# Mid-Infrared Transparent Materials: from Mechanisms to Cutting-Edge Applications

**DOI:** 10.1007/s40820-026-02113-y

**Published:** 2026-02-28

**Authors:** Hanyuan Zhang, Zhenhui Huang, Changyuan Chen, Yi Long, Weilin Xu, Zhengui Zhou, Jun Wan

**Affiliations:** 1 State Key Laboratory of New Textile Materials and Advanced Processing, School of Chemistry and Chemical Engineering, Wuhan Textile University, Wuhan, 430200 People’s Republic of China; 2 Department of Electronic Engineering, The Chinese University of Hong Kong, New Territories, Hong Kong SAR, 999077 People’s Republic of China

**Keywords:** Mid-infrared transparent materials, Imaging and sensing, Laser systems, Thermal management, Optical applications

## Abstract

The mid-infrared (MIR) spectral window, typically spanning wavelengths from 2.5 to 20 μm (or wave numbers 500–4000 cm^−1^), constitutes a pivotal domain of the electromagnetic spectrum, where molecular vibrational and rotational transitions enable precise spectroscopic identification and tunable thermal radiation modulation. Mastery over this spectral range underpins a broad and growing suite of technologies, encompassing high-resolution MIR imaging and spectroscopic gas sensing, advanced thermal management via radiative cooling/heating and dynamic emissivity control, integrated photonic platforms featuring low-loss optical windows and waveguides, as well as MIR laser systems that leverage broadband transparency for efficient frequency conversion and beam delivery. High MIR transmittance (*T*_MIR_) is therefore essential for driving MIR photonic innovations, enabling efficient photon transmission, modulation, and targeted heat control. Yet, the fundamental interplay among material structure, photonic/electronic behavior, and MIR optical performance remains underexplored. This review comprehensively evaluates high *T*_MIR_ materials, with an emphasis on their optical mechanisms, structural attributes, synthesis routes, and performance benchmarks. By elucidating structure–property relationships and offering design strategies for MIR transparency, this review provides a roadmap for developing high-performance MIR transparent materials for advanced thermal management, infrared optics, and next-generation photonic systems.

## Highlights

- Establishes a structure–property–function paradigm linking band structure, phonons, and architecture to mid-infrared (MIR) transmittance.
- Provides a rigorous taxonomy spanning intrinsic and engineered materials with clarified transparency mechanisms.
- Derives design rules enabling low-loss MIR transport for advanced thermal and photonic systems.

## Graphical abstract

**Figure:**
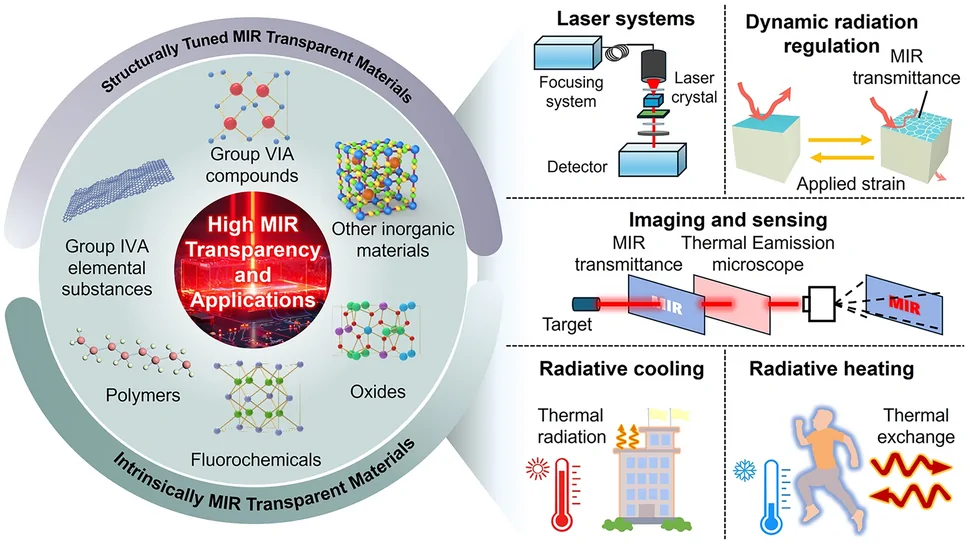

## Introduction

The mid-infrared (MIR) spectral region (2.5–20 μm) occupies a critical position within the electromagnetic spectrum, where fundamental interactions such as molecular vibrations, lattice phonon resonances, and thermal radiation processes converge. This spectral domain underpins a diverse array of cutting-edge technologies spanning energy conversion, environmental monitoring, defense systems, and advanced optical platforms [1–7]. Within this context, materials with high MIR transmittance (*T*_MIR_) have emerged as indispensable functional platforms, bridging fundamental light–matter interactions with practical engineering demands. Their ability to transmit, modulate, and convert MIR photons enables versatile integration across disciplines, supporting the development of next-generation systems in photonics, thermal control, and optoelectronics [8–11]. From molecular fingerprinting in infrared spectroscopy to dynamic thermal modulation in complex environments, high *T*_MIR_ materials offer a unique foundation for achieving simultaneous optical and thermal functionality [12–21].

In optical systems, these materials serve as the foundational components of MIR photonics, including laser gain media, waveguides, and sensor substrates, where their low intrinsic optical losses and compatibility with nonlinear optical processes are critical to device performance [22–27]. Simultaneously, their radiative properties facilitate precise thermal control via selective infrared emission or transmission, making them highly valuable for both passive and active thermal management systems [28–30]. This dual functionality originates from the intrinsic coupling among the electronic structure, phonon behavior, and microstructural organization, which determine MIR transparency and spectral emissivity simultaneously [31, 32]. Recent advances in material design strategies, such as microstructural engineering, compositional tuning, and hybrid integration, have greatly expanded the performance range of high *T*_MIR_ materials, creating new opportunities for multifunctional integration and system-level innovation. Among multiple use cases, two application domains best exemplify the high potential of high *T*_MIR_ materials. First, in thermal management, their ability to selectively transmit or emit infrared radiation has enabled breakthroughs in radiative cooling manipulation, solar heating, and hybrid photothermal management [33–35]. Acting as spectrally selective interfaces, these materials minimize unwanted heat exchange while preserving desirable optical transparency, which is essential for applications such as adaptive building façades, wearable thermal devices, and aerospace insulation systems [36–38]. Second, in optical and photonic technologies, high *T*_MIR_ materials are integral to high-resolution thermal imaging, gas sensing, hyperspectral detection, and integrated MIR photonics. Their broadband transparency and low optical loss significantly enhance signal fidelity, miniaturization, and operational efficiency [39–51].

Despite their broad potential, the practical implementation of high *T*_MIR_ materials is still hindered by intrinsic trade-offs among optical transmittance, thermal stability, mechanical durability, and manufacturability. For instance, La_0.15_Y_1.85_O_3_ exhibits improved hardness with La^3+^ substitution, but suffers from reduced thermal stability [52]. Y_2_O_3_–MgO nanocomposites, widely explored for infrared ceramics, suffer from excessive phonon scattering and MIR absorption losses [53]. While elemental semiconductors such as silicon (Si) and germanium (Ge) display excellent *T*_MIR_, they are limited by low mechanical durability [54]. Similarly, high-sulfur polymers offer promising optical properties but are limited by poor thermal and mechanical resilience, affecting their reliability in demanding applications [55]. Moreover, some high-performance MIR materials such as Ge and chalcogenide glasses face limitations due to high production costs, scarce raw material resources, and complex fabrication processes [56–60]. These limitations highlight the urgent need to deepen the understanding of the relationships among structure, properties, and performance that determine *T*_MIR_ behavior, in order to guide the development of next-generation materials with high optical efficiency, mechanical robustness, thermal stability, and scalability. However, current literature remains fragmented, with insufficient emphasis on the structural and compositional determinants of *T*_MIR_ performance across different material classes [61].

**Fig. 1:**
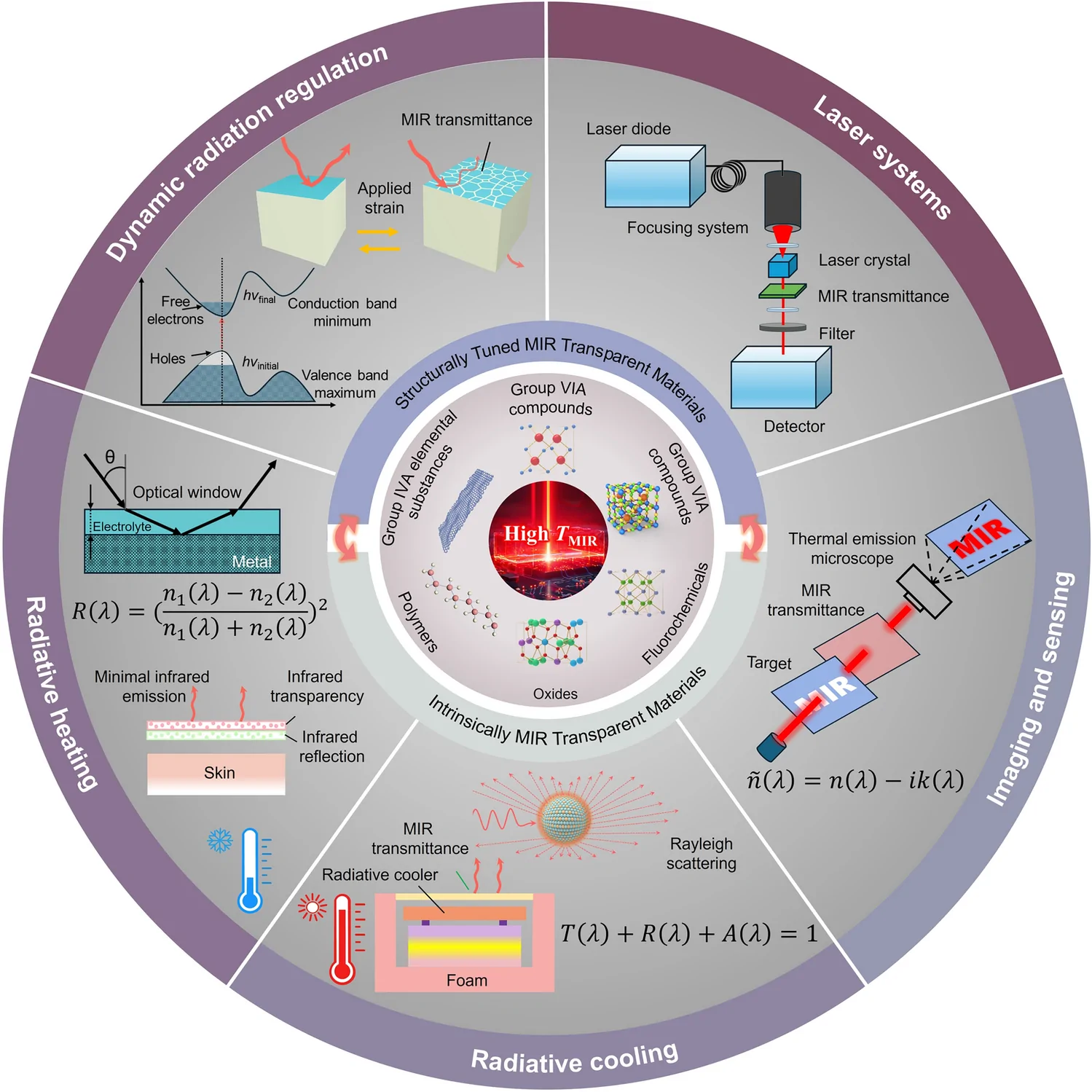
Overview of high *T*_MIR_ materials and their applications in optical technologies and thermal management

To address these gaps, this review presents a comprehensive and material-focused examination of the current landscape of high *T*_MIR_ materials. Through critical evaluation of intrinsic mechanisms, microstructural manipulation, synthesis methodologies, and application-specific performance metrics, this review seeks to establish a unified framework for understanding and optimizing MIR transparent materials. The discussion begins with a concise overview of the fundamental principles governing *T*_MIR_, followed by a classification of representative high *T*_MIR_ materials (Fig. 1), distinguishing between inherently transparent systems and those engineered through structural modification. Their key roles in emerging applications are systematically analyzed, including optical windows for hyperspectral imaging, selective radiative cooling and heating, MIR thermal modulation, and low-loss substrates for integrated photonics. Further attention is devoted to their potential as MIR transparent electrodes in laser systems and gas sensors. Finally, the review identifies critical materials and process challenges and outlines future directions aimed at enabling broader deployment of high *T*_MIR_ materials in next-generation energy-efficient, photonic, and optoelectronic technologies.

### Fundamental Principles Governing MIR Transmittance

To systematically elucidate the origins and design principles governing *T*_MIR_, this section is divided into two interrelated parts. The first part outlines the fundamental optical principles that determine *T*_MIR_, highlighting its dependence on intrinsic material parameters (*e.g.*, refractive index, extinction coefficient), interfacial boundary conditions, and wave propagation dynamics. The second part focuses on the principal attenuation mechanisms of MIR transmission, including intrinsic vibrational absorption and extrinsic scattering processes, and further analyzes how these are modulated by compositional attributes and microstructural features. This comprehensive framework provides the physical foundation for evaluating *T*_MIR_ and guiding the rational design of high *T*_MIR_ materials.

#### Optical Fundamentals of MIR Transmittance

##### Energy Balance and Definitions Insertion

**Fig. 2:**
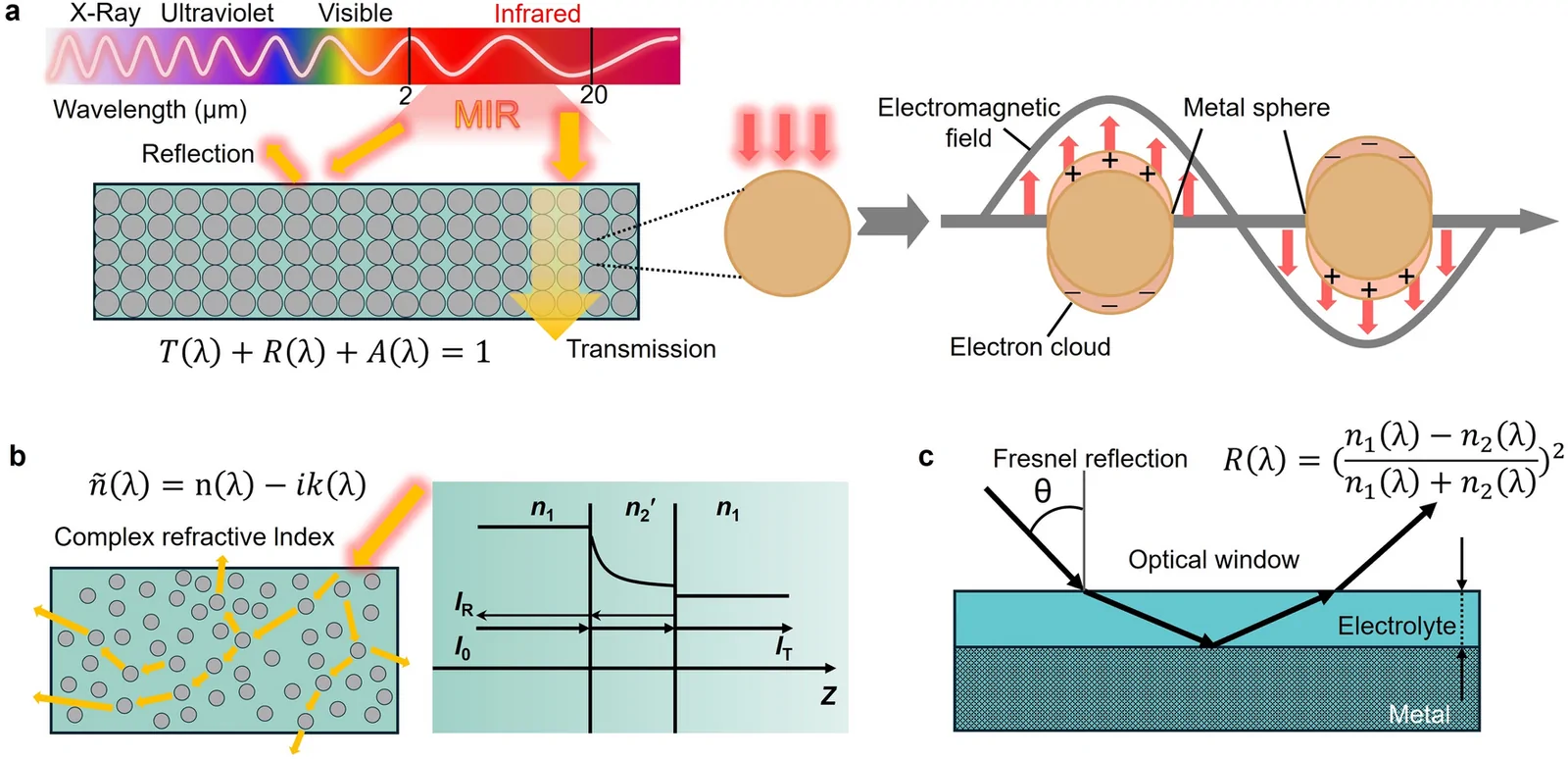
Schematic illustration of mid-infrared transmittance phenomena.** a** Schematic of MIR transparent materials showing transmission, reflection, and internal absorption pathways. **b** Beer–Lambert model illustrating exponential attenuation of light through an absorbing medium. **c** Fresnel reflection at an interface, depicting angle-dependent partial reflection and transmission due to refractive index contrast

In the MIR spectral window, the transmittance of a material is governed by the fundamental principles of radiative energy conservation (Fig. 2a). For a non-luminescent, linear, and homogeneous medium exposed to monochromatic MIR radiation under normal incidence, the incident energy is partitioned among three primary optical channels: transmission through the medium, reflection at the interface, and internal absorption. This relationship can be expressed as:

1 $$\begin{array}{*{20}c} {T\left( \lambda \right) + R\left( \lambda \right) + A\left( \lambda \right) = 1} \\ \end{array}$$

where $T\left(\lambda \right)$ is the spectral transmittance, $R\left(\lambda \right)$ is the reflectance, and $A\left(\lambda \right)$ is the absorptance, each as a function of wavelength λ∈[2.5, 20 μm]. This identity holds across the MIR region and provides the quantitative foundation for optimizing optical transparency in this range. $T\left(\lambda \right)$ describes the fraction of incident MIR radiation that emerges from the rear surface of the material without being absorbed or reflected. In practical measurements, transmittance encompasses both specular and diffuse contributions, and may be angle-dependent in materials with anisotropic microstructures or surface roughness [62–64]. $R\left(\lambda \right)$ results from the discontinuity in refractive index at the air–material interface and accounts for the portion of incident energy that is redirected at the surface without entering the material. In the MIR regime, materials with high refractive indices (*e.g.*, Ge, Si, or chalcogenide glasses with n > 3) typically exhibit strong reflection losses unless mitigated by surface engineering or antireflective coatings [65, 66]. For such systems, $R\left(\lambda \right)$ may exceed 30% even at normal incidence if left uncorrected, significantly degrading the effective *T*_MIR_. $A\left(\lambda \right)$ encompasses all internal attenuation mechanisms, including phonon-induced vibrational absorption, free carrier damping, interband transitions, and any scattering losses that redirect energy away from the forward transmission path [67–69]. To maintain a consistent loss hierarchy, extrinsic losses associated with scattering, denoted here as $S\left(\lambda \right)$, originate from surface roughness, pores, grain boundaries, and microscale inhomogeneities that redirect energy away from the forward direction; while Rayleigh scattering is generally weak in the MIR, Mie scattering from microscale heterogeneities can still be non-negligible and should be treated as a distinct extrinsic loss channel when interpreting measured *T*_MIR_. In the 2.5–20 μm range, absorption is often dominated by multiphonon processes and vibrational overtones in ionic crystals, or by localized vibrational bands in polymers [70]. Importantly, while Rayleigh scattering is typically negligible in the MIR due to the long wavelengths involved, Mie scattering from microscale inhomogeneities may still contribute to effective absorptance in real systems and must be considered under $A\left(\lambda \right)$.

For high *T*_MIR_ materials, the design objective is to minimize both R(λ) and A(λ) across a defined spectral subwindow within 2.5–20 μm. It is important to note that a material does not need to be transparent across the entire MIR band to be functionally effective; instead, application-specific windows (*e.g.*, 3–5 μm or 8–13 μm atmospheric windows) often define the critical operating range [67, 71]. Accordingly, the spectral dependence of T(λ) must be interpreted with respect to both the material’s intrinsic optical response and the application’s spectral requirements. In addition, the total integrated transmittance over a given spectral interval [${\lambda }_{1}$, ${\lambda }_{2}$] is frequently used as a figure of merit for MIR window performance:

2 $$\begin{array}{*{20}c} {\overline{T} = \frac{1}{{\lambda_{2} - \lambda_{1} }}\mathop \smallint \limits_{{\lambda_{1} }}^{{\lambda_{2} }} T\left( \lambda \right)d\lambda } \\ \end{array}$$

This metric allows for direct comparison between different materials or processing conditions and is particularly useful when evaluating broadband transmission in materials such as IR ceramics or polymeric films. However, when using this integral, care must be taken to weight T(λ) appropriately in systems with non-uniform spectral intensity (*e.g.*, thermal emission sources or tunable MIR lasers).

Finally, it is worth emphasizing that the energy balance equation applies under linear, steady-state conditions and may not capture dynamic or nonlinear optical phenomena relevant under high-intensity MIR laser excitation. However, within the context of most passive optical applications, including imaging, sensing, and thermal management, this energy conservation framework provides a robust and essential starting point for evaluating and engineering high-performance MIR transparent materials.

##### Complex Refractive Index and Beer–Lambert Relation

The propagation of MIR radiation through a material is fundamentally governed by its complex refractive index, which encapsulates both the phase velocity and attenuation characteristics of light within the medium (Fig. 2b). This complex quantity is defined as:

3 $$\begin{array}{*{20}c} {\tilde{n}\left( \lambda \right) = n\left( \lambda \right) - ik\left( \lambda \right)} \\ \end{array}$$

where n(λ) is the real part of the refractive index, dictating the speed and refraction of light, and k(λ) is the extinction coefficient, associated with exponential attenuation of the wave due to intrinsic absorption [72–74]. Both components are wavelength-dependent and are particularly critical in the 2.5–20 μm range, where materials often exhibit strong phonon-related absorption features and dispersive behavior [72, 75, 76]. When an MIR beam traverses a homogeneous medium of thickness *d*, the intensity of the transmitted wave I(λ) relative to the incident intensity ${I}_{0}\left(\lambda \right)$ follows the Beer–Lambert law:

4 $$\begin{array}{*{20}c} {T\left( \lambda \right) = \frac{I\left( \lambda \right)}{{I_{0} \left( \lambda \right)}} = \exp \left( { - \alpha \left( \lambda \right)d} \right)} \\ \end{array}$$

Here, the absorption coefficient $\alpha (\lambda )$ is directly related to the extinction coefficient via:

5 $$\begin{array}{*{20}c} {\alpha \left( \lambda \right) = \frac{4\pi k\left( \lambda \right)}{\lambda }} \\ \end{array}$$

By substitution, the fundamental relation for spectral transmittance in the MIR region can be expressed as:

6 $$\begin{array}{*{20}c} {T\left( \lambda \right) = \exp \left( { - \frac{4\pi k\left( \lambda \right)d}{\lambda }} \right)} \\ \end{array}$$

This expression highlights the exponential sensitivity of transmittance to both the extinction coefficient and the sample thickness. In the MIR regime, where λ is typically in the tens of micrometers, even relatively small values of k(λ)∼10^−3^ can significantly reduce transmittance over millimeter-scale path lengths. Consequently, for materials to exhibit high *T*_MIR_, it is essential that the extinction coefficient k(λ) remains extremely low, ideally less than 10^−4^, across the entire operational spectral range. The functional form of k(λ) is determined by intrinsic material properties, including lattice vibrations (in ionic solids), overtones of molecular vibrational modes (in polymers), and free carrier absorption (in doped semiconductors). These mechanisms give rise to pronounced absorption peaks or broad bands, often centered within the MIR [77]. Therefore, accurate determination of k(λ) is essential in evaluating a material’s suitability for MIR transmission applications.

Practically, n(λ) and k(λ) can be extracted via spectroscopic ellipsometry, Kramers–Kronig transformation of reflectance spectra, or direct inversion of transmittance–reflectance data using models such as the Lorentz oscillator formalism [78–80]. In engineering contexts, minimizing k(λ) is achieved not only through careful material selection but also via precise process control, such as reducing impurity concentrations, eliminating microstructural heterogeneity, or tuning vibrational modes to lie outside the target spectral range. Notably, in the MIR region, refractive index dispersion is often significant, especially in proximity to phonon absorption bands [81]. As n(λ) increases, so too does Fresnel reflection at interfaces, which can further suppress effective transmittance. Thus, the real and imaginary parts of $\widetilde{n}$ are intricately coupled in determining both transmission losses and optical matching strategies, especially in multilayer systems or antireflective coating designs.

In summary, the Beer–Lambert relation, grounded in the material’s complex refractive index, offers a quantitative basis for understanding and predicting *T*_MIR_. For a material to be considered genuinely transparent in the 2.5–20 μm window, it must exhibit low and spectrally flat k(λ), together with controlled n(λ) that minimizes reflection losses and enables coherent optical integration. These criteria form the foundation of rational material selection and design in MIR transparent systems.

##### Fresnel Reflection and Antireflective Considerations

In addition to intrinsic absorption, Fresnel reflection at material interfaces represents a significant source of transmittance loss in the MIR region, especially when dealing with high-refractive-index materials (Fig. 2c) [82]. At each dielectric interface, part of the incident MIR wave is reflected due to discontinuity in optical impedance. This effect is quantitatively described by the Fresnel equations, which, under normal incidence, yield the reflectance *R* as:

7 $$\begin{array}{*{20}c} {R\left( \lambda \right) = \left( {\frac{{n_{1} \left( \lambda \right) - n_{2} \left( \lambda \right)}}{{n_{1} \left( \lambda \right) + n_{2} \left( \lambda \right)}}} \right)^{2} } \\ \end{array}$$

where ${n}_{1}\left(\lambda \right)$ and ${n}_{2}\left(\lambda \right)$) are the real parts of the refractive indices of the adjacent media at wavelength λ. In a typical configuration involving an air–material interface (${n}_{1}\sim$ 1), the reflectance increases with the refractive index of the substrate [83]. For materials such as germanium (Ge, n∼4.0) or chalcogenide glasses (n∼2.5–3.5) in the 2.5–20 μm range, uncoated surface reflectance at normal incidence can exceed 25%–35%, thereby significantly degrading the effective transmittance even if internal absorption is minimal. Moreover, the reflectance is wavelength-dependent due to the dispersion of n(λ), which can be particularly pronounced in the MIR region where phonon resonances or lattice polarizability variations occur [84, 85]. In highly dispersive materials, this leads to spectral non-uniformity in reflection loss, complicating optical system design and limiting the utility of such materials without surface modification.

To address this challenge, antireflective (AR) strategies are essential to approach the theoretical transmission limits of bulk materials. A classic solution involves introducing a single-layer AR coating whose refractive index *n*_c_ satisfies the geometric mean condition:

8 $$\begin{array}{*{20}c} {n_{c} \left( \lambda \right) = \sqrt {n_{s} \left( \lambda \right)} } \\ \end{array}$$

where ${n}_{s}$ is the refractive index of the substrate material, assuming air as the incident medium. For destructive interference to occur at the target wavelength ${\lambda }_{0}$, the optical thickness of the coating must be one-quarter wavelength:

9 $$d = \frac{{\lambda_{0} }}{{4n_{c} }}$$

This quarter-wave AR condition results in minimal reflectance at ${\lambda }_{0}$ and can be extended into the MIR using materials such as thorium fluoride (ThF_4_), zinc sulfide (ZnS) [86], or certain low-index polymers like fluorinated polyimides, which remain transparent in portions of the 2.5–20 μm range. However, the narrowband nature of single-layer coatings limits their effectiveness for broadband MIR applications.

To overcome this limitation, multilayer AR stacks or gradient index structures are employed, enabling reduced reflectance across broader MIR bands. These may involve combinations of high- and low-index layers (*e.g.*, Ge/ZnSe, ZnS/CaF_2_, or Si/SiO_2_) [72], designed via optical transfer matrix modeling to tailor destructive interference across the desired spectral range. In some cases, nanoporous coatings or sol–gel-derived mesostructures can approximate a continuous refractive index gradient, yielding effective impedance matching with minimal chromatic artifacts. Another effective strategy is surface texturing, in which subwavelength-scale structures such as pyramids, moth-eye arrays, or etched gratings are patterned on the material surface to enable a gradual transition of the effective refractive index from air to the substrate. This approach, when realized with feature sizes below the shortest wavelength of interest (i.e., < 2.5 μm), can significantly suppress Fresnel reflection across the full MIR band. However, fabrication complexity and long-wavelength diffraction must be considered, especially in flexible or non-planar systems. It is also noteworthy that for thin-film materials or composite coatings, interference effects between front and rear interfaces may either enhance or suppress transmittance depending on layer thickness and coherence length. In such cases, precise control over film thickness and surface planarity is required to avoid unintended Fabry–Pérot resonances within the MIR region, particularly in materials with low absorption coefficients where interference fringes are spectrally resolvable [87].

Therefore, Fresnel reflection constitutes a non-negligible loss channel in MIR transparent systems and must be systematically mitigated through refractive index engineering at material interfaces [88]. Whether via discrete-layer coatings, graded-index designs, or nanostructured surfaces, the choice of AR strategy must consider spectral bandwidth, thermal stability, mechanical robustness, and compatibility with the optical and chemical properties of the underlying substrate. For MIR transparent materials to realize their full potential in optical and thermal applications, surface optimization is as crucial as bulk transparency.

#### Attenuation Mechanisms and Microstructural Influences on MIR Transmittance

While minimizing intrinsic absorption is a primary objective in achieving high *T*_MIR_, practical materials inevitably exhibit a range of attenuation phenomena that compromise optical clarity. These losses arise from both fundamental interactions, such as photon coupling with vibrational and electronic excitations, and extrinsic structural factors, including grain boundaries, porosity, and compositional inhomogeneity [89–91]. In the 2.5–20 μm range, where wavelengths are particularly sensitive to phonon absorption and mesoscopic scattering, the interplay between intrinsic optical properties and microstructural architecture becomes especially critical. This section provides a comprehensive examination of the physical origins of attenuation in MIR transparent materials, highlighting the mechanisms by which absorption and scattering arise and the structural factors that exacerbate or suppress these effects. Understanding these interconnected pathways is essential for tailoring material compositions, refining processing strategies, and ultimately advancing the design of low-loss MIR optical components.

##### Intrinsic Absorption Mechanisms

**Fig. 3:**
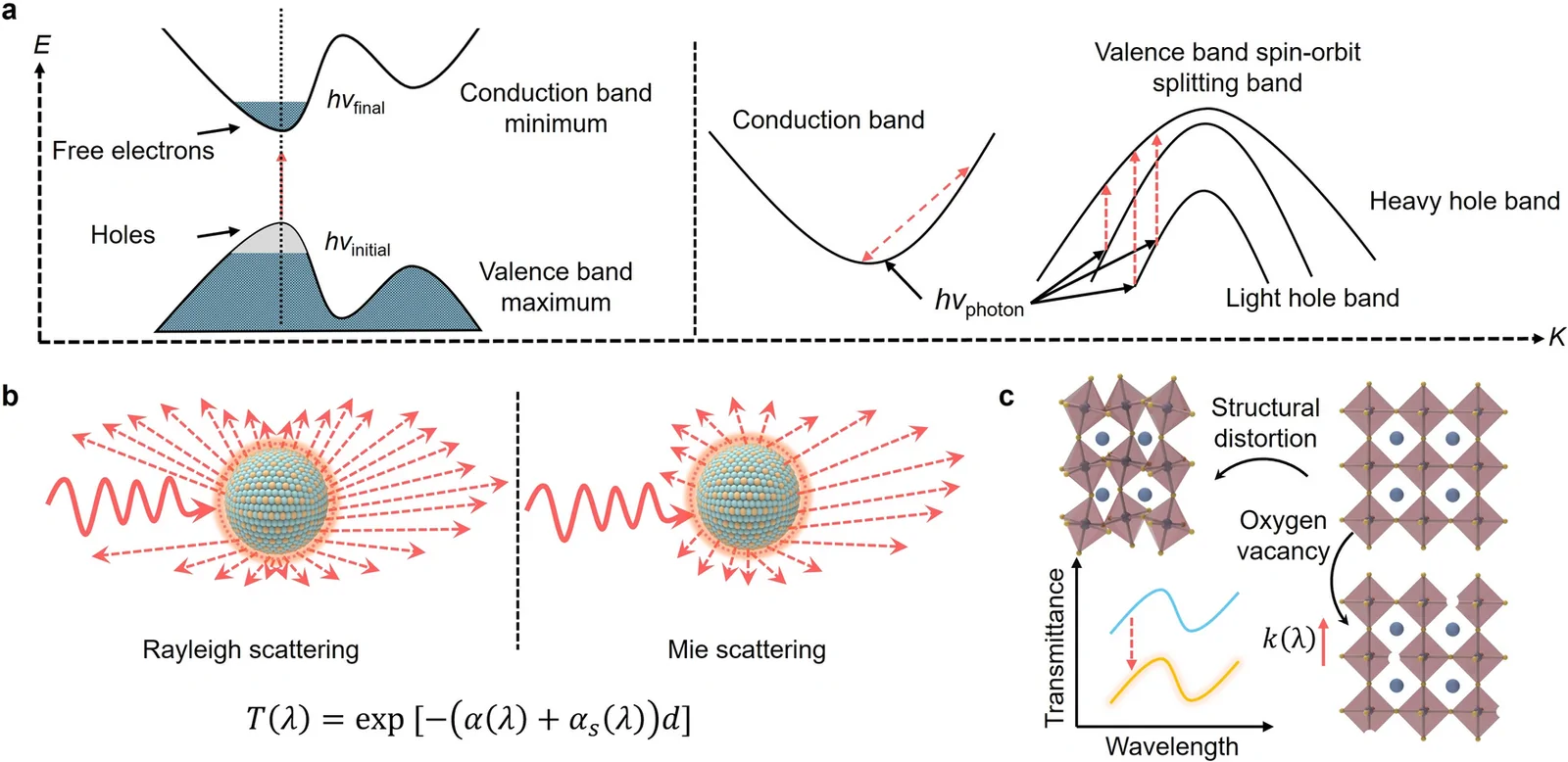
Schematic illustration of mid-infrared transmittance phenomena.** a** Intrinsic electronic transitions contributing to free carrier absorption and interband attenuation. **b** Rayleigh and Mie scattering effects. **c** Influence of lattice distortions and oxygen vacancies on mid-infrared transmittance

Intrinsic absorption represents a fundamental limitation to achieving high *T*_MIR_, where photon energies (∼0.06–0.5 eV) interact with the material’s vibrational and electronic states. In MIR transparent media, the dominant attenuation arises from lattice vibrations, electronic subgap transitions, and free carrier effects, each of which introduces wavelength-selective loss (Fig. 3a) [92].

In polar dielectrics and ionic crystals, strong coupling between the electric field of the incident MIR wave and optical phonon modes leads to characteristic absorption bands. These phonon-mediated absorptions occur when the photon frequency matches the vibrational resonance of the lattice, typically in the form of transverse optical (TO) and longitudinal optical (LO) modes. The dielectric response in this regime is well described by the Lorentz oscillator model:

10 $$\begin{array}{*{20}c} {\varepsilon \left( \omega \right) = \varepsilon_{\infty } + \mathop \sum \limits_{j} \frac{{S_{j} }}{{\omega_{0j}^{2} - \omega^{2} - i\gamma_{j} \omega }}} \\ \end{array}$$

where ${\varepsilon }_{\infty }$ is the high-frequency dielectric constant, ${S}_{j}$ is the oscillator strength, ${\omega }_{0j}$ is the phonon resonance frequency, and ${\gamma }_{j}$ is the damping factor. The imaginary part of the dielectric function determines the extinction coefficient k(λ), which governs the absorption coefficient $\alpha (\lambda )$ [93]. In practical applications, materials with strong phonon absorption, such as many metal oxides, tend to exhibit low transmittance in the vicinity of their Reststrahlen bands, which are typically situated between 10 and 20 μm [94]. This restricts their use in MIR applications. Conversely, materials with low vibrational polarity and simple unit cells, such as fluorides or nitrides, can exhibit broader transparency windows due to weaker dipole-induced absorption.

Beyond vibrational losses, subgap electronic absorption also contributes to MIR attenuation, particularly in materials containing structural disorder, defects, or impurity states [95]. Although direct band-to-band transitions generally lie outside the MIR range due to wide bandgaps (typically > 3 eV), residual absorption can arise from localized states within the gap. These include Urbach tails, defect bands, or transitions assisted by phonons. The resulting absorption spectra often exhibit weak but broadband exponential edges, particularly evident in disordered systems or amorphous materials. This behavior can be empirically described by an Urbach-type relation, where $\alpha (\lambda )$ increases with wavelength:

11 $$\begin{array}{*{20}c} {\alpha \left( \lambda \right) \propto \exp \left( {\frac{hc}{{\lambda E_{U} }}} \right)} \\ \end{array}$$

${E}_{U}$ denoting the Urbach energy that quantifies the degree of tailing from structural disorder. Minimizing such contributions requires high structural order, stoichiometric precision, and post-synthetic thermal treatments that anneal defect states and reduce absorption in the MIR window.

Another significant intrinsic loss channel arises from free carrier absorption, particularly in doped semiconductors or conductive ceramics [96]. Free carriers absorb MIR photons through intraband transitions, which are described by the classical Drude model. The wavelength dependence of this mechanism is quadratic, making it especially detrimental at longer MIR wavelengths. The Drude-type absorption coefficient can be approximated as:

12 $$\begin{array}{*{20}c} {\alpha \left( \lambda \right) \propto \frac{{N\lambda^{2} }}{{m^{*} t}}} \\ \end{array}$$

where *N* is the carrier concentration, *m* ∗ is the effective mass, and* t* is the carrier scattering time. Even low levels of unintentional doping or intrinsic carrier generation can introduce noticeable absorption at wavelengths above 10 μm. Therefore, maintaining ultra-low carrier densities is critical in materials intended for MIR transparency.

Additionally, impurity-induced absorption from residual chemical groups or extrinsic ions further limits *T*_MIR_. For instance, –OH, –CH, and –CO groups exhibit overtone or combination vibrational modes that strongly absorb in the 2.5–8 μm region. These modes are particularly relevant in polymeric systems, sol–gel-derived oxides, or hybrid organic–inorganic materials [97]. Typical hydroxyl-related absorption near 2.7–3.5 μm or carbonyl absorption in the 5–6 μm range can drastically reduce transmittance if not properly mitigated. Achieving high MIR transparency in such systems thus necessitates rigorous precursor purification, solvent-free processing, and post-synthetic dehydration or annealing.

Overall, intrinsic absorption in MIR transparent materials results from a complex interplay of lattice dynamics, electronic structure, and defect chemistry. The resulting extinction coefficient k(λ), through the Beer–Lambert relation, exponentially suppresses transmittance with increasing thickness or defect density. Consequently, optimizing *T*_MIR_ demands a combined strategy: selecting materials with weak phonon activity, wide bandgaps, and low free carrier densities, while also engineering synthesis protocols that eliminate mid-gap states and molecular contaminants. Only by managing these intrinsic factors can materials achieve the low-loss performance required for advanced MIR optical and thermal applications.

##### Scattering Mechanisms

In the MIR region, where photon wavelengths range from 2.5 to 20 μm, optical scattering represents a critical form of transmittance attenuation that coexists with intrinsic absorption (Fig. 3b). Unlike absorption, which results from direct energy conversion into vibrational or electronic excitations, scattering redistributes incident light through redirection, diffraction, or diffusion, thereby reducing the forward-propagating intensity measurable as transmittance. The physical origin of scattering lies in spatial fluctuations of dielectric permittivity within the material, which can result from structural heterogeneities such as grain boundaries, pores, inclusions, or surface irregularities [98]. The impact of these scattering sources on *T*_MIR_ depends strongly on their size, distribution, and refractive index contrast relative to the host matrix, particularly in comparison to the MIR wavelength.

For inhomogeneities much smaller than the incident wavelength (i.e., feature size d ≪ λ), scattering is well described by Rayleigh theory, in which the scattering cross section *σ*_s_ scales as *d*^6^/*λ*^4^. As a result, Rayleigh scattering becomes increasingly negligible at longer wavelengths, making it a relatively minor contributor in the 10–20 μm region [99]. However, in materials with high densities of nanoscale voids, secondary phase nanoparticles, or compositional inhomogeneities, this mechanism may still contribute to modest transmission losses near the short-wavelength end of the MIR window. The extent of Rayleigh scattering can be minimized by reducing the density of sub-100 nm inclusions and ensuring compositional homogeneity during synthesis or processing. When the characteristic feature size of the scattering centers approaches or exceeds the wavelength (*i.e.*, *d*∼*λ*), Mie scattering dominates. This regime is particularly relevant in polycrystalline ceramics, porous media, or nanocomposites where the grain sizes or embedded particles range from 1 to 10 μm, which is comparable to MIR wavelengths. Unlike Rayleigh scattering, Mie scattering exhibits complex angular and spectral dependencies, with forward-scattering dominance and oscillatory behavior that depends on the size parameter $x=2\pi r/\lambda$ and the relative refractive index of the inclusion to the matrix. The total scattering efficiency is strongly enhanced when the refractive index contrast is large and the scatterer is resonant with incident MIR radiation. Materials that are highly porous, multiphase, or poorly sintered frequently exhibit significant Mie scattering, especially in the atmospheric window critical to many infrared applications. Grain boundary and interface scattering further impact *T*_MIR_, particularly in polycrystalline or layered materials. In well-densified systems, the optical discontinuity across grain boundaries can still produce weak scattering if adjacent crystallites exhibit misorientation or different optical anisotropies. Moreover, residual pores and microcracks at boundaries can act as localized scattering centers, especially when their dimensions lie in the subwavelength to few-micron range. In multilayer systems or composites, abrupt interfaces with mismatched optical impedance lead to interfacial scattering that may degrade directional transmittance even if individual layers are nominally transparent [100]. For MIR optical elements such as windows, lenses, or coatings, such interface-induced scattering becomes particularly detrimental when coherent or collimated radiation is required [101].

To quantify this impact more rigorously, scattering should be treated as a consequence of structural length scales and dielectric discontinuities relative to the operating wavelength, because these parameters determine how radiative energy is redistributed in angle rather than simply attenuated. This immediately motivates a distinction between directional (ballistic) transmittance and hemispherical (total) transmittance: index mismatches, interfaces, and inclusions with characteristic sizes comparable to the wavelength can strongly suppress ballistic throughput by transferring energy into off axis directions, while the total transmitted energy may remain relatively high if absorption is low. Such a separation is essential at the device level, since imaging and coherent beam delivery demand high ballistic transmittance and low haze, whereas many non-imaging implementations can tolerate larger diffuse contributions provided that the integrated throughput is preserved. Accordingly, diffuse transport in strongly scattering media should not be represented by a single Beer–Lambert-type factor, but instead assessed through metrics that separate ballistic and diffuse components, consistent with the underlying wavelength-dependent scattering constraints.

In summary, scattering mechanisms in MIR transparent materials are intrinsically linked to structural length scales and dielectric discontinuities relative to the operating wavelength. The spectral range of 2.5–20 μm, with its broad variation in photon wavelength, imposes strict constraints on permissible inhomogeneity sizes and refractive index mismatches [102]. Therefore, successful material platforms must integrate optical design with microstructural engineering to suppress all relevant scattering contributions and unlock the full potential of broadband MIR transparency.

##### Microstructural and Compositional Factors

Beyond the intrinsic optical constants of a material, its actual *T*_MIR_ performance is strongly governed by microstructural and compositional attributes. Even in systems with intrinsically low absorption and refractive index-matched interfaces, imperfections in the structural arrangement or chemical composition can induce additional absorption and scattering losses, particularly within the spectral window where the optical response is highly sensitive to vibrational and dielectric heterogeneity (Fig. 3c).

The crystal structure plays a foundational role in shaping the phonon modes and determining the position and intensity of infrared-active vibrations. Highly symmetric and centrosymmetric lattices, such as those found in cubic fluorides or garnet-type oxides, typically exhibit fewer infrared-active phonon modes and possess broader transparency windows. In contrast, structures with lower symmetry and more complex unit cells often support numerous strong dipole-active vibrations, which can introduce absorption bands within the MIR region. Thus, materials with minimal asymmetry in bond polarizability and reduced mode density are generally favorable for MIR transparency. Moreover, structural phase transitions (*e.g.*, from cubic to monoclinic) can activate otherwise forbidden vibrational modes, leading to sudden transmittance degradation over specific subbands.

Grain size and crystallographic orientation exert a dual influence on both scattering and phonon dynamics. In polycrystalline ceramics or films, large grain sizes reduce the density of grain boundaries and thereby suppress boundary-related scattering [103]. However, if the grain sizes become comparable to the MIR wavelength, particularly within the range of 2–10 μm, Mie scattering can become significant, as previously discussed. To mitigate this, processing techniques such as hot pressing or spark plasma sintering are employed to promote grain coarsening while maintaining high optical density [104]. Additionally, materials with anisotropic optical properties may exhibit orientation-dependent *T*_MIR_, especially in uniaxial crystals. In such cases, controlled texturing, such as aligning the optical axis with the direction of the incident beam, can improve effective transmission by favoring orientations that promote transparency.

Point defects and compositional disorder introduce localized states within the bandgap or perturb the vibrational structure, both of which contribute to MIR absorption. Oxygen vacancies, cation non-stoichiometry, or substitutional impurities can activate silent modes or broaden existing phonon features, increasing the extinction coefficient k(λ). Defect-related scattering may also arise due to strain fields or local dielectric contrast [105]. The concentration and distribution of such defects must be minimized through careful precursor selection, controlled atmosphere sintering, and thermal annealing [106]. For example, the presence of hydroxyl groups, residual carbon, or halide residues in oxide and fluoride ceramics can give rise to vibrational absorption near 2.7–3.5 or 5–7 μm, respectively, significantly compromising transmittance in those ranges.

Porosity is another critical parameter. Even when pores are not optically absorbing, they cause scattering due to the mismatch in refractive index between the void and the surrounding matrix. The scattering cross section is most pronounced when the pore size is on the order of the MIR wavelength, which includes a wide range from submicrometers to tens of micrometers. Achieving high-density microstructures through pressure-assisted sintering, controlled binder burnout, or sol–gel densification is therefore essential to minimizing optical losses. In addition to bulk porosity, microcracks or intergranular voids introduced during cooling or mechanical processing can produce localized refractive index gradients that scatter and diffract MIR radiation [107].

Surface morphology also affects transmittance, especially at shorter MIR wavelengths. Surface roughness on the scale of 100 nm–1 μm can lead to significant diffuse reflection, reducing effective *T*_MIR_. This is particularly important for thin films and optical components such as windows or domes, where even small deviations from flatness or uniformity can introduce angular dispersion or beam distortion. Optical-grade polishing or atomic layer planarization may be required to meet surface roughness thresholds for minimal MIR scattering. Moreover, surface contamination such as adsorbed water or organic residues can introduce molecular vibrational modes in the MIR region, potentially overlapping with the desired transparency window. This necessitates stringent post-processing measures and storage in inert or dry environments.

In sum, the microstructural and compositional architecture of a material dictates not only its mechanical and thermal robustness, but also the fine-scale optical behavior that determines its usefulness as a MIR transparent medium [108]. Achieving high *T*_MIR_ requires integrated design across multiple length scales: from the atomic ordering that defines phonon spectra, to the grain and pore structures that influence scattering, and finally to surface and interface engineering that preserves coherent transmission. As such, microstructural refinement is not merely a supporting element but a central determinant of performance in high-efficiency MIR optical platforms.

## Classification of High *TMIR* Materials

### Overview of Representative High *TMIR* Materials

**Fig. 4:**
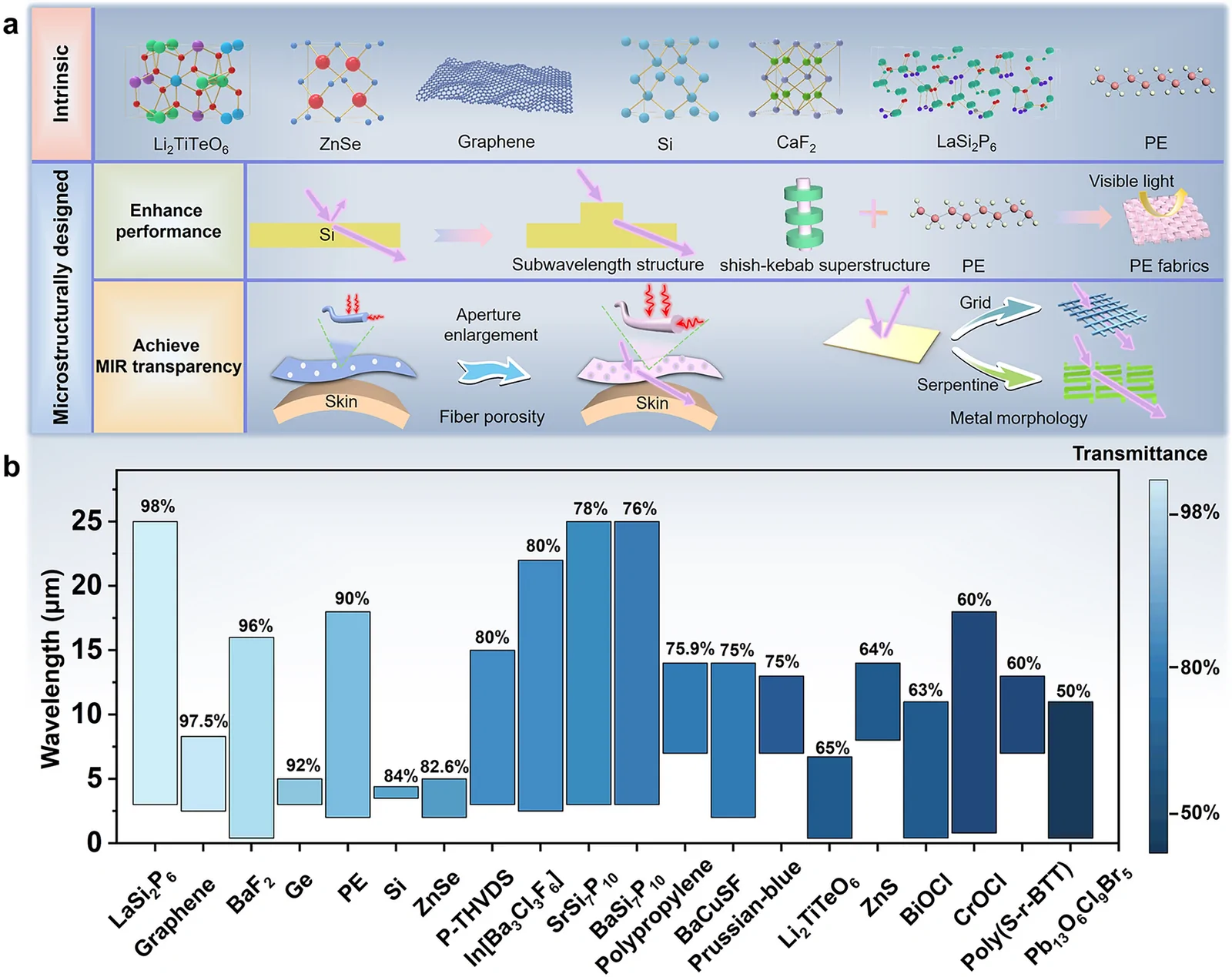
Schematic overview of the classification and spectral transmittance characteristics of high-performance MIR transparent materials.** a** Comprehensive categorization of high *T*_MIR_ materials based on structural similarities. **b** Comparative analysis of MIR spectral range and transmittance

To establish a coherent framework for evaluating and comparing MIR transparent materials, high *T*_MIR_ candidates are classified into two principal categories: intrinsically transparent materials and those whose transparency is enhanced through microstructural design. The former group comprises systems that naturally exhibit low absorption and scattering across the 2.5–20 μm range owing to their wide bandgaps, low phonon activity, and minimal free carrier densities. These materials include oxides, Group VIA compounds, elemental semiconductors, fluorochemicals, and selected polymers or organic substances. The latter category comprises materials that, while not intrinsically ideal, are engineered to achieve high transmittance through structural tailoring. This includes strategies such as refractive index modulation, control of surface morphology, and the design of composite structures. Figure 4a schematically illustrates the dual-path strategy by aligning representative examples according to their intrinsic optical properties and corresponding microstructural design principles. For instance, materials such as Li_2_TiTeO_6_, ZnSe, CaF_2_, and LaSi_2_P_6_ exhibit promising intrinsic transparency within subregions of the MIR spectrum, while polyethylene (PE) demonstrates excellent *T*_MIR_ when processed into porous fibers or patterned into subwavelength structures. Beyond material selection, performance enhancements have been achieved via features such as plasmonic electrode engineering [109], hierarchical subwavelength-masked structures [110], shish-kebab assemblies [111], serpentine wire arrangements [112], or molecular vibration suppression [113], with each approach targeting the suppression of interfacial reflections or volumetric scattering across key MIR subbands.

In interpreting and benchmarking the high *T*_MIR_ performance summarized below, it is also important to consider an engineering relevant distinction beyond the mechanism-based taxonomy, namely the integration form factor. Specifically, a material may be implemented as a self-supporting element such as bulk optics or free standing films and membranes, or it may be intrinsically substrate supported as a deposited thin film or coating. Self-supporting crystals and transparent ceramics are commonly used as windows and lenses, whereas polymeric and membrane-type systems can provide flexible free standing films for large area and conformal thermal management surfaces. By contrast, epitaxial or sputtered films are typically substrate bound, and their device-level *T*_MIR_ is strongly governed by interfacial reflection, impedance matching, and multilayer thickness control. Therefore, the free standing or substrate supported nature should be considered alongside intrinsic absorption and scattering when comparing reported transmittance data across material families.

To facilitate a quantitative comparison across this diverse range of materials, available spectral transmittance data were compiled into a unified bar chart (Fig. 4b), in which representative peak transmittance values are plotted against their corresponding MIR wavelength ranges. Because transmittance is dependent on optical path length, the peak values in Fig. 4b are presented with the specimen thickness or measurement form specified in the cited studies, to provide the necessary context for cross-material interpretation. The data reveal a wide range in both spectral coverage and transmittance efficiency. Certain materials, including LaSiP_3_ and LaSi_2_P_6_ (*T*_3-25_ = 98%), CdTe (*T*_1-25_ = 96%), BaF_2_ (*T*_0.4-16_ = 96%; 3-mm-thick window), SnGa_4_S_7_ (*T*_0.4-25_ = 94%), and PE (*T*_2-18_ > 90%; demonstrated for nano-PE-based layers with thicknesses ranging from 12 to 450 μm), demonstrate transmittance values exceeding 90% across broad MIR spectral windows. Polypropylene (*T*_7-14_ = 75.9%; 35.8 μm film) has also been proposed as an alternative polymer cover for radiative cooling applications These materials generally benefit from wide bandgaps, low free carrier densities, and minimized phonon absorption within the target wavelength range. La-based Zintl phases such as LaSi_2_P_6_ combine structural simplicity with vibrational sparsity, enabling record-high *T*_MIR_ values in the critical 3–25 μm range. These high-performance cases often reflect intrinsic material advantages rather than extrinsic structural manipulation, underscoring the role of chemical and bonding characteristics in defining *T*_MIR_ baselines. At the same time, the dataset reveals materials with more moderate transmittance or narrower spectral spans, often limited by phonon activity, defect absorption, or index mismatch-induced scattering. For example, BiOCl (*T*_0.4–11_ > 63%; reported for crystal plates with thicknesses of 52, 119.5, and 175 μm), Pb_13_O_6_Cl_9_Br_5_ (*T*_0.38–14_ > 50%; measured using an 8 × 3 × 1 mm single crystal), and *β*-Sc(IO_3_)_3_ (*T*_2.5–6.25_ = 50%; measured at a thickness of 32 μm), exhibit relatively low *T*_MIR_ despite being formally transparent, largely due to complex vibrational modes or residual mid-gap states. Other compounds, such as Fe-ZnSe (*T*_5-14_ = 63%, 5–14 μm) or K_2_Bi_2_(SeO_3_)_3_F_2_ (*T*_2.5–20_ = 60%), offer broader coverage but require further optimization to reduce intrinsic absorption or scattering. Meanwhile, polymers like poly(thiohexylvinyl disiloxane) (*T*_3-15_ = 80%; 4.5 μm film) and sulfur-based inverse-vulcanized polymers (*T*_7-13_ = 60%) provide useful spectral selectivity for niche applications such as thermal imaging or flexible optics, though their *T*_MIR_ values remain below those of crystalline inorganics. These comparisons reinforce the importance of aligning material selection with spectral requirements and application contexts, as maximizing *T*_MIR_ often requires trade-offs between bandwidth, optical clarity, mechanical processability, and environmental compatibility.

Based on the dataset compiled in Table 1, which summarizes reported *T*_MIR_ performance and the corresponding fabrication route adopted in each cited study together with representative applications, several structure–property–process correlations can be discerned. For clarity and one-to-one traceability, the “fabrication” entry reports the processing route used in the corresponding reference; alternative established routes are introduced where relevant in the following sections. In addition to transmittance level and bandwidth, the dataset in Table 1 highlights that the practical competitiveness of MIR transparent materials is frequently determined by application facing constraints that differ across material classes. Dense inorganic crystals and transparent ceramics typically offer favorable long-term durability, humidity tolerance, and ultraviolet stability, but their brittleness and limited damage tolerance can impose penalties in mechanical resistance and impact reliability, particularly for large area or lightweight components. Chalcogenide- and halide-based materials can provide attractive MIR windows, yet their practical use may be restricted by chemical and surface stability under humid environments, together with material specific toxicity and handling constraints, which often necessitate encapsulation and stricter deployment protocols. Polymeric films and porous polymer architectures enable scalable, lightweight, and conformal implementations with good mechanical compliance, although their outdoor reliability can be limited by ultraviolet induced aging, abrasion sensitivity, and time-dependent deformation, which can lead to performance drift unless protective layers and lifetime engineering are employed. Accordingly, the comparative trends summarized in Table 1 should be interpreted as coupled optical and reliability descriptors, in which durability, mechanical resistance, ultraviolet stability, humidity tolerance, and environmental compatibility jointly define realistic application access. Materials that exhibit transmittance exceeding 90% across broad MIR ranges, including LaSiP_3_, LaSi_2_P_6_, BaF_2_, CdTe, and SnGa_4_S_7_, generally feature wide electronic bandgaps, low phonon densities, and highly ordered crystalline structures. These systems often crystallize in symmetric, rigid structures that suppress infrared-active vibrational modes and free carrier generation, resulting in minimized absorption over the 2.5–25 μm window. Notably, many of them are accessible via conventional high-temperature solid-state or flux growth routes, underscoring the reliability of thermodynamic synthesis methods in delivering defect-minimized, phase-pure bulk crystals with reproducible *T*_MIR_ performance. In practical implementation, related compositions can also be produced through alternative established routes, such as melt-based crystal growth, chemical vapor transport, and densification- or deposition-based processing when transparent ceramics or thin-film formats are desired, with the preferred route largely dictated by target geometry, device integration demands, and allowable defect and scattering budgets. In contrast, several oxide-based candidates such as (Er, Yb)_2_O_3_ or Li_2_SnTeO_6_, while structurally stable and synthetically tractable, exhibit reduced *T*_MIR_ (65%–80%) or narrower usable windows, primarily due to multiphonon absorption and mid-gap sublevels associated with complex lattice polarizability.

**Table 1:** A representative overview of high *T*_MIR_ materials, including reported transmittance, MIR bandwidth, representative applications, and the processing route used in the cited studies

Classification	Material	Fabrication	*T*_MIR_ (%)	Spectral bands (μm)	Application	References
Group IVA elemental substances	Graphene	Chemical vapor deposition	97.5	2.5–8.3	IR measurements	Hu et al. [95]
	Si	Anodization	84	3.5–4.4	Optical Modulator	Park et al. [99]
	Ge	Chemical vapor deposition	92_max_	3–5	MIR filter	Im et al. [114]
Group VIA compounds	SnGa_4_S_7_	High-temperature solid-state reaction	94	0.40–25	Infrared laser devices	Ensley et al. [115]
	ZnS	Hot pressing	64	8–14	Radiative cooling cover	Bosi et al. [116]
	Fe-ZnSe	Chemical vapor deposition	> 63	5–14	Transparent ceramics	Luo et al. [117]
	ZnSe	Nanosphere lithography	82.6_max_	2–5	Optoelectronic devices and high-power lasers	Huanget al. [118]
	CdTe	Sputtering deposition	96	1–25	MIR microphotonics	Long et al. [119]
Oxides	(K, Na)NbO_3_	Top-seeded solution growth	> 70	0.37–3.8	MIR lasers	Yin et al. [120]
	Sr_1-*x*_Ba_*x*_VO_3_	Pulsed laser epitaxy	75	2–10	Optoelectronic devices	Ha et al. [121]
	*β*-Sc(IO_3_)_3_	Hydrothermal	50	2.5–6.25	NLO optical devices	Wu et al. [122]
	Li_2_TiTeO_6_	Top-seeded solution growth	65	0.38–6.72	Military communication	Du et al. [123]
	Li_2_SnTeO_6_	Top-seeded solution growth	80	0.6–6	Laser lithography	Du et al. [123]
	Rb_4_Li_2_TiOGe_4_O_12_	Solid-state reaction	> 75	0.28–5.58	MIR laser beams	Xia et al. [124]
	(Er_1-*x*_Yb_*x*_)_2_O_3_	Vacuum sintering	80	0.7–9.5	Optical temperature sensor	Feng et al. [125]
Fluorochemicals	BaF_2_	Solid-state reaction	96	0.4–16	All-optical communications	Ensley et al. [126]
	BaCuSF	Multicomponent sputtering	> 75	2–14	Flexible electronics and photovoltaics	Frantz et al. [127]
	K_2_Bi_2_(SeO_3_)_3_F_2_	Mild hydrothermal	> 60	2.5–20	Laser communication	Shi et al. [128]
	In[Ba_3_Cl_3_F_6_]	Hydrothermal	> 80	2.5–22	Molecular sieves	Jiang et al. [129]
	CeF_2_(IO_3_)_2_	Fluorinated homovalent substitution	> 70	0.43–6.46	Optical parameter oscillators	Wu et al. [130]
Other inorganic materials	BaSi_7_P_10_	Solid-state reaction metal salt flux	> 76	3–25	Laser telecommunication	Zhao et al. [131]
	SrSi_7_P_10_	Solid-state reaction metal salt flux	78	3–25	Laser telecommunication	Zhao et al. [131]
	LaSiP_3_	Metal salt flux	98	3–25	Infrared lasers	Sun et al. [132]
	LaSi_2_P_6_	Metal salt flux	98	3–25	Infrared lasers	Sun et al. [132]
	CrOCl	Chemical vapor-phase transport	> 60	0.8–18	MIR pulsed lasers	Wang et al. [133]
	BiOCl	Chemical vapor-phase transport	> 63	0.4–11	MIR pulsed lasers	Ma et al. [134]
	Pb_13_O_6_Cl_9_Br_5_	Flux method	> 50	0.38–14	Wavelength-tunable laser systems	Chen et al. [135]
	Prussian blue	Double salt mixing method	> 75	7–13	Radiative cooling	Cai et al. [136]
Polymers	PE	Vapor-phase polymerization method	> 90	2–18	Personal thermal management	Hsu et al. [137]
	Poly(S-r-BTT)	Inverse vulcanization	60	7–13	Infrared thermal imaging	Jang et al. [55]
	Polypropylene	Commercial thin film	75.9	7–14	Wind shield for radiative cooling	Martorell et al. [138]
	Poly thiohexyl vinyl disiloxane	Sulfur vapor chemical deposition	80	3–15	Optical devices	Peng et al. [139]

Another evident trend lies in the influence of processing techniques on optical performance, especially for materials with polymeric or microstructured architectures. PE-based systems, for instance, exhibit *T*_MIR_ exceeding 90% in the 2–18 μm range, largely due to low molecular vibration absorption and well-controlled polymer chain arrangement. This effect can be further enhanced when combined with controlled chain organization enabled by diverse film-forming routes (*e.g.*, vapor-phase polymerization or melt-based processing) and/or subwavelength structural patterning, which suppress scattering losses and reduce effective refractive index mismatch. In comparison, sulfur-rich polymers and organosilicon derivatives like poly (thiohexylvinyl disiloxane) demonstrate moderate *T*_MIR_ (60%–80%) and spectral coverage up to 15 μm, making them suitable for targeted applications but less ideal for broadband transparency. From an application perspective, materials that provide extended coverage beyond 14 μm, such as K_2_Bi_2_(SeO_3_)_3_F_2_ or SrSiP_10_, are especially valuable for passive thermal regulation and broadband infrared optics. In contrast, those with narrower and more sharply defined transmission windows, like Si or *β*-Sc(IO_3_)_3_, are better suited for wavelength-selective sensing or MIR filtering. Overall, the Table 1 compilation reveals that achieving high *T*_MIR_ requires not only favorable intrinsic electronic and vibrational properties, but also judicious control over fabrication, morphology, and defect chemistry, highlighting the importance of integrated material–process–device design in advancing MIR transparent technologies.

### Intrinsic High *TMIR* Materials

To render the materials overview more analytical, we interpret *T*_MIR_ transparency using a loss channel framework that links bonding and lattice dynamics, composition, and microstructure to the dominant MIR attenuation mechanisms. Bonding configuration and lattice vibrations set the intrinsic phonon limited baseline by governing the vibrational spectrum and multiphonon absorption, thereby bounding the achievable MIR transparency window. Composition then modulates electronic loss by shaping the band structure and defect chemistry, which can introduce absorption from mid-gap states and free carrier Drude attenuation even when phonon absorption is weak. Finally, microstructure determines extrinsic penalties, including scattering, parasitic absorption, and reflection associated with interfaces, through porosity, grain boundaries, secondary phases, surface roughness, and interfacial cleanliness. This hierarchy provides a consistent basis for comparing the six material classes discussed below and for identifying the key levers used to suppress each loss pathway.

#### Oxides

Oxide materials, owing to their structural diversity and chemical tunability, represent a broad class of candidates for achieving high *T*_MIR_. From a crystallographic perspective, the MIR optical properties of oxides are fundamentally governed by factors such as lattice symmetry, phonon mode activity, coordination environments, and the mass and electronegativity of constituent elements [140]. High *T*_MIR_ performance is typically associated with materials that possess large bandgap energies, low phonon absorption cross sections, and minimal free carrier concentrations. These characteristics are often found in oxides with highly symmetric crystal fields, light atomic frameworks, and strong covalent or ionic bonding. For instance, in perovskite-type structures or layered frameworks, the degree of polyhedral connectivity (*e.g.*, corner-sharing octahedra) and the presence of nonpolar or weakly polar bonds significantly influence phonon dispersion and, hence, vibrational absorption [141]. Materials with low-energy vibrational modes or reduced multiphonon processes exhibit diminished attenuation in the MIR region. Furthermore, the incorporation of heavy or highly polarizable cations may introduce localized vibrational modes that broaden absorption bands, while lighter cations and more rigid lattice motifs tend to suppress such effects [142]. Although these structural criteria do not universally guarantee high *T*_MIR_, as extrinsic factors such as defects, grain boundaries, and impurity levels also play crucial roles, they nonetheless offer a fundamental framework for understanding and predicting MIR transparency trends in oxides from a materials science perspective [143]. Consequently, identifying structure–property correlations at the atomic level is essential for rationalizing the MIR optical behavior of oxide materials and guiding the discovery of new candidates with enhanced transparency across targeted spectral regions [31, 144].

**Fig. 5:**
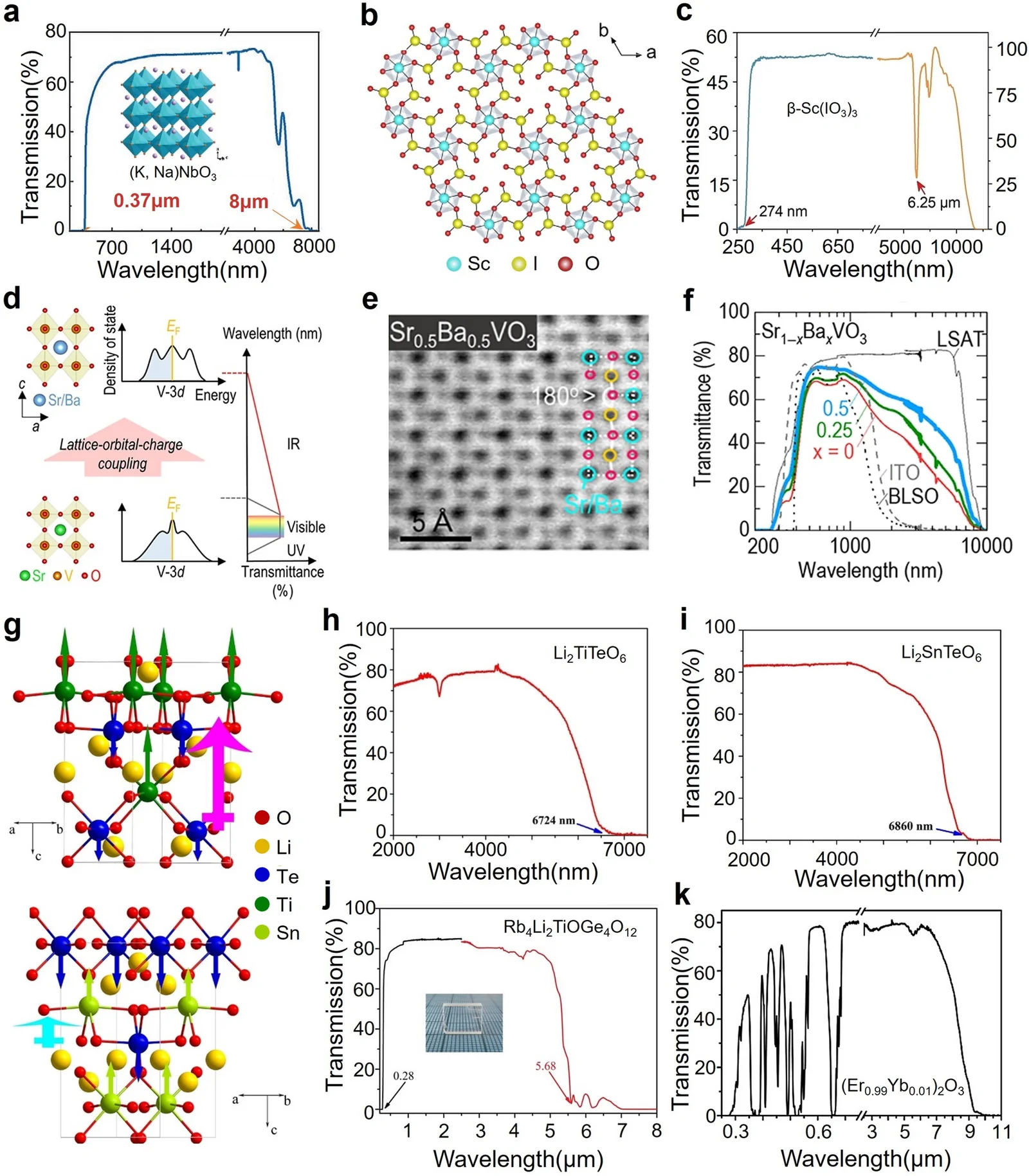
High *T*_MIR_ materials of Oxides. **a** UV–visible–MIR transmission spectra of potassium sodium niobate [120]. Copyright 2022, John Wiley and Sons. **b** Crystal structure of *β*-Sc(IO_3_)_3_ along the c-axis. **c** UV–visible–NIR (left) transmittance and infrared (right) spectra of *β*-Sc(IO_3_)_3_ [122]. Copyright 2022, John Wiley and Sons. **d** Lattice–orbital–charge coupling mechanism for achieving infrared transmittance while maintaining low electrical resistivity in Sr_1-x_Ba_x_VO_3_-related metals. **e** The annular bright-field scanning transmission electron microscope image of Sr_0.5_Ba_0.5_VO_3_. **f** Infrared transmission spectra of Sr_1-x_Ba_x_VO_3_ [121]. Copyright 2022, American Chemical Society. **g** The dipole moment vectors of Li_2_TiTeO_6_ (top) and Li_2_SnTeO_6_ (bottom). h Infrared transmission spectra of Li_2_TiTeO_6_. **i** Infrared transmission spectra of Li_2_SnTeO_6_ [123]. Copyright 2021, John Wiley and Sons. **j** UV–visible–IR transmission spectra of Rb_4_Li_2_TiOGe_4_O_12_ [124]. Copyright 2019, John Wiley and Sons. **k** Infrared transmission spectra of (Er_0.99_Yb_0.01_)_2_O_3_ [125]. Copyright 2022, American Chemical Society

In the past decade, potassium sodium niobate has attracted considerable research interest within the realm of ferroelectric materials due to its environmentally benign and lead-free nature [145–147]. However, the synthesis of optically transparent potassium sodium niobate single crystals has posed enduring challenges. Addressing this issue, Yin et al. have made a significant advancement by successfully producing transparent potassium sodium niobate single crystals using the top-seeded solution growth method [120]. The crystal structure of potassium sodium niobate is a prototypical perovskite, consisting of a 3D lattice formed by niobium (Nb) and potassium/sodium (K^+^/Na^+^) ions. This structure features two distinct oxygen coordination environments. Specifically, niobium ions are coordinated by six oxygen ions to form NbO_6_ octahedra. These NbO_6_ polyhedra are interconnected through shared oxygen atoms, ensuring a uniform distribution of K^+^/Na^+^ ions to maintain charge balance. Additionally, infrared transmission spectra analysis of potassium sodium niobate crystals revealed that *T*_MIR_ exceeds 70% over a broad spectral range of 0.37–8 µm (Fig. 5a). Notably, the ultraviolet short-wave cutoff is observed at 370 nm, and the infrared absorption edge extends to 8000 nm. This finding highlights the significant potential of potassium sodium niobate for MIR applications. Similarly, recent research by Wu et al. has shown that the introduction of additives during reactions plays a pivotal role in forming polar non-centrosymmetric solids [122]. They provide initial evidence that phase transitions induced by these additives can lead to the formation of a novel polar and metastable non-centrosymmetric phase, *β*-Sc(IO_3_)_3_, rather than the stable centrosymmetric phase, *α*-Sc(IO_3_)_3_. Unlike *α*-Sc(IO_3_)_3_, *β*-Sc(IO_3_)_3_ exhibits a three-dimensional [Sc(IO_3_)_3_]_∞_ framework structure with trans-coordinated [IO_3_] groups, aligning nearly parallel to the c-axis of the unit cell, thus manifesting the observed polar crystal structure (Fig. 5b). Infrared spectroscopic analysis reveals that *β*-Sc(IO_3_)_3_ crystals demonstrate transparency across the wavelength range of 2.5–6.25 μm (Fig. 5c). Moreover, transmission spectra spanning ultraviolet–visible–near-infrared (UV–visible–NIR) wavelengths of *β*-Sc(IO_3_)_3_ single crystals indicate a broad UV absorption edge at 274 nm, exceeding those previously reported for non-centrosymmetric rare earth metal-based iodates. These findings highlight the potential of the novel metastable phase, *β*-Sc(IO_3_)_3_, in addressing contemporary challenges in commercial infrared nonlinear optical materials, thereby significantly enhancing its optical performance.

Youngkyoung et al. explored the potential of Sr_1−*x*_Ba_*x*_VO_3_ thin films, produced via pulsed laser deposition [121]. The study demonstrated that 26-nm-thick Sr_1−*x*_Ba_*x*_VO_3_ thin films, with *x* varied at values of 0, 0.25, and 0.5, exhibit high *T*_MIR_ and metallic behavior compared to indium-doped In_2_O_3_ and lanthanum-doped BaSnO_3_ counterparts. This improvement primarily results from the distortion of VO_6_ octahedral structures, adjustments in electronic band configurations, and the larger size of Ba^2+^ ions substituting for Sr^2+^, leading to a reorganization of orbital occupancy (Fig. 5d). High-angle annular dark-field scanning transmission electron microscopy images revealed a significant increase in V–O–V bond angles within VO_6_ octahedra of Sr_0.5_Ba_0.5_VO_3_ relative to SrVO_3_, consistent with theoretical predictions (Fig. 5e). Furthermore, Fig. 5f indicates that increasing Ba concentration notably enhances the high *T*_MIR_ of Sr_1−*x*_Ba_*x*_VO_3_ films, simultaneously improving their metallic properties and effectiveness in electromagnetic shielding. Notably, Sr_0.5_Ba_0.5_VO_3_ films achieved approximately 50% high *T*_MIR_, far exceeding the SrVO_3_’s 27%.

Oxides are increasingly recognized for their potential in nonlinear optics across UV–visible–NIR regions. However, traditional oxides often struggle with limited transmission in the MIR spectrum, restricting their practical use in MIR technologies [148, 149]. Recent research aims to extend the MIR cutoff wavelength to improve its applicability. For example, Du et al. synthesized Li_2_TiTeO_6_ and Li_2_SnTeO_6_ single crystals using the top-seeded solution growth technique [123]. Figure 5g shows these crystals with antiparallel polarization directions in their octahedral units, which may partially cancel their dipole moments. Despite this, both crystals exhibit significant nonlinear optical effects. Li_2_TiTeO_6_ has an infrared absorption edge at 6.72 μm with a transmission range from 0.38 to 6.72 μm and an averaged transmittance of 65% (Fig. 5h). It maintains over 80% transmittance between 600 nm and 6 μm. Li_2_SnTeO_6_, with an absorption edge at 6.86 μm, shows up to 80% *T*_MIR_ and minimal absorption in the atmospheric transparency window (Fig. 5i). These results highlight Li_2_TiTeO_6_ and Li_2_SnTeO_6_ as promising materials for enhancing *T*_MIR_, offering new opportunities for the development of nonlinear Opt. Mater.. Similarly, Xia et al. introduced a novel crystal structure, Rb_4_Li_2_TiOGe_4_O_12_ [124], characterized by compressed TiO_5_ square pyramids and distorted GeO_4_ tetrahedra interspersed with Rb^+^ and Li^+^ cations. Transmission spectra of a 2.75 mm-thick sample, spanning 0.2–8 μm, reveal an infrared absorption onset around 5.58 μm and an ultraviolet absorption onset at 0.28 μm (Fig. 5j). The sharp UV absorption edge at 0.28 μm reflects a wide electronic bandgap, which is essential for minimizing parasitic absorption and ensuring high optical transparency across the visible and near-infrared regions. Rb_4_Li_2_TiOGe_4_O_12_ exhibits a transmittance exceeding 75% in the 1 to 5 μm range, demonstrating excellent transparency with a notable absorption peak at 4.2 μm attributed to path absorption phenomena. Additionally, Feng et al. employed vacuum sintering technology to produce cubic bicomponent transparent ceramics of (Er_1−*x*_Yb_*x*_)_2_O_3_ with x = 0.005 and 0.01 [125]. Transmission spectra of (Er_0.99_Yb_0.01_)_2_O_3_ ceramics, measured over 250 nm–11 μm (Fig. 5k), demonstrate impressive transparency levels of approximately 80% across both visible and infrared regions. The ceramics exhibit an effective infrared cutoff wavelength of around 9.5 μm, comparable to that of pure Er_2_O_3_ ceramics.

In addition to oxide single crystals and transparent ceramics, the oxide family also includes heavy metal oxide glass platforms for 2–5 μm mid-infrared photonics, where manufacturable waveguiding formats are essential. In representative gallate glasses, relatively high glass transition temperature and hardness support mechanical stability, and the nonlinear refractive index can be about an order of magnitude higher than that of silica [150]. These glasses are fiber drawable and have been demonstrated as long fibers and tapers with mid-infrared transparency, enabling high-power-compatible implementations such as supercontinuum generation. Complementarily, rare earth-doped germinate-based oxide glasses provide composition flexibility and relatively high rare earth solubility together with melt processing and polished bulk transparency, supporting mid-infrared emission and amplification schemes [151]. From a mechanistic perspective, the attainable MIR window in oxides is largely bounded by phonon absorption associated with metal oxygen bonding and lattice symmetry. Compositional tuning and defect control therefore act as primary levers to suppress defect related electronic attenuation and to minimize scattering losses in polycrystalline or glass ceramic forms, improving *T*_MIR_.

#### ***Other Group VIA Compounds***

Group VIA chalcogenides are widely investigated for MIR transmission because heavy chalcogen elements and predominantly covalent bonding lower phonon energies, which shifts multiphonon absorption to longer wavelengths and broadens the usable MIR window. The coordination motifs and lattice symmetry determine the distribution and infrared activity of vibrational modes, thereby setting the phonon absorption baseline. Composition and defect chemistry further regulate electronic attenuation through bandgap characteristics, defect states, and free carrier density, which can introduce subgap absorption and Drude-type losses [152]. Consequently, high *T*_MIR_ in this family is achieved by moderating lattice distortion and bonding polarity, while suppressing defects, phase heterogeneity, and free carriers through stoichiometry control and processing optimization. Together, these structural and compositional factors define the MIR transparency window and the attainable *T*_MIR_ level [153].

**Fig. 6:**
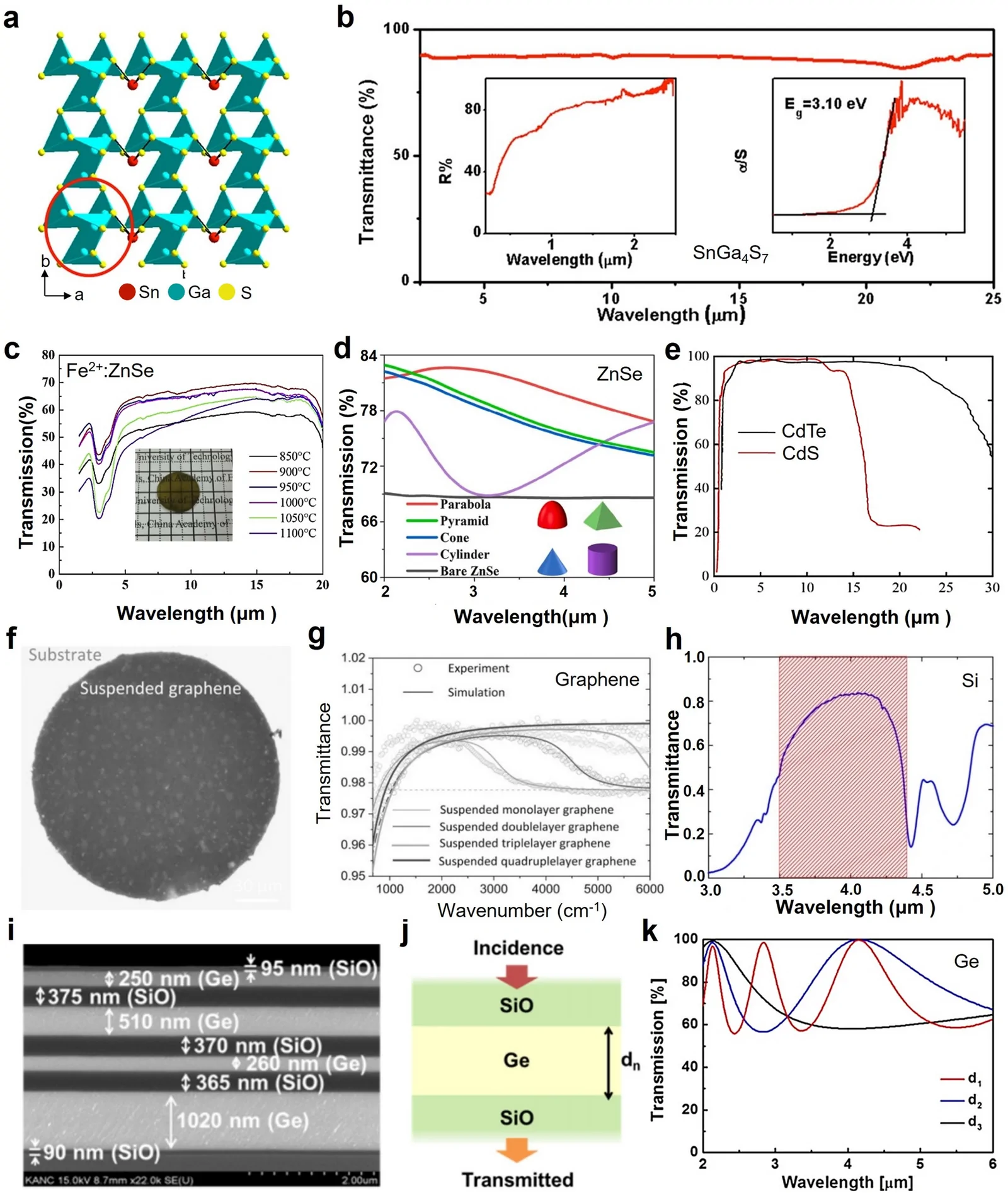
High *T*_MIR_ materials of other Group VIA compounds and Group ⅣA elemental substances.** a** Schematic diagram of the structure of SnGa_4_S_7._ **b** Infrared transmission spectra of SnGa_4_S_7_ [115]. Copyright 2014, American Chemical Society. **c** Infrared transmission spectra of 1 mm-thick Fe^2+^ transparent ceramics sintered at various temperatures, along with a photograph of Fe^2+^ ceramics sintered at 900 °**C** for 2 h under 90 MPa [117]. Copyright 2021, The Optical Society. **d** Transmittance curves of different-profile moth-eye nanostructures [118]. Copyright 2023, Elsevier. **e** Infrared transmission spectra of CdS and CdTe [119]. Copyright 2022, The Optical Society. **f** Optical image of a suspended monolayer graphene on a circular hole (150 µm in diameter) in a SiO_2_/Si substrate. **g** Infrared transmission spectra of large-area suspended graphene with varying layers [95]. Copyright 2017, John Wiley and Sons. **h** Infrared transmission spectra of a mesoporous silicon film with a porosity of 71% and a thickness of 111 μm [99]. Copyright 2016, Springer Nature. **i** Scanning electron microscope image of the filter in the case of d = 530 nm. **j** Structural diagram of the functional cavity. **k** Spectral response of a single cavity [114]. Copyright 2017, The Optical Society

SnGa_4_S_7_ stands out as an outstanding example in group VIA compounds. Luo et al. reported a high-temperature solid-state reaction synthesized SnGa_4_S_7_, which comprises a three-dimensional structure with tetranuclear secondary building units (marked in red circle, Fig. 6a), featuring [Ga_4_S_11_] clusters within the lattice [115]. This compound displays a broad transparent region from 0.40 to 25 μm, with minimal optical absorption peaks within the MIR spectrum, giving broad-spectrum *T*_MIR_ (Fig. 6b). Similarly, transparent ceramics of Fe^2+^:ZnSe, investigated by Luo et al. using hot-pressing techniques, offer insights into the effects of sintering temperature on *T*_MIR_ [117]. It reveals that while samples sintered at 850 °C exhibited reduced transmittance due to residual porosity, those sintered at 900 °C achieved optimal optical quality with transmittance levels reaching approximately 63% at 5 μm and 69% at 14 μm (Fig. 6c). However, further increases in sintering temperature, particularly to 1100 °C, led to a decline in transmittance, indicating the necessity of precise temperature control to maintain high *T*_MIR_ in Fe^2+^:ZnSe ceramics.

In the context of ZnSe, an important MIR material, Huang et al. explored bioinspired moth-eye nanostructures to enhance antireflective properties, as illustrated in Fig. 6d [118]. By employing nanosphere lithography, they fabricated these nanostructures, achieving a significant increase in transmittance up to 82.6% in the 2–5 μm wavelength range. This improvement surpasses the 71.28% transmittance of planar ZnSe, demonstrating the efficacy of nanostructured surfaces in augmenting *T*_MIR_. Additionally, cadmium telluride (CdTe) is recognized for its broad transparent window spanning 1–25 μm. As depicted in Fig. 6e, CdTe maintains a *T*_MIR_ exceeding 80%, outperforming other MIR semiconductors and dielectrics such as Si, Si_3_N_4_, Ge, and As_2_S_3_ [119]. This broad transparency is a testament to CdTe’s superior performance in MIR applications.

Beyond these crystalline II VI semiconductors, chalcogenide glasses represent a foundational platform for MIR technologies because their low phonon energies enable broad mid-infrared transmission, and S-based compositions such as Ga, Sb, S have been reported with an infrared cutoff edge reaching approximately 14 μm together with good thermal stability for glass processing [154, 155]. Moreover, chalcogenide glass platforms are particularly impactful in fiber-based implementations, where high refractive index and nonlinear response support MIR waveguiding and nonlinear photonics, as exemplified by gallium lanthanum chalcogenide glasses that maintain high transparency over 0.5–10 μm and can be structured toward advanced fiber geometries [154]. In these chalcogen-based systems, heavier anions generally lower phonon energies and can mitigate multiphonon absorption, widening the MIR window. At the same time, stoichiometry and impurity control are crucial to limit defect states and free carrier absorption and to avoid scattering from phase separation, which otherwise reduces *T*_MIR_.

#### Group IVA Elemental Substances

Group IVA elemental substances, such as silicon (Si), germanium (Ge), and carbon-based materials including graphene and diamond, exhibit distinct structural and electronic characteristics that critically influence their optical behavior in the MIR region. These materials typically adopt high-symmetry crystal structures such as the diamond cubic structure for silicon and germanium, and the *sp*^2^-bonded honeycomb lattice for graphene, which enable well-defined phonon dispersion and relatively low phonon absorption in the MIR range [156]. The absence of permanent dipole moments and the centrosymmetric nature of these lattices limit infrared-active vibrational modes, thereby suppressing strong one-phonon absorption. In particular, the light atomic masses and strong covalent bonding in these structures contribute to high optical phonon frequencies, pushing significant vibrational absorption beyond the MIR window [157]. For instance, Si and Ge exhibit indirect bandgaps and low free carrier absorption when undoped, allowing substantial transmission in specific MIR subbands, especially under high-purity and low-defect conditions. Graphene, although gapless in its pristine form, possesses exceptionally low optical absorption per atomic layer and supports broadband *T*_MIR_ due to its two-dimensional electron gas behavior and lack of interband transitions in the MIR regime. However, factors such as free carrier concentration, doping level, and localized states introduced by structural or chemical defects can strongly modulate the dielectric function and thus impact *T*_MIR_ [158]. While not all Group IVA elements inherently exhibit high *T*_MIR_ across the entire MIR spectrum, their crystallographic simplicity, low density of IR-active phonons, and tunable electronic structure provide a mechanistic basis for understanding and exploiting their transparency in targeted MIR applications, particularly in integrated photonics, sensing, and thermal imaging platforms [159].

As depicted in Fig. 6f, large-scale suspended single- or multilayer graphene was successfully synthesized by Hu et al. [95] using chemical vapor deposition, giving more than 97.5% transmittance from 1 to 6 μm range. Notably, increasing the number of graphene layers shifted the transmittance slope toward higher wave numbers, yet maintained nearly constant ultra-high *T*_MIR_ (Fig. 6g). This indicates that both doped monolayer and few-layer graphene possess outstanding transmittance properties throughout the MIR spectral range. Si, another Group IVA element, has demonstrated considerable *T*_MIR_, especially in its mesoporous form. Zakar et al. [99] reported on mesoporous silicon having approximately 84% transmission around 4.0 μm with a thickness of 111 μm and a porosity of 71% (Fig. 6h). Ge, recognized for its high transmittance, is highly valued in optical applications, particularly in MIR filters. Ren et al. designed a novel MIR optical filter featuring a wide angular response, achieved through the sequential integration of hybrid subwavelength resonator units (Fig. 6i) [114]. These resonators, using Ge and silicon dioxide as high-refractive-index layers deposited on a silicon substrate, achieved single peak resonance within the MIR spectral range while minimizing sideband effects. Specifically, a Ge cavity was integrated between two silicon dioxide layers to form a functional cavity (Fig. 6j), showing that the resonances of cavities d_1_ and d_2_ occurred at identical spectral positions despite their different periods, while cavity d_3_ did not resonate in the MIR region (Fig. 6k). This phenomenon arises from the fact that both d_1_ and d_2_ possess optical thicknesses that satisfy the resonant condition for constructive interference at specific MIR wavelengths, whereas d_3_ falls outside the effective cavity length required to support such modes. Background transmittance reached approximately 60%, but incorporating functional cavities effectively mitigated high background transmissions, enhancing the sidelobe suppression capabilities of the filter in the MIR band. These specific examples highlight the substantial progress achieved with Group IVA elemental materials in realizing high *T*_MIR_ alongside integrated optical functionalities such as filtering. For Group IVA crystals and related two-dimensional membranes, intrinsic phonon absorption is relatively weak across much of the MIR, so practical transparency is often governed by carrier density introduced by impurities, dopants, or interfacial charge transfer. Controlling composition in this sense, together with surface and microstructural quality that limits scattering and reflection, is therefore central to achieving high *T*_MIR_.

#### Fluorochemicals

Fluorochemicals, particularly binary and complex fluorides such as CaF_2_ and BaF_2_, are widely recognized for their exceptional transparency in the MIR region, a property that can be fundamentally attributed to their unique structural and bonding characteristics [160]. From a crystallographic and lattice dynamics perspective, the high *T*_MIR_ of many fluorides stems from their highly ionic bonds, low atomic masses, and simple cubic or orthorhombic crystal structures, which collectively suppress vibrational absorption within the MIR window. The strong electrostatic attraction between metal cations and fluoride anions results in stiff lattice frameworks with minimal anharmonicity, thereby limiting the generation of low-frequency phonon modes that could otherwise contribute to multiphonon absorption [161]. In addition, the low polarizability of the fluoride ion (F^−^) reduces the strength of dipole-induced vibrational transitions, leading to a significant decrease in absorption cross section across the MIR spectrum [162]. These factors contribute to a sharp phonon cutoff typically above 20 μm, leaving the entire 2.5–20 μm range largely transparent. Furthermore, the wide electronic bandgaps (typically > 10 eV) preclude interband transitions in the MIR, further enhancing their transmittance. However, it is important to note that *T*_MIR_ performance is sensitive to extrinsic factors such as purity, defect density, and grain boundary scattering, especially in polycrystalline forms [163]. While not all fluorochemicals inherently possess high *T*_MIR_, the combination of low refractive index, weak phonon absorption, and chemical inertness renders many fluoride compounds structurally ideal for high-performance optical windows, lenses, and coatings in MIR spectroscopic and thermal imaging systems [161, 164]. Notably, fluorinated systems are typically low index in the MIR, often around 1.34–1.45, which eases broadband index matching but limits optical confinement in compact designs. In layered or coated configurations, the refractive index contrast between adjacent media directly sets the Fresnel reflection at each interface, and therefore the effective *T*_MIR_ can be limited by interfacial reflection losses even when bulk absorption is low.

**Fig. 7:**
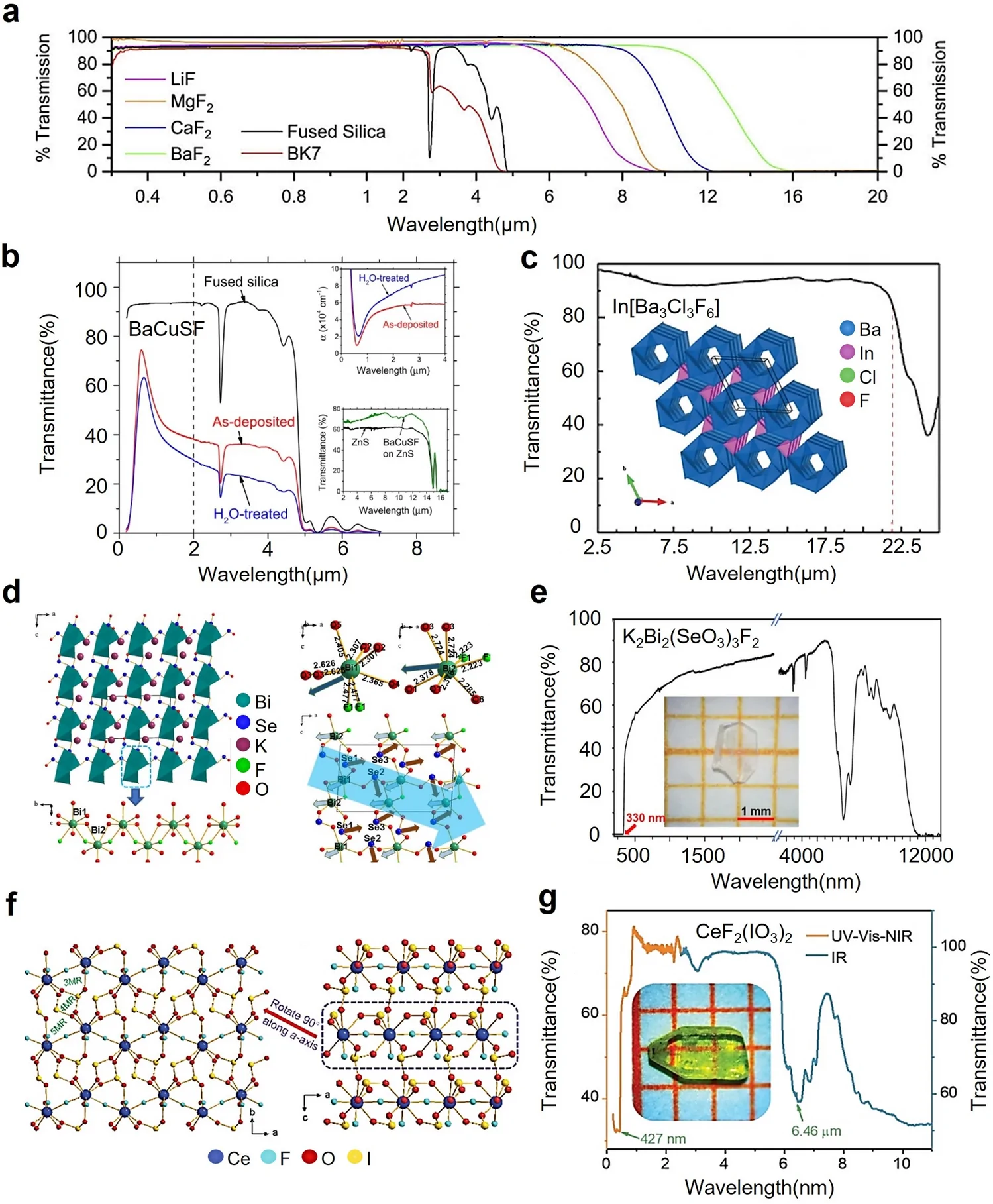
High *T*_MIR_ materials of fluorochemicals. **a** Infrared transmission spectra of fluoride crystals [126]. Copyright 2019, The Optical Society. **b** Transmittance of 150-nm-thick BaCuSF films deposited on and water-treated over a 250 μm-thick fused silica substrate [127]. Copyright 2013, Optical Society of America. **c** Infrared transmission spectra of In[Ba_3_Cl_3_F_6_] [129]. Copyright 2020, Royal Society of Chemistry. **d** Views of the structure of K_2_Bi_2_(SeO_3_)_3_F_2_ along the b-axis. **e** UV–visible–NIR transmission spectra of K_2_Bi_2_(SeO_3_)_3_F_2_ [128]. Copyright 2020, American Chemical Society. **f** Downward view along the c-axis of the 2D layers composed of [CeO_6_F_3_] polyhedra and [IO3] groups, and the final 3D structure of CeF_2_(IO_3_)_2_. **g** UV–visible–NIR transmission spectra of CeF_2_(IO_3_)_2_ [130]. Copyright 2021, Royal Society of Chemistry

The performance of MIR transparent materials can be elucidated through the examination of representative case studies. Trenton et al. conducted an extensive investigation into the optical properties of various fluoride crystals, including BaF_2_, CaF_2_, LiF, and MgF_2_ [126]. Their study utilized Infrared transmission spectra to assess the *T*_MIR_ of these fluorochemical crystals, each with a thickness of 3 mm (Fig. 7a). The results indicate that these fluorescent chemical crystals exhibit a high *T*_MIR_ exceeding 80% up to 6 μm, highlighting their potential for high *T*_MIR_ applications. Further research by Frantz et al. focused on BaCuSF thin films deposited on substrates at 100 °C, suggesting that 150 nm-thick BaCuSF films deposited on ZnS substrates demonstrated an enhanced *T*_MIR_ over ZnS substrate. (Fig. 7b) [127]. These BaCuSF films exhibited characteristics comparable to those of antireflective coatings. Additionally, BaCuSF films on CsI substrates extended their minimum transmittance wavelength to 30 μm, confirming their extensive transparency within the MIR range. Additionally, unlike the strong and broadband vibrational modes of metal–oxygen bonds [165, 166], metal–halide bonds generally demonstrate heightened transparency to infrared radiation [167]. Jiang et al. synthesized a novel molecular sieve, In [Ba_3_Cl_3_F_6_], via hydrothermal methods (Fig. 7c) [129]. This compound, composed of In, Ba, Cl, and F atoms, forms a hexagonal octahedral framework with a high transmittance exceeding 90% in the 2.5–22 μm range, covering critical infrared absorption bands associated with organic molecules. This indicates the compound’s potential for in situ infrared spectroscopic analysis in molecular sieve environments.

Furthermore, Shi et al. investigated alkali metal selenite fluorides incorporating Bi^3+^ ions, discovering a new category of selenite fluoride compounds. They synthesized non-centrosymmetric alkali metal bismuth selenite fluorochemicals, specifically K_2_Bi_2_(SeO_3_)_3_F_2_ (Fig. 7d) [128]. The unit cell of K_2_Bi_2_(SeO_3_)_3_F_2_ includes K, Bi, Se, O, and F atoms, forming a three-dimensional framework. Room temperature UV–visible–NIR transmission spectroscopy further elucidated the unique transmission properties of these crystals, revealing a transmittance exceeding 60% within the 0.5–6 μm range (Fig. 7e). Wu et al. proposed a novel method for synthesizing CeF_2_(IO_3_)_2_, a non-centrosymmetric material, by substituting [IO_3_] anions in Ce(IO_3_)_4_ with fluoride ions (Fig. 7f) [130]. The crystal structure of CeF_2_(IO_3_)_2_, features adjacent [CeF_2_] chains and a three-dimensional framework interconnected by [I(2)O_3_] groups. The transparency range from 0.43 to 6.46 μm, underscores the material’s potential for infrared transmittance applications (Fig. 7g).

These examples illustrate the high transmittance achievable in selected fluorochemicals, underscoring their potential for advanced MIR applications. While these specific fluorochemicals exhibit substantial *T*_MIR_, high *T*_MIR_ is not an intrinsic attribute of all fluorochemical compositions, but rather arises from favorable structure enabled suppression of vibrational absorption and scattering together with appropriate electronic configurations, which highlights the need for targeted material selection and optimization for MIR deployment. In parallel with these crystalline fluorochemical candidates, fluoride glasses provide an enabling fluorine-based platform for mid-infrared photonics, particularly in the 2–4 μm region where low loss waveguiding and rare earth active functionalities are simultaneously required. ZBLAN-type fluoride glasses can be produced by melt processing into bulk components and, critically, drawn into optical fibers, offering a scalable route to MIR waveguides that complements the crystal-based fluorochemical route [168]. Within this platform, rare earth compatibility supports mid-infrared gain media and laser operation, exemplified by Er-doped ZBLAN systems developed for emission around 2.7 μm together with broad infrared transmission. More broadly, practical MIR fiber laser development up to approximately 4 μm has been widely recognized as relying on high-quality fluoride glass fibers, which also underpin nonlinear fiber implementations such as long-wavelength supercontinuum generation [169]. Complementarily, silica-based hollow core fiber architectures employing antiresonant or inhibited coupling guidance can extend practical mid-infrared transmission despite the strong intrinsic absorption of bulk silica beyond about 2.2 μm, enabling fiber-compatible beam delivery and long-path-length gas spectroscopy. In fluorochemical materials, wide band gaps and low carrier densities typically suppress electronic absorption, placing greater emphasis on the phonon absorption baseline set by lattice vibrations. Consequently, defect suppression and high-density processing that reduces porosity and surface roughness are essential to limit scattering and to preserve high *T*_MIR_ in device-scale components.

#### Other High TMIR Inorganic Materials

In addition to the previously mentioned high *T*_MIR_ materials, there are several other inorganic compounds known for their high transparency in this spectral range, such as phosphides, oxyhalides, and Prussian blue [170]. Phosphides, particularly those incorporating rare earth or transition metal cations, represent a structurally diverse class of inorganic materials whose MIR transmittance behavior can be rationalized through their distinct crystallographic frameworks and bonding characteristics [171]. Many phosphides crystallize in highly ordered, covalent–ionic hybrid lattices in which P atoms form tetrahedral or extended polyanionic networks coordinated to electropositive cations. This structural motif often leads to high lattice rigidity and a suppression of low-frequency lattice vibrations, thereby reducing phonon absorption within the MIR region. In particular, compounds such as LaSiP_3_ and LaSi_2_P_6_ exhibit dense three-dimensional frameworks composed of interconnected [SiP_4_] or [Si_2_P_6_] units that help delocalize vibrational modes and minimize dipole-induced transitions, a feature that contributes to their high *T*_MIR_ across broad spectral windows. Furthermore, the relatively low polarizability of phosphorus and the strong covalent nature of P–Si or P–M (M = metal) bonds constrain multiphonon processes and limit free carrier concentrations, thereby minimizing both vibrational and electronic absorption losses [172]. A wide phonon bandgap combined with the lack of mid-gap defect states, particularly in high-purity single crystals prepared through flux growth or solid-state reactions, can significantly improve transparency across the 3 to 25 μm range. However, it is important to recognize that *T*_MIR_ in phosphides is highly sensitive to stoichiometry, crystal phase, and microstructural disorder, particularly in compounds with narrow bandgaps or metallic bonding character [173]. Despite this, the structural regularity, bond strength, and vibrational mode suppression observed in select phosphides offer a compelling basis for their consideration in MIR transparent Opt. Mater., particularly for high-power and thermally stable applications where conventional oxides or halides may be limited [174].

**Fig. 8:**
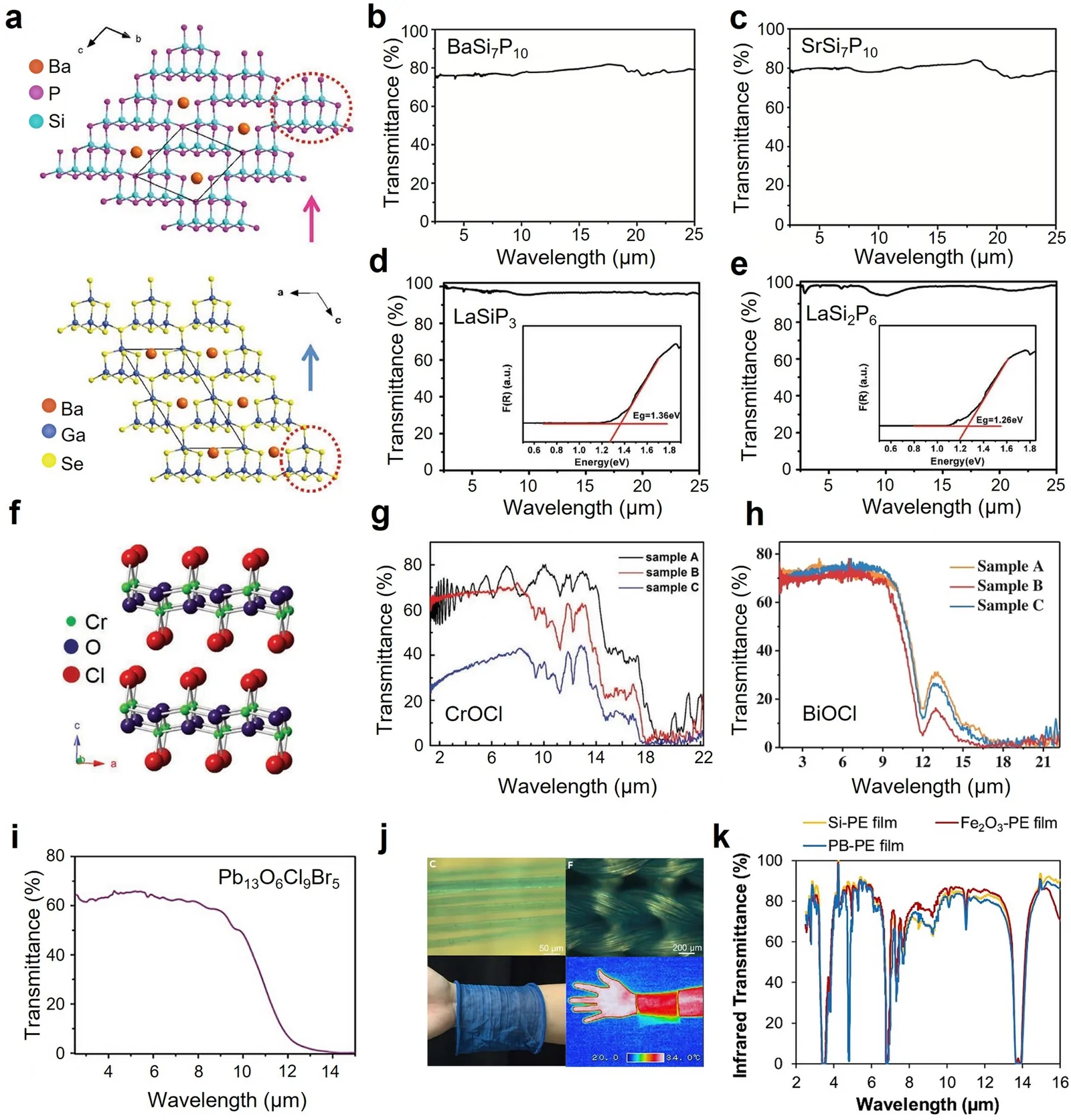
High *T*_MIR_ materials of inorganic substances. **a** Crystal structures of SrSi_7_P_10_ and BaSi_7_P_10_. **b** Infrared transmission spectra of BaSi_7_P_10_. **c** Infrared transmission spectra of SrSi_7_P_10_ [131]. Copyright 2022, John Wiley and Sons. **d** Infrared transmission spectra of LaSiP_3_. **e** Infrared transmission spectra of LaSi_2_P_6_ [132]. Copyright 2021, John Wiley and Sons. **f** Crystal structure of CrOCl. **g** Infrared transmission spectra of CrOCl [133]. Copyright 2020, John Wiley and Sons. **h** Infrared transmission spectra of different BiOCl samples [134]. Copyright 2022, John Wiley and Sons. **i** Infrared transmission spectra of Pb_13_O_6_Cl_9_Br_5_ [135]. Copyright 2020, John Wiley and Sons. **j** Fiber optical microscopy image of Prussian blue PE, photograph of the knitted fabric, and infrared image of the fabric covering human skin. **k** Total infrared transmittance of colored PE textiles and non-colored nanoporous PE textiles [136]. Copyright 2019, Elsevier

Zhao et al. synthesized MSi_7_P_10_ (M = Sr, Ba) materials within the non-centrosymmetric monoclinic space group P1 (No. 1) [131]. Given the structural similarities between SrSi_7_P_10_ and BaSi_7_P_10_, the authors conducted an extensive structural analysis of BaSi_7_P_10_. As shown in Fig. 8a, the BaSi_7_P_10_ structure comprises a three-dimensional framework with tunnellike channels formed by anionic [SiP_4_] tetrahedral clusters. These clusters share corners with Ba^2+^ cations, which occupy the channels to maintain charge neutrality. Both BaSi_7_P_10_ (Fig. 8b) and SrSi_7_P_10_ (Fig. 8c) revealed minimal absorption peaks within the 3–25 μm range, confirming excellent infrared transparency in both materials. Furthermore, Sun et al. successfully synthesized rare earth nickel-based infrared nonlinear optical crystals LaSiP_3_ and LaSi_2_P_6_ using the metal salt flux method [132]. LaSiP_3_ (Pna2_1_) exhibits a 2D structure with alternately stacked SiP_4_ tetrahedral layers and isolated P–P chains, while LaSi_2_P_6_ (Cmc2_1_) features a 3D framework comprising two varieties of SiP_4_ tetrahedral layers and diverse phosphorus polyanions. Both LaSiP_3_ (Fig. 8d) and LaSi_2_P_6_ (Fig. 8e) demonstrate negligible absorption peaks observed within the 3–25 µm range. These findings lay essential empirical groundwork for the potential application of rare earth nickel-based infrared nonlinear optical crystals in the field of infrared optics.

Oxyhalides, characterized by the coexistence of oxide (O^2−^) and halide (typically Cl^−^, Br^−^, or I^−^) anions within a single crystalline framework, exhibit unique structural attributes that critically influence their optical behavior in the MIR region [175]. From a structural standpoint, many oxyhalides crystallize in layered or framework-type lattices, where the mixed anionic environment gives rise to anisotropic bonding and spatially decoupled vibrational modes [176]. The incorporation of halide ions, which possess relatively low electronegativity and high polarizability, effectively reduces the overall lattice phonon energy, thereby minimizing the strength and density of infrared-active vibrational modes within the MIR range. Simultaneously, the presence of rigid oxide sublattices often enhances lattice stability and reduces anharmonic phonon scattering. This dual-anion coordination environment can produce wide phonon bandgaps and dampen multiphonon absorption, enabling partial transparency in the MIR window, especially between 3 and 8 μm. Furthermore, certain oxyhalides, especially those containing heavy main group or rare earth cations, possess stereochemically active lone pairs and exhibit asymmetric coordination geometries, which can further modulate dielectric dispersion and influence the strength of light–matter interactions [177]. For example, [PbO_6_] or [BiO_6_] octahedra coexisting with halide-coordinated polyhedra can introduce localized optical inactivity in specific phonon modes, depending on symmetry selection rules. However, it must be noted that not all oxyhalides inherently exhibit high *T*_MIR_; factors such as halide volatility, phase heterogeneity, or the formation of defect states can introduce significant absorption losses. Nonetheless, the modular structural nature of oxyhalides offers a tunable platform in which vibrational dynamics, bonding strength, and optical phonon activity can be strategically balanced to achieve MIR transparency in select compositions and synthetic configurations.

Wang et al. synthesized CrOCl crystals using chemical vapor transport [133]. The crystal structure comprises CrO bilayers sandwiched between layers of halogen atoms, with these halogen layers stacking along the c-axis of the lattice, revealing significant van der Waals gaps (Fig. 8f). Optical properties of CrOCl crystals were evaluated through room temperature transmission spectroscopy on samples of various thicknesses, covering spectral ranges of 1.28–22.22 μm (Fig. 8g). The results indicated a transparency range spanning 0.8–18 μm, with an IR absorption cutoff observed at 18 μm, slightly extending to 19 μm in the thinnest samples. These findings establish foundational benchmarks and empirical insights essential for further exploration and utilization of the optical characteristics of CrOCl crystals. Additionally, Ma et al. employed the chemical vapor transport method to directly grow sheetlike bulk BiOCl crystals [134]. The crystal lattice of BiOCl consists of alternating layers of [Bi_2_O_2_]^2+^ and double layers of Cl^−^, arranged and stacked along the c-axis direction through van der Waals forces. This unique anisotropic layered architecture promotes the formation of an intrinsic electric field, thereby enhancing the separation of photogenerated electrons and holes and imparting BiOCl with excellent electrical and optical properties, resulting in ~ 70% transmission over the range of 1.28–22.22 μm, with light transmitted along the [001] direction (Fig. 8h). Chen et al. systematically investigated the PbO–PbCl_2_–PbBr_2_ system and identified three lead-based nonlinear optical mixed oxyhalides: Pb_13_O_6_Cl_4_Br_10_, Pb_13_O_6_Cl_7_Br_7_, and Pb_13_O_6_Cl_9_Br_5_ [135]. Pb_13_O_6_Cl_9_Br_5_ has a UV cutoff at 384 nm and an infrared cutoff at 14.0 μm (Fig. 8i). Pb_13_O_6_Cl_4_Br_10_ and Pb_13_O_6_Cl_7_Br_7_ exhibit similar transparency characteristics to Pb_13_O_6_Cl_9_Br_5_. This study demonstrated the broadest reported infrared transparency window (up to 14.0 μm) among all oxide-based nonlinear optical crystals.

Prussian blue and its analogs (PBAs) possess open-framework structures composed of metal–cyanide–metal linkages arranged in cubic or slightly distorted lattices, where the linear–CN– bridges form rigid and periodic three-dimensional networks. This structural regularity suppresses low-frequency lattice vibrations and limits phonon-related absorption in the MIR region. The strong covalency and low mass of the cyanide group shift its characteristic vibrational modes outside the MIR window, thereby minimizing vibrational losses. Additionally, PBAs with low-spin or electronically inert metal centers exhibit reduced free carrier absorption, which can further enhance *T*_MIR_. However, interstitial water, structural vacancies, and intervalence charge transfer may introduce mid-gap states or local absorption bands that degrade transparency. While not all PBAs demonstrate high *T*_MIR_, their crystalline rigidity, phonon suppression mechanisms, and electronic tunability provide a structurally grounded rationale for their potential in MIR transparent applications. As depicted in Fig. 8j, Cai et al. employed Prussian blue as a pigment integrated with polyethylene to produce a flexible polymer substrate [136]. After thoroughly blending these components, the mixture was extruded into fibers suitable for incorporation into woven or knitted textiles, enabling the creation of composite fabrics with enhanced radiative cooling capabilities. Experimental results demonstrate that these colored PE composite textiles achieve up to 80% *T*_MIR_ (Fig. 8k). Furthermore, they exhibit high transmittance in the visible light spectrum, thereby enhancing their effectiveness in passive cooling applications. Across these inorganic frameworks, bonding motifs and lattice connectivity define the vibrational spectrum that sets the phonon absorption baseline, while composition and defect chemistry determine whether additional electronic attenuation emerges. Maintaining phase purity and microstructural uniformity is therefore critical to suppress scattering from inclusions and grain boundaries and to sustain high *T*_MIR_.

#### Polymers and Organics

Polymers exhibiting MIR transparency typically feature simple, symmetric molecular backbones composed of light atoms (*e.g.*, C, H, F), which minimize dipole moment changes during vibrational transitions and thus reduce infrared absorption intensity. In particular, linear polyolefins such as polyethylene (PE) benefit from their highly regular, nonpolar –CH_2_– chains, resulting in discrete and well-separated vibrational modes that avoid overlap with the MIR atmospheric windows [137, 178, 179]. The degree of crystallinity also plays a crucial role: semicrystalline regions facilitate ordered phonon propagation and reduce scattering, while amorphous domains can introduce broad absorption features. Furthermore, the absence of strongly polar functional groups (*e.g.*, carbonyls, hydroxyls) is essential, as such moieties typically exhibit strong fundamental or overtone vibrations within the MIR. In advanced polymer systems, structural design strategies such as increasing chain symmetry, incorporating fluorinated segments to reduce vibrational intensity, or constructing hydrogen-bonded supramolecular networks can effectively modulate phonon activity and improve *T*_MIR_. However, despite these structural advantages, polymers remain susceptible to vibrational overtone absorption and thermal instability at elevated temperatures. Nevertheless, their inherent flexibility, processability, and tunable molecular architecture make polymers an important and versatile class of MIR transparent materials, especially for lightweight, flexible, or conformal optical and thermal devices [180]. In practice, MIR transparent polymers can be fabricated through multiple established processing routes, including melt processing into films, phase separation enabled casting, fiber spinning, and porous or aerogel formation, with route selection primarily governed by the targeted form factor and end-use requirements.

**Fig. 9:**
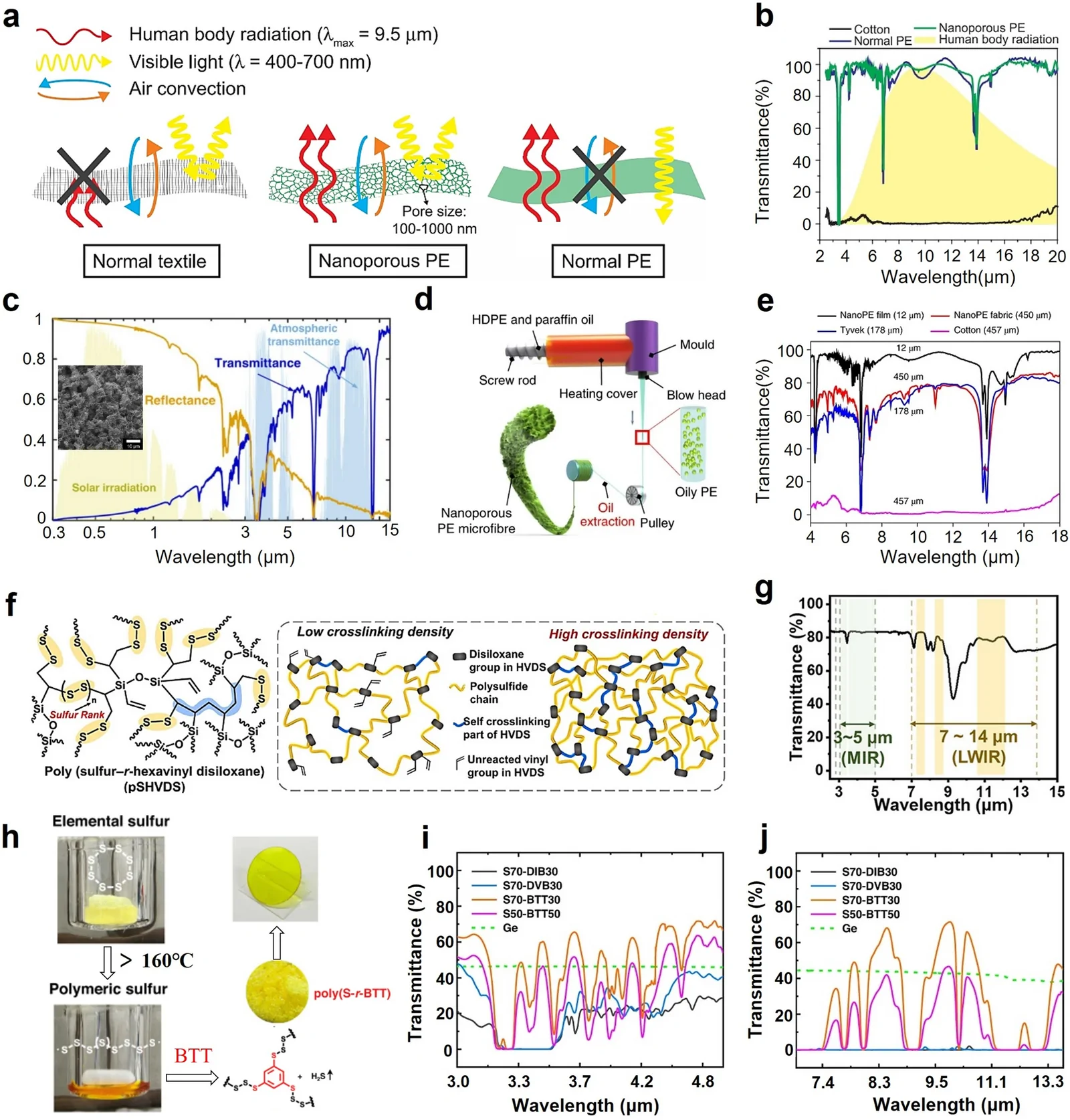
High *T*_MIR_ materials of polymers.** a** Schematic comparison of nanoporous PE, conventional PE, and cotton. **b** Total infrared transmittance of nanoporous PE, conventional PE, and cotton [137]. Copyright 2016, The American Association for the Advancement of Science. **c** Hemispherical transmittance and reflectance of 6 mm-thick PE aerogel samples, along with the standardized AM1.5 solar spectrum and atmospheric transmittance (1976 US standard atmosphere) [32]. Copyright 2019, The American Association for the Advancement of Science. **d** Schematic diagram of the fabrication process for nanofibers. **e** Total infrared transmittance of nanofabrics, nanofilms, Tyvek, and cotton [181]. Copyright 2018, Springer Nature. **f** Schematic diagram of the polythiohexyl vinyl disiloxane network structure and cross-linking. **g** Infrared transmission spectra of polythiohexyl vinyl disiloxane [55]. Copyright 2023, American Chemical Society. **h** Schematic diagrams and actual images of the structures of poly(S-r-BTT), poly(S-r-DVB), and poly(S-r-DIB). **i** TMIR spectra of Ge, S70-DIB30, S70-DVB30, S70-BTT30, and S50-BTT50. **j** Long wave infrared transmission spectra of Ge, S70-DIB30, S70-DVB30, S70-BTT30, and S50-BTT50 [139]. Copyright 2023, Springer Nature

As illustrated in Fig. 9a, this nanostructured porous PE features interconnected pores ranging from 50 to 1000 nm in diameter. These dimensions match the wavelength of visible light, causing strong visible light scattering and making PE appear opaque to the human eye. However, these pores are much smaller than infrared wavelengths, preserving the high infrared transparency of nanostructured porous PE in thin-film form. Leveraging the inherent high *T*_MIR_ of PE, these films exhibit exceptional transparency to IR radiation. The introduced pores give the minimal impact of the nanopores on the overall infrared transmittance of nanostructured porous PE (Fig. 9b). Additionally, Leroy et al. employed a thermally induced phase separation method to synthesize PE aerogels [32]. They subsequently conducted a detailed analysis of the *T*_MIR_ properties of 6-mm-thick PE aerogel samples, comparing them with both the air mass 1.5 solar spectrum and standard atmospheric transmittance. As shown in Fig. 9c, PE aerogels exhibited a transmittance of 79.9% in the wavelength range of 8–13 μm, emphasizing their effective IR penetration capability within this atmospheric transparency window. Additionally, distinct absorption peaks were observed at 3.5, 6.8, and 13.8 μm, arising from the asymmetric stretching, bending, and wagging modes of the CH_2_ molecules inherent in the PE aerogel structure. Innovative approaches in producing nanoscale PE fibers also show promising results. Peng et al. introduced a method for producing nanoscale PE fibers, depicted in Fig. 9d, enabling continuous fiber production with excellent uniformity and mechanical strength [181]. Their comprehensive evaluation of the IR transmittance of these nanoscale PE fabrics, as shown in Fig. 9e, revealed that the films maintained exceptional *T*_MIR_ even at varying thicknesses, with a minimum of 12 μm. Remarkably, even in nanoporous fabrics with a thickness of 450 μm, *T*_MIR_ remains above 70% in the 4–18 μm range, demonstrating the high transmission capability of PE materials in the MIR spectrum.

Sulfur-rich polymers, another promising category, offer significant potential for IR optical applications, especially in the LMIR spectrum due to the inherent properties of sulfur. Wontae et al. introduced a novel sulfur-rich polymer, polythiohexyl vinyl disiloxane, giving impressive *T*_MIR_. As depicted in Fig. 9f, this polymer features a uniform structure with significant cross-linking effects, validating the feasibility of producing cross-linked films using polysulfide and sulfur components. Their study showed that a 4.5-μm-thick film of this polymer achieved over 70% transmittance across the 2.5–15 μm spectral range, with minimal optical losses in specific wavelength bands, as illustrated in Fig. 9g. This performance is attributed to the polymer’s unique composition, including infrared-transmitting S–S bonds, low infrared-absorbing C–S bonds, and segments of hexyl ethylene vinyl disiloxane copolymer. Recent research has increasingly focused on sulfur-containing polymers due to their ability to address inherent optical limitations in traditional polymers. For example, Miyeon et al. synthesized poly(S-r-BTT) copolymers using the trifunctional aromatic thiol cross-linker 1,3,5-trithiane (BTT) to sulfurize elemental sulfur [139]. As shown in Fig. 9h, it highlights the superior *T*_MIR_ of poly(S-r-BTT) copolymers compared to those based on other cross-linkers like 1,3-divinylbenzene (DVB) or diisopropenylbenzene (DIB). The symmetric structure of BTT and the presence of post-sulfurization infrared-active sulfur simplify the infrared spectra of the copolymer, enhancing its transmittance capabilities. Additionally, elemental analysis confirmed the lower organic content in poly(S-r-BTT) copolymers, resulting in reduced infrared absorption. Infrared transmission spectra of different polymers, as shown in Fig. 9i, j, indicated that poly(S-r-BTT) copolymers exhibit simpler fingerprint regions and better *T*_MIR_ performance compared to other sulfur-containing copolymers. In polymers, the intrinsic MIR window is dictated by the vibrational modes of the backbone and side groups, so chemical composition directly determines absorption band positions and intensities. Microstructure, including crystallinity, phase separation, and porosity, then controls scattering and reflection losses, making molecular design and morphology control complementary levers for improving *T*_MIR_. Overall, intrinsic high *T*_MIR_ materials still face coupled trade-offs among the MIR window, defect related electronic attenuation, and scalable manufacturability. Key unresolved challenges include suppressing defects and microstructural heterogeneity at device scale, and separating bulk absorption from scattering and interfacial reflection when comparing reported *T*_MIR_ performance.

### Microstructural Design for Achieving High *TMIR* Materials

To further elucidate the role of structural engineering in modulating MIR optical performance, the following sections examine two major categories of microstructural design strategies: (1) structurally enhanced *T*_MIR_ materials, which involve the optimization of materials already possessing intrinsic MIR transparency through surface texturing, gradient refractive index architectures, or hierarchical ordering; and (2) structurally tailored extrinsic *T*_MIR_ materials, in which structural modification is used to induce or significantly improve MIR transparency in materials that are not inherently transmissive. Surface roughness and porosity introduce refractive index inhomogeneity and boundary irregularities, which enhance MIR scattering and reduce the specular component of transmittance, leading to a lower *T*_MIR_. Grain boundaries and inclusions, including secondary phase particulates, further act as internal scattering centers by providing additional optical contrast and discontinuities, thereby increasing attenuation and degrading *T*_MIR_. For both classes, emphasis is placed on analyzing how specific microstructural features such as subwavelength patterning, porosity, alignment, and morphology influence the interaction between infrared light and matter. By correlating representative experimental studies with underlying optical principles, this discussion highlights the mechanistic basis through which structural design can suppress reflection, minimize scattering, and extend transmission bandwidths across the MIR region [182].

#### Structurally Enhanced TMIR Materials

**Fig. 10:**
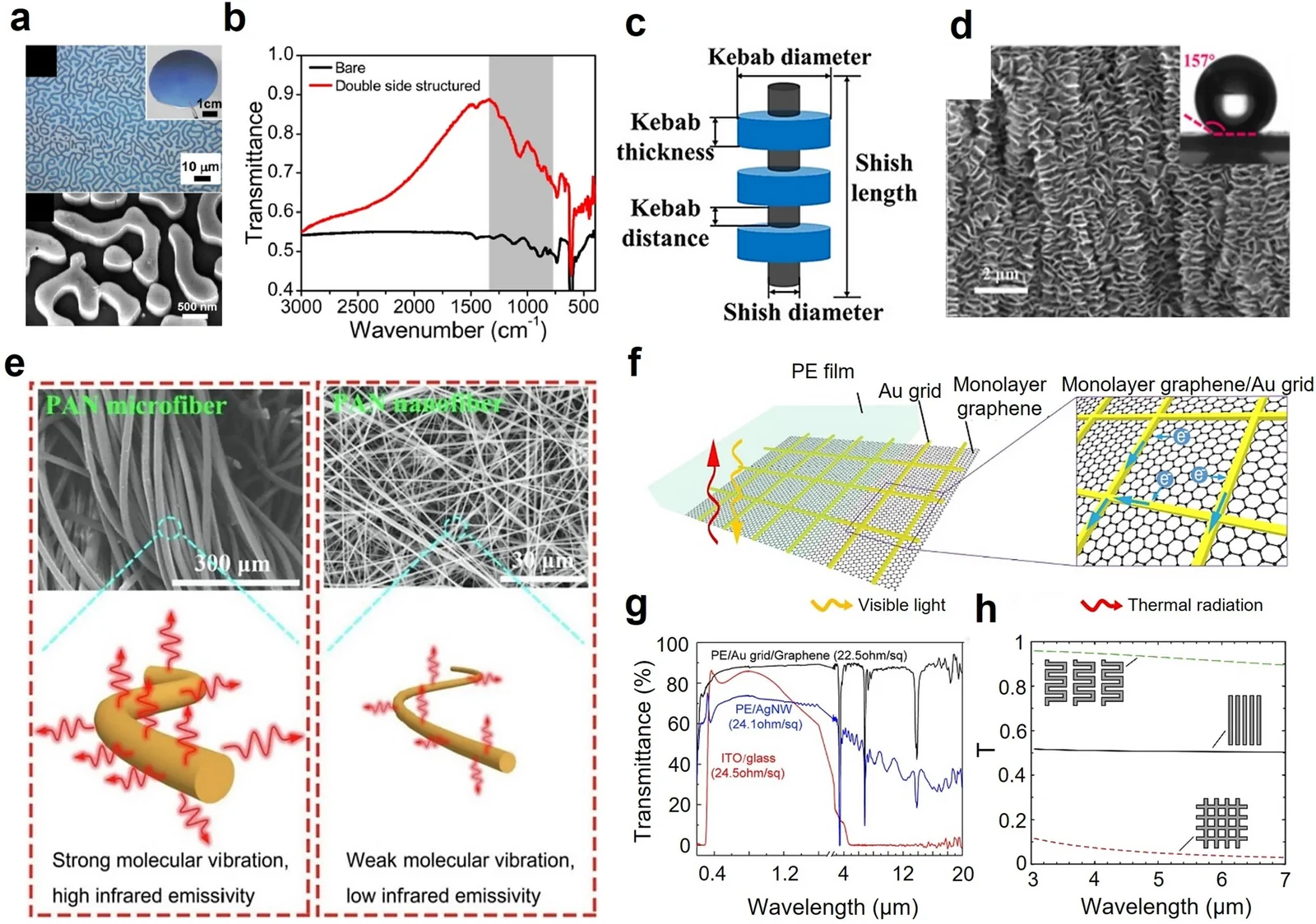
High *T*_MIR_ materials via microstructural tuning. **a** Optical microscopy images of phase-separated polystyrene patterns (top) and oblique scanning electron microscopy images of Si subwavelength structures (bottom). **b** Transmission spectra of double-sided structured Si wafers [110]. Copyright 2018, The Optical Society. **c** Illustration of shish-kebab in polymer science. **d** Scanning electron microscopy images of shish-kebab superstructure film [111]. Copyright 2023, American Chemical Society. **e** Scanning electron microscopy images of polyacrylonitrile microfiber (left) and polyacrylonitrile nanofiber (right) [113]. Copyright 2022, Elsevier. **f** Schematic diagram of the structure of ultra-broadband transparent conductive electrodes. **g** Comparison of transmittance between ultra-broadband transparent conductive electrodes and other types of transparent conductive electrodes [109]. Copyright 2021, American Chemical Society. **h** Transmission spectra of unpolarized light through serpentine wire arrays, straight wire arrays, and grid structures in vacuum [112]. Copyright 2014, The Optical Society

While Si inherently exhibits high transparency in the infrared spectrum, its high refractive index can cause significant reflectance, thereby impeding infrared device performance. To address this issue, subwavelength photonic structures, such as the moth-eye structure, have been employed to reduce the refractive index and enhance transmittance. Zhang et al. introduced an innovative method using phase-separated polystyrene/PMMA blends as masks to fabricate Si subwavelength structures with significantly improved *T*_MIR_ [110]. Figure 10a demonstrates the uniform deposition of polystyrene on silicon wafers, indicating the scalability of this approach for production. The fabricated masks featured smooth sidewalls on the Si structures, and researchers successfully created double-sided Si subwavelength structures through iterative fabrication on polished silicon wafers. IR transmittance evaluations revealed maximum *T*_MIR_ approaching 70% and 89% for single-sided and double-sided structures, respectively (Fig. 10b). These results highlight the potential of Si subwavelength surface structures to significantly reduce the refractive index and enhance *T*_MIR_.

In another example, polyethylene demonstrates inherent high transparency in the MIR spectrum. Nevertheless, its utility in personal thermal management is limited due to its insufficient capacity to reflect incident solar radiation. The shish-kebab structure, a self-assembled architecture, comprises multilayers with linear cores surrounded by layered crystals (Fig. 10c). This structure imparts superhydrophobic properties and enhances solar radiation scattering. Li et al. utilized flow-induced crystallization to integrate polyethylene films with complete kebab-like structures, producing thermal management materials characterized by superhydrophobic properties and effective solar reflectance, termed shish-kebab superstructure films [111]. The micro–nanofeatures confer pronounced superhydrophobic characteristics to the film (Fig. 10d). Studies show that shish-kebab superstructure films transmit a significant portion of infrared radiation emitted by human skin, outperforming conventional textiles such as polyester, cotton, and Tyvek in infrared transmittance across the 8–13 μm wavelength range, achieving an average *T*_MIR_ of 87%. These findings underscore the superior infrared transmittance capabilities of shish-kebab superstructure films in the MIR spectrum, highlighting their potential in personal thermal management applications.

#### Structurally Tailored Extrinsic TMIR Materials

In the MIR spectrum, many fibers exhibit limited transmittance due to inherent high absorption. However, passive radiative heating applications demand fibers with high *T*_MIR_ to allow significant penetration of infrared radiation and reduce emissivity, facilitating efficient heating processes. Adjusting fiber porosity through microstructural engineering has emerged as a promising strategy [183]. For example, Li et al. demonstrated a method to reduce the infrared emissivity of textile fibers by decreasing their diameter, creating polyacrylonitrile nanofibers via electrospinning techniques [113]. As illustrated in Fig. 10e, ultra-fine polyacrylonitrile fiber fabrics exhibited 85% infrared emissivity, enhancing thermal dissipation, while polyacrylonitrile nanofiber textiles showed significantly lower infrared emissivity at only 14%. The reduction in fiber diameter results in a proportional decrease in fiber volume, thereby diminishing the intensity of molecular vibrational modes and lowering infrared emissivity [15]. Therefore, adjusting fiber diameter is a viable approach for fine-tuning the infrared optical properties of fiber materials.

Metals, due to their intrinsic electronic structure, typically exhibit significant absorption and scattering in the MIR spectrum, leading to reduced transmittance. To address the requirements of transparent conductive electrodes, metals need to exhibit high *T*_MIR_ [184]. Microstructural engineering on metal surfaces has been proposed to mitigate optical losses and optimize the production of wideband transparent conductive electrodes, managing both solar and thermal radiation effectively. For instance, Rao et al. proposed a design for wideband transparent conductive electrodes incorporating a monolayer of graphene, a gold microgrid, and a PE film, as depicted in Fig. 10f [109]. This design achieves a *T*_MIR_ of 84.87% across the 0.2 to 20 μm wavelength range, demonstrating excellent optical transparency. Due to the presence of a gold microgrid, only 2% of shadow loss is observed (Fig. 10g). The microgrid consists of a mesh structure with grid widths of 10 μm and a spacing of 1 mm. Integrating a PE film as a flexible, infrared-transmitting substrate further enhances the transparency of these wideband transparent conductive electrodes. Additionally, Clark et al. explored the optical properties of two-dimensional nanoscale metal structures integrated into metamaterial frameworks for infrared applications [112]. They found that optical losses in serpentine structures were less than 7%, primarily due to reduced electrical conductivity from their curved configuration and inductance. The optical performance of serpentine, linear array, and grid structures was compared (Fig. 10h). Serpentine structures showed *T*_MIR_ values between 0.89 and 0.96 for unpolarized light. Linear structures exhibited metallic behavior along the conducting direction and dielectric behavior in the orthogonal direction, resulting in an average *T*_MIR_ of about 0.5. Grid structures demonstrated metallic properties in both directions, with *T*_MIR_ ranging from 0.02 to 0.12. These results highlight the significant transmittance capabilities of serpentine 2D metal structures within the MIR spectrum.

## Applications Enabled by High *TMIR* Materials

### Thermal Management Systems

To comprehensively assess the role of high *T*_MIR_ materials in thermal management, the following sections explore three major application domains: (1) radiative cooling, (2) radiative heating, and (3) dynamic radiation regulation. Each domain utilizes the capacity of materials to transmit or modulate thermal infrared radiation in a controlled fashion [185–187]. These applications exploit different aspects of MIR transparency: in radiative cooling, high *T*_MIR_ in the atmospheric window enables effective heat dissipation into outer space; in radiative heating, *T*_MIR_ facilitates the passage of solar heat or enhances photothermal conversion; and in dynamic regulation, materials with switchable emissivity or phase states allow for adaptive thermal control. Optical anisotropy, birefringence, and texturing can introduce angle and polarization dependence in Fresnel reflectance and scattering, which reduces the device effective MIR transmittance and can also alter the apparent emittance through the coupled balance of transmittance, reflectance, and absorption. Analysis of representative experimental studies reveals the critical roles of intrinsic material characteristics and engineered microstructures in modulating thermal radiation, offering a foundation for energy-efficient strategies under diverse environmental constraints. This application-focused framework provides a mechanistic understanding of how high *T*_MIR_ performance can be harnessed to address pressing challenges in passive and active thermal management systems. In practice, system-level performance is governed not only by *T*_MIR_ magnitude but also by spectral selectivity, angular response, and interfacial losses in multilayer assemblies. Durability under humidity, ultraviolet exposure, abrasion, and thermal cycling remains a practical constraint that can offset intrinsic optical advantages. In practice, system-level performance is governed not only by *T*_MIR_ magnitude but also by spectral selectivity, angular response, and interfacial losses in multilayer assemblies. Durability under humidity, ultraviolet exposure, abrasion, and thermal cycling remains a practical constraint that can offset intrinsic optical advantages [188].

#### Radiative Cooling

Radiative cooling operates on the principle of selectively emitting thermal radiation through the atmospheric transparency window (8–13 μm) into outer space, which acts as an ultra-cold heat sink [189–192]. Materials with high *T*_MIR_ are critical in this process, as they enable the efficient passage of thermal photons from underlying emitters to the external environment without significant spectral attenuation. Specifically, high *T*_MIR_ materials serve as optical interfaces or encapsulating layers that minimize parasitic absorption and reflection in the MIR regime, thereby preserving the radiative flux required for subambient cooling [193–195]. Such encapsulating strategies can also be extended to infrared transparent cover layers, which help reduce non-radiative heat exchange while preserving radiative transmission within the atmospheric window [116]. Their transparency within the atmospheric window ensures that emitted thermal energy is not trapped or reabsorbed, allowing for sustained heat rejection even under solar exposure when paired with reflective or spectrally selective components [196–198]. Thus, the optical compatibility of high *T*_MIR_ materials with the Earth’s infrared emissivity profile directly governs the thermodynamic efficiency and practical viability of radiative cooling systems [199].

**Fig. 11:**
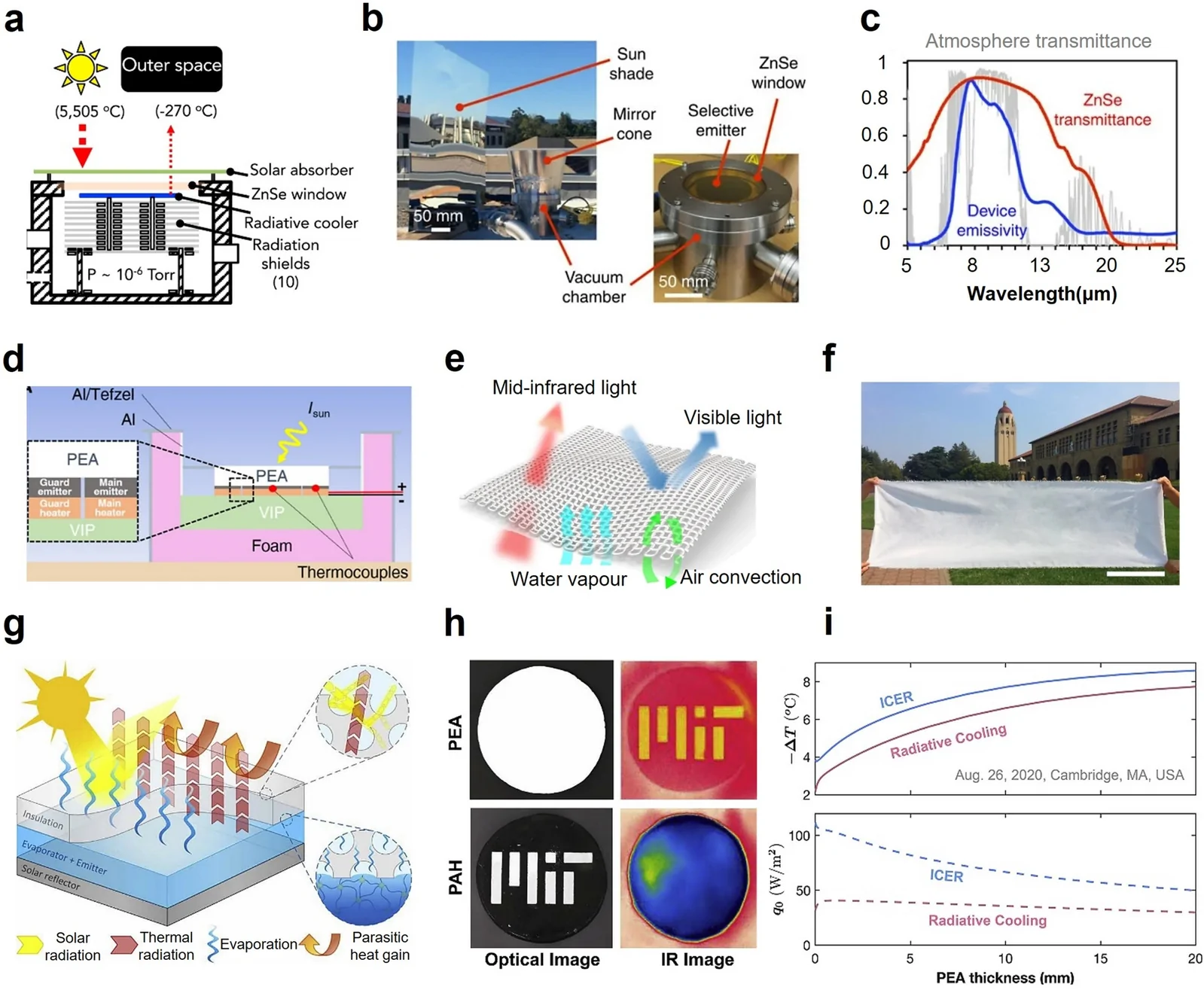
Applications of high *T*_MIR_ materials in radiative cooling. **a** Schematic of the experimental setup [40]. Copyright 2019, Elsevier. **b** Schematic diagram of the field test setup and detailed images of the vacuum chamber. **c** Emissivity of selective emitters and the transmittance of ZnSe windows [33]. Copyright 2016, Springer Nature. **d** Schematic diagram of the radiative cooler principle [32]. Copyright 2019, The American Association for the Advancement of Science. **e** Schematic diagram of nano-PE fabric. **f** A photograph of a large nanofabric [181]. Copyright 2018, Springer Nature. **g** Schematic diagram of the structure of the evaporative and radiative insulation cooling device. **h** Optical and infrared images of polyacrylamide hydrogels and PE aerogels. **i** Prediction of different PE aerogels thicknesses using the experimental device model based on the stagnation temperature drop -ΔT and environmental temperature cooling power q_0_ [204]. Copyright 2022, Elsevier

Within this context, ZnSe, as an infrared emission window material, demonstrates the potential in achieving effective radiative cooling in specific setups. Chen et al. introduced an experimental apparatus that combines a radiative cooler and a solar absorber (Fig. 11a) [40]. To minimize parasitic heat losses, the radiative cooler is enclosed in a vacuum chamber with a ZnSe window, known for its exceptional transparency in the 8–13 μm wavelength range. This setup facilitates efficient thermal radiation transmission, allowing the solar absorber to achieve the desired cooling effect. Under peak sunlight conditions, the experimental results at Stanford University show a temperature increase of the solar absorber by over 24.4 °C and a decrease of the radiative cooler temperature by more than 28.9 °C compared to the ambient temperature. Further enhancing radiative cooling, Zhu et al. developed a device featuring a selective emitter within a vacuum chamber, shielding it from direct solar radiation (Fig. 11b) [33]. The device integrates a selective thermal emitter with a transparent atmospheric window, reducing parasitic thermal losses. Detailed analyses of the emitter’s emissivity and the ZnSe window’s transmittance characteristics reveal exceptional *T*_MIR_, facilitating effective thermal exchange between the emitter, atmosphere, and outer space (Fig. 11c). Experimental data indicate a maximal temperature reduction of about 42 °C when the apparatus was exposed to peak solar irradiance, while the selective emitter itself was shielded from direct sunlight and operated primarily under diffuse illumination, demonstrating the strong cooling potential of ZnSe window-based vacuum systems when the ambient temperature is below 60 °C.

Recent advancements include integrating PE with emitters to enhance radiative cooling efficiency. Leroy et al. introduced a composite material comprising PE aerogel, as depicted in Fig. 11d, combined with existing emitters [32]. In this representative design, PE aerogel functions as an infrared transparent insulating layer that suppresses non-radiative heat gain while maintaining radiative transmission within the atmospheric window, and optical characterization showed that a 6 mm PE aerogel achieved a *T*_MIR_ of 79.9% across 8–13 μm. More recently, Yang et al. reported a polyethylene nanoflake aerogel cooling skin that provides an updated benchmark by reaching about 97.9% porosity together with an average solar reflectance of about 0.96 and an average mid-infrared transmittance of about 0.80 at millimeter-scale thickness [200, 201]. Comparative analysis reveals that incorporating PE aerogel significantly lowers emitter temperatures, confirming its suitability for advanced cooling applications. PE-derived microfibers also hold promise for developing high-performance nanofabrics. Peng et al. demonstrated large-scale extrusion of uniform, nanostructured porous PE microfibers, producing nano-PE fabrics with high *T*_MIR_ and visible light opacity (Fig. 11e) [181]. These fabrics exhibit enhanced abrasion resistance and can be rapidly manufactured (Fig. 11f). Compared to commercial cotton fabrics, nano-PE fabrics offer superior cooling capabilities, reducing skin temperature by 2.3 °C and achieving over 20% energy savings in indoor cooling applications. More recently, MIR transparent wearable platforms have been advanced toward practical deployment through scalable fabrication of transmission-type daytime radiative cooling layers that simultaneously enhance solar reflectance and mid-infrared transparency, and through woven polyethylene textiles produced using standard textile manufacturing approaches with engineered moisture transport to improve thermal comfort, durability, and sustainability [202, 203]. Additionally, Lu et al. proposed a novel cooling system, incorporating a solar reflector, a dual-function evaporative layer, and a breathable, infrared-transmissive insulation layer (Fig. 11g) [204]. Using commercial materials such as 3 M enhanced mirror film, polyacrylamide hydrogel, and PE aerogel, the system achieves a solar reflectance of 92.2%, *T*_MIR_ of 79.9%, and thermal conductivity of 28 mW m^−1^ K^−1^ (Fig. 11h). Experimental studies and a predictive model show that increasing PE aerogel thickness significantly reduces the stagnation temperature, enhancing the cooling system’s efficiency (Fig. 11i).

#### Radiative Heating

Radiative heating in MIR thermal management commonly relies on enabling efficient coupling of long-wave thermal radiation through a protective layer while suppressing non-radiative heat losses. In this context, high *T*_MIR_ materials function as infrared transparent windows or encapsulants that transmit MIR radiation with minimal attenuation, thereby permitting radiative exchange across an interface while providing mechanical protection and reducing convective heat loss [205–208]. In practical composites, additional layers may be introduced to tailor spectral selectivity or surface emissivity; however, the central material requirement discussed here is the high MIR transmittance of the infrared transparent component that governs the radiative throughput.

**Fig. 12:**
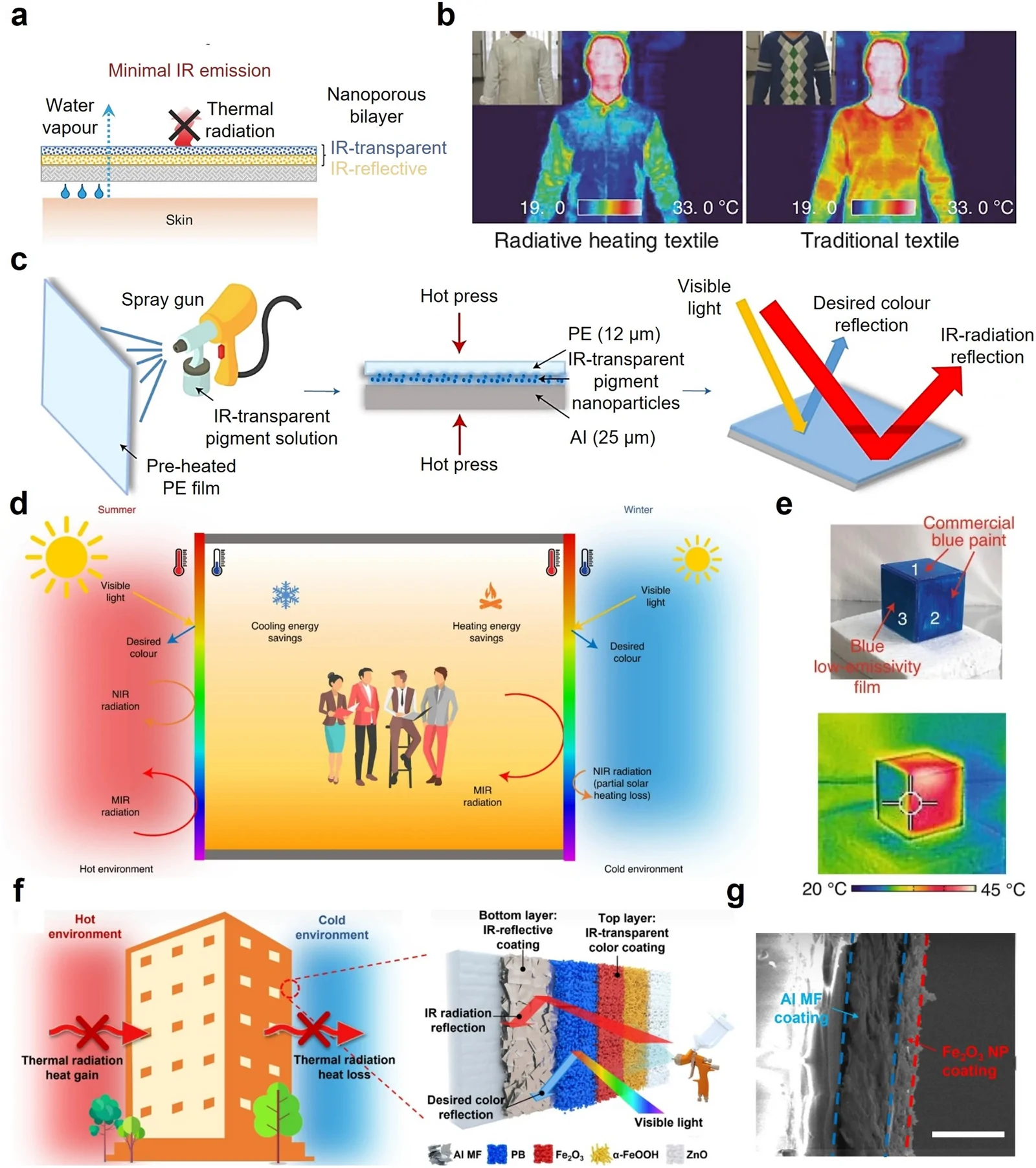
Applications of high *T*_MIR_ materials in radiative heating. **a** Schematic diagrams of nanophotonic structures comprising an infrared transparent layer and an infrared-reflective layer in heated textiles. **b** Thermal imaging of garments made from radiation-heated cotton/silver/PE textiles and traditional textiles worn by humans [37]. Copyright 2017, Springer Nature. **c** Schematic diagram of the fabrication process for colored low-emissivity films. **d** Simulation of room temperature thermal radiation using the law of Planck at 300 K. **e** Optical photograph of the building model (top) and thermal image of the building model under infrared light (bottom) [209]. Copyright 2022, Springer Nature. **f** Schematic illustration of the working mechanism of colored low-radiation coatings (left) and the schematic diagram of the painted bilayer structure (right). **g** Scanning electron microscopy image of the cross section of a bilayer red low-emissivity coating [210]. Copyright 2023, PNAS

Typical polymers such as polyethylene (PE) exhibit high *T*_MIR_, which is advantageous for heating-related configurations that require MIR transparent encapsulation with low parasitic absorption. For instance, Cai et al. developed a dual-layer nanophotonic textile consisting of an infrared transparent nano-PE layer and an additional functional layer, both incorporating nanoporous features (Fig. 12a) [37]. In this architecture, the nano-PE layer provides the MIR transparent pathway that preserves mid-infrared transmission through the textile while maintaining a lightweight and wearable form factor. The reported thermal imaging results (Fig. 12b) are consistent with reduced apparent infrared emission from the outer surface, indicating suppressed radiative heat loss under indoor conditions. These observations highlight that PE-based layers can serve as scalable MIR transparent encapsulants for wearable systems, where maintaining high *T*_MIR_ and low absorption is essential for radiative coupling through a protective cover. Peng et al. reported a spectrally selective bilayer film composed of a pigment–PE composite layer on an aluminum layer (Fig. 12c) [209]. In this design, the pigment–PE composite provides a processable polymer layer that maintains substantial mid-infrared transparency while enabling visible coloration (Fig. 12d), supporting large-area building envelope implementations where radiative exchange is mediated through a coated interface. Device-level demonstrations using a cubic building model (Fig. 12e) further illustrate that layered architectures can modulate radiative interaction with the surrounding infrared environment. Within the present discussion, the key materials implication is that PE-based composite films can retain high *T*_MIR_ in scalable formats, thereby enabling MIR radiative throughput through mechanically robust coatings and encapsulation layers.

Fe_2_O_3_ is also widely used in architectural coatings and can function as an infrared transparent top layer in spectrally engineered envelopes. Peng et al. developed a colored low-emissivity coating using a spray method, employing a bilayer configuration consisting of an infrared transparent top layer and a functional base layer (Fig. 12f, g) [210]. In this architecture, the infrared transparent layer preserves mid-infrared transmission across the coating surface, while the underlying layer provides device-level spectral tailoring that suppresses radiative heat leakage from the interior environment, which is beneficial for heating scenarios in cold conditions. Accordingly, the role of the MIR transparent component is to provide an optically transparent and environmentally robust encapsulation layer that enables radiative coupling through the exterior coating without introducing substantial mid-infrared absorption.

Collectively, these examples indicate that high *T*_MIR_ is the enabling materials requirement for infrared transparent windows and encapsulation layers in radiative heating-related architectures, whereas additional functional layers, when present, primarily provide device-level spectral tailoring while the *T*_MIR_ transparent component determines the MIR radiative throughput through the protected interface.

#### Dynamic Radiation Regulation

Dynamic radiation regulation involves the real-time modulation of thermal emissivity and *T*_MIR_ to adaptively control heat exchange with the environment under varying external conditions [211–213]. High *T*_MIR_ materials are integral to this function, serving either as passive transmission layers or as active components whose optical properties can be tuned via external stimuli such as temperature, electrical bias, or phase transitions [214, 215]. Their inherent transparency in the MIR ensures that changes in the emissive or reflective states of underlying functional layers such as thermochromic or electrochromic materials are not spectrally hindered, thereby maintaining the efficiency and responsiveness of the thermal regulation mechanism [216–218]. By enabling precise control over radiative energy flow while minimizing optical loss, high *T*_MIR_ materials provide a critical foundation for next-generation adaptive thermal systems that demand both spectral selectivity and environmental adaptability [219–221].

**Fig. 13:**
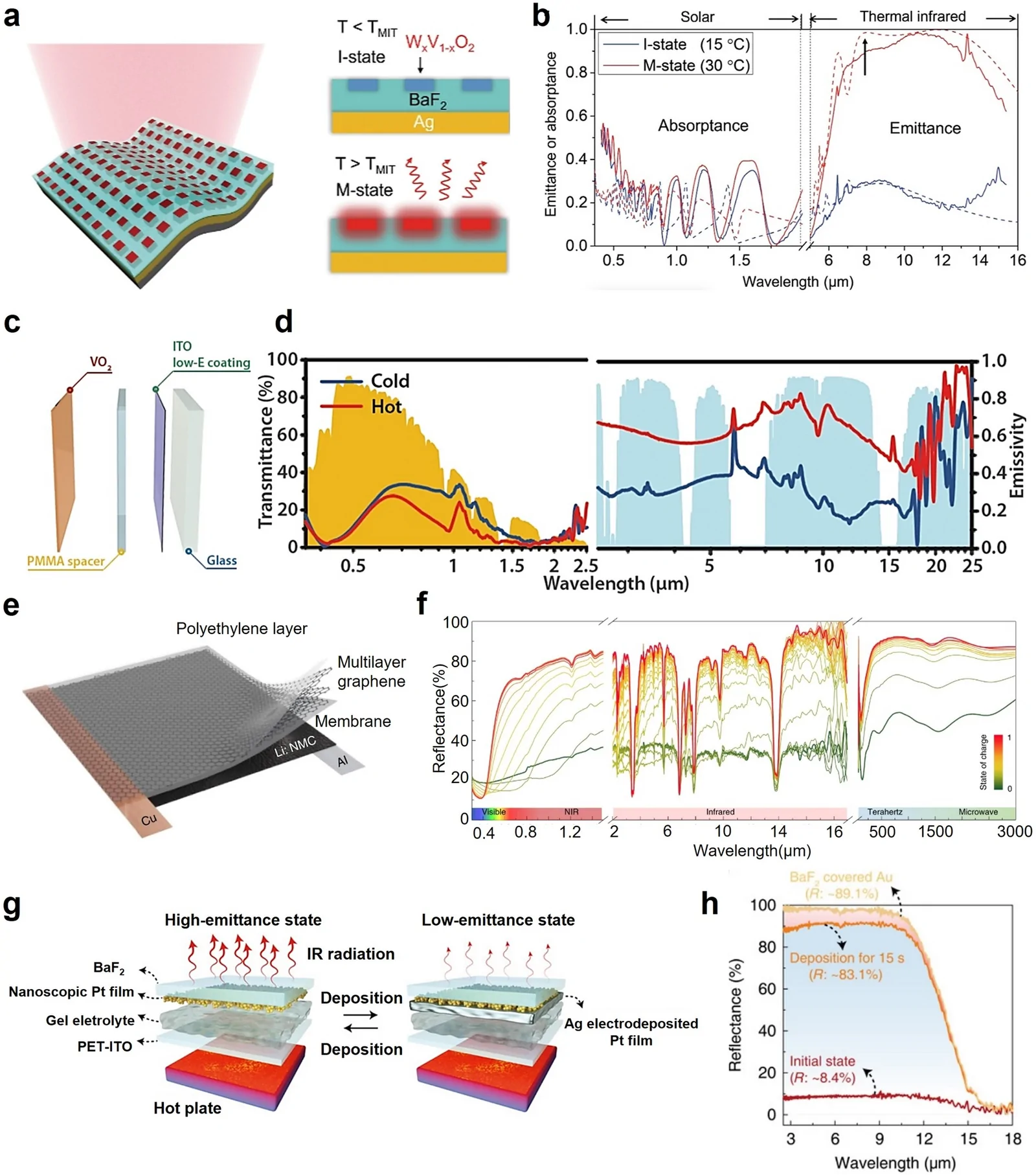
Applications of high *T*_MIR_ materials in dynamic radiation regulation**.** **a** Schematic diagram of the structure of a temperature-adaptive radiative coating. **b** Solar spectral absorptance and partial thermal spectral emissivity of temperature-adaptive radiative coatings at low and high temperatures [43]. Copyright 2021, The American Association for the Advancement of Science. **c** Schematic diagram of the structure of the radiation-cooled adaptive sample [34]. Copyright 2021, The American Association for the Advancement of Science. **d** Comparison of the spectra of radiation-cooled samples with the standardized AM1.5 global solar spectrum (yellow shading) and the LMIR atmospheric transmission window (blue shading). **e** Schematic diagram of the experimental setup. **f** Reflectance spectra of visible light, infrared, terahertz, and microwaves at different doping levels (charge states) [223]. Copyright 2021, Springer Nature. **g** Schematic diagrams of the nanometer-scale Pt thin-film electrodeposition device structure before (left) and after (right) electrodeposition. **h** Total infrared reflectance spectra of a 3 nm Pt/BaF_2_ substrate before and after (15 s) silver deposition in a three-electrode electroplating system [224]. Copyright 2020, The American Association for the Advancement of Science

BaF_2_ has been established as an effective dielectric layer for modulating the emissivity of VO_2_ in response to temperature fluctuations, thereby advancing dynamic radiative modulation technologies. Tang et al. introduced a temperature-adaptive radiation coating, where blocks of W_*x*_V_1-*x*_O_2_ thin film were integrated into a 2D lithographic pattern array and deposited onto a silver substrate coated with BaF_2_ dielectric layer, as depicted in Fig. 13a [43]. Below the critical temperature, W_*x*_V_1-*x*_O_2_ exhibits notable infrared transparency within the 8–13 μm atmospheric spectral window, maintaining its insulating properties. This transparency reduces infrared radiation absorption in this spectral range, thereby enhancing infrared reflection. Conversely, above the critical temperature, W_*x*_V_1-*x*_O_2_ undergoes a transition to a metallic state, resulting in increased absorption within the atmospheric spectral window. Furthermore, the researchers conducted a spectral analysis of this temperature-adaptive radiation coating across solar and far-infrared spectra (Fig. 13b). The results reveal that W_*x*_V_1-*x*_O_2_ exhibits an emissivity of approximately 0.20 in the insulating state and around 0.90 in the metallic state. This highlights the effectiveness of incorporating a BaF_2_ dielectric layer to achieve temperature-responsive modulation of W_*x*_V_1-*x*_O_2_ emissivity.

To achieve dynamic modulation of infrared emissivity, Wang and colleagues employed spin-coating technology to integrate PMMA, VO_2_, and indium tin oxide, constructing a Fabry–Perot cavity [34]. They specifically designed and fabricated a thermochromic window consisting of a VO_2_/spacer/low-emissivity stack, illustrated in Fig. 13c, to create the Fabry–Perot resonator. Vanadium dioxide nanoparticles dispersed within the PMMA spacer were applied via spin coating, enabling precise control over both solar transmittance and radiative cooling properties. Additionally, the spectral characteristics of this thermochromic window were thoroughly assessed (Fig. 13d). At 20 °C, the emissivity of the multilayer structure in the far-infrared spectrum was 0.21; however, this value increased significantly to 0.61 when the temperature exceeded 60 °C. These results demonstrate a marked reversal in emissivity with temperature variations, underscoring the unique dynamic modulation capability of the device [222].

In the field of electro-optic devices, PE serves a crucial role as a protective layer to maintain consistent optical modulation across the entire electromagnetic spectrum. Recently, Ergoktas et al. introduced an innovative graphene-based electro-optic device that showcases broad-spectrum optical modulation capabilities spanning from visible light to microwave frequencies [223]. This device utilizes multilayer graphene as both the anode and active optical material, capitalizing on improved optical accessibility facilitated by a transparent protective layer. To achieve this, as depicted in Fig. 13e, researchers employed layered configurations of graphene sheets and enclosed the device within a low-density PE bag. This encapsulation exhibits optical transparency exceeding 90% across the spectrum from visible light to microwave wavelengths, with selective absorption confined to the infrared region. Such airtight packaging is essential for ensuring the stability and reliable operation of the device. Additionally, to evaluate the performance of the device under various doping levels and across different spectral bands, researchers conducted reflectance spectroscopy measurements **(**Fig. 13f). The results confirm the capability of the device to modulate reflectance within the non-ionizing segment of the electromagnetic spectrum.

BaF_2_, when used as a substrate, significantly enhances the infrared absorptivity of materials, thereby enabling precise control over infrared emissivity. This ability to fine-tune emissivity is vital for applications such as adaptive thermal camouflage and other advanced optical technologies. For instance, Li et al. developed an adaptive thermal camouflage device using nanostructured Pt films, which are known for their high infrared absorption and partial transmission characteristics [224]. Even without electroplating treatment, these films exhibit increased emissivity. By applying a deposition voltage, silver gradually deposits onto the surface of the nanostructured Pt film, forming an Ag-deposited Pt film. This electroplating process progressively alters the infrared absorption and transmission properties of the nanostructured Pt film, converting it into an infrared reflector and effectively controlling low emissivity (Fig. 13g). Furthermore, the infrared transparent BaF_2_ substrate not only provides structural integrity but also reduces average reflectance across the 3–14 μm wavelength range by approximately 4.7%–15% through effective refractive index matching. This enhancement further augments the infrared absorption capabilities of the nanostructured Pt film, thereby facilitating low emissivity characteristics under electroplating conditions. For example, as demonstrated in Fig. 13h, utilizing a Pt/BaF_2_ substrate with a 3 nm thickness and applying − 2.2 V for 15 s during electroplating increased the average infrared reflectance of the structure from about 8.4% to approximately 83.1%. This significant increase highlights the critical role of the infrared transparent BaF_2_ substrate in enhancing the infrared absorption properties of nanostructured Pt films.

**Fig. 14:**
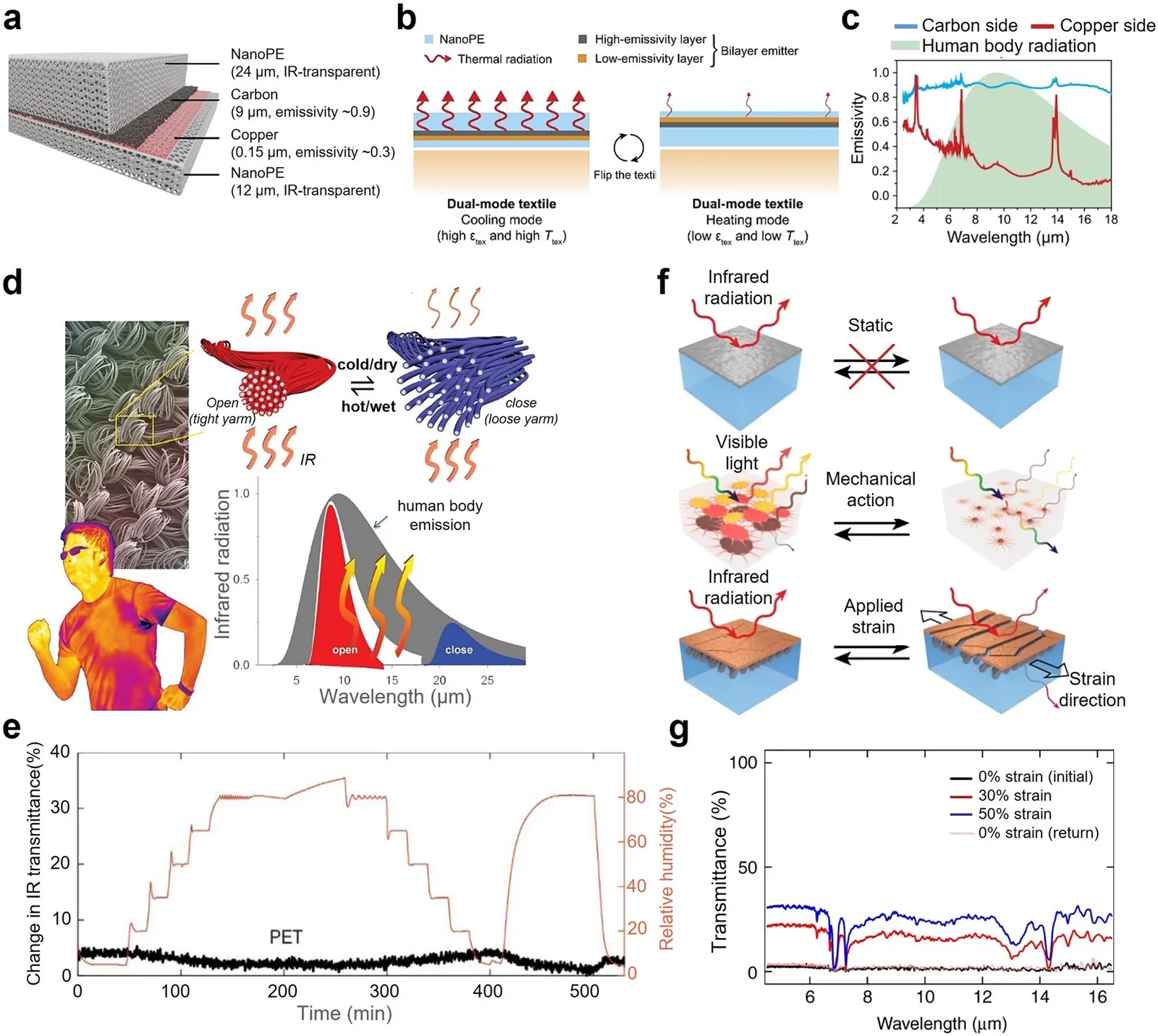
Applications of high *T*_MIR_ materials in dynamic radiation regulation. **a** Schematic diagram of the structure of a dual-mode textile with a bilayer structure. **b** Schematic diagram of the operating principle of dual-mode textiles. **c** Emissivity of carbon and copper coatings [225]. Copyright 2017, The American Association for the Advancement of Science. **d** Design principles of infrared gating textiles. **e** Infrared response of traditional polyethylene terephthalate fiber textiles [226]. Copyright 2019, The American Association for the Advancement of Science. **f** Comparison of the working mechanism of bioinspired thermal-regulating composites with space blankets. **g** Total TMIR spectra of the composite material at 0, 30, and 50 strain [227]. Copyright 2019, Springer Nature

Variations in the thickness of the protective PE layer play a critical role in the infrared emissivity of devices, enabling dynamic control of their radiative properties. Liu et al. showcased a dual-emitter configuration featuring an infrared transparent nanostructured PE layer (Fig. 14a) [225]. By modifying the thickness of this PE layer, the radiative characteristics can be dynamically adjusted. This nanostructured PE layer comprises a 24 μm-thick carbon side and a 12 μm-thick copper side. In cooling mode, as depicted in Fig. 14b, the high-emissivity carbon layer is oriented outward, while the thin nanolayer between the emitter and the skin enhances thermal conduction, raising the emitter’s temperature. This combination of high outward emissivity and minimal distance between the emitter and the skin results in a high heat transfer coefficient, thus optimizing the textile for cooling. Conversely, when the textile is inverted, the low-emissivity copper layer faces outward, increasing the distance between the emitter and the skin, reducing thermal conductivity, and shifting the textile into heating mode. This asymmetry in emissivity and nanocoating thickness yields two distinct heat transfer coefficients. As illustrated in Fig. 14c, the carbon side shows an emissivity of approximately 0.8 to 1.0 in the 2–18 μm wavelength range, with a weighted average emissivity of 0.894 at 33 °C, closely matching human radiation. In contrast, the copper side exhibits a significantly lower emissivity, with a weighted average of 0.303.

Significant advancements in the dynamic regulation of infrared emissivity have also been achieved using ultra-fibers in textiles, enabling adaptive thermal management. Zhang et al. introduced an infrared-adaptive textile, where each yarn consists of ultra-fiber bundles (Fig. 14d) [226]. In high-temperature or high-humidity environments, the yarns collapse, causing the constituent elements on adjacent fibers to converge and induce resonant electromagnetic coupling. This effect alters the textile’s emissivity, enhancing heat exchange efficiency by better matching it with human thermal radiation. In low-temperature or low-humidity environments, the yarns return to their original state, reducing heat dissipation. This infrared-adaptive textile can effectively regulate over 35% of infrared radiation in response to changes in skin relative humidity. In contrast, the control sample made from commercially available polyethylene terephthalate does not exhibit infrared tunability with humidity. This difference underscores the critical role of the superfiber-driven mechanism in achieving the observed infrared gating effect (Fig. 14e). The use of infrared transparent polymers as substrates is crucial for controlling infrared emissivity. Drawing inspiration from the static infrared reflection mechanism in space blankets and the dynamic color-changing ability in squid skin, Leung et al. developed an innovative composite material [227]. This composite includes a flexible and stretchable infrared transparent polymer substrate and an array of columnar nanostructures made of infrared-reflective metal domains (Fig. 14f). Initially, without mechanical manipulation, the densely packed nanostructures anchor the infrared-reflective metal regions to the bottom surface of the substrate, reflecting almost all incident infrared radiation. However, when mechanically manipulated, the metal domains disperse, partially exposing the stretched polymer substrate and allowing significant amounts of infrared radiation to pass through the composite. This dynamic regulation underscores the critical role of the infrared transparent polymer substrate. The *T*_MIR_ of the composite under varying stress conditions, shown in Fig. 14g, reveals a nonlinear relationship between the composite’s reflectance, transmittance, and applied strain.

### Optical and Photonic Applications

The systematic categorization of high *T*_MIR_ materials (Table 1) provides their structural characteristics and optical properties across the MIR wavelength spectrum, which establishes foundations for advanced photonic systems requiring precise MIR radiation manipulation, particularly in imaging/sensing architectures and laser engineering. For example, porous silicon enables dynamic modulation for high-speed photonic devices, while Er/Yb co-doped ceramics demonstrate superior performance in thermal sensing applications. Their operational efficacy primarily stems from exceptional spectral selectivity and broadband transmittance characteristics. Notably, nonlinear Opt. Mater. such as chalcogenide glasses and fluoride crystals with high *T*_MIR_ values facilitate efficient frequency conversion processes. Complementary to these, saturable absorbers like CrOCl exhibit critical functions in pulsed laser systems. The classification framework enables rational material selection through systematic correlation between intrinsic material properties and device operational requirements. This section focuses on design principles, providing guidelines for targeted material design to achieve specific optical functionalities.

#### MIR Imaging and Sensing

The advancement of MIR imaging and sensing technologies critically relies on the development of materials with tailored optical properties. Recent breakthroughs in polymeric, inorganic, and hybrid systems have enabled control over IR transparency, modulation dynamics, and luminescent sensing capabilities [228–230]. By integrating material design, surface engineering, and dynamic modulation strategies, longstanding challenges in broadband imaging efficiency, environmental durability, ultra-fast signal control, and precision thermometry are poised for resolution [231, 232].

**Fig. 15:**
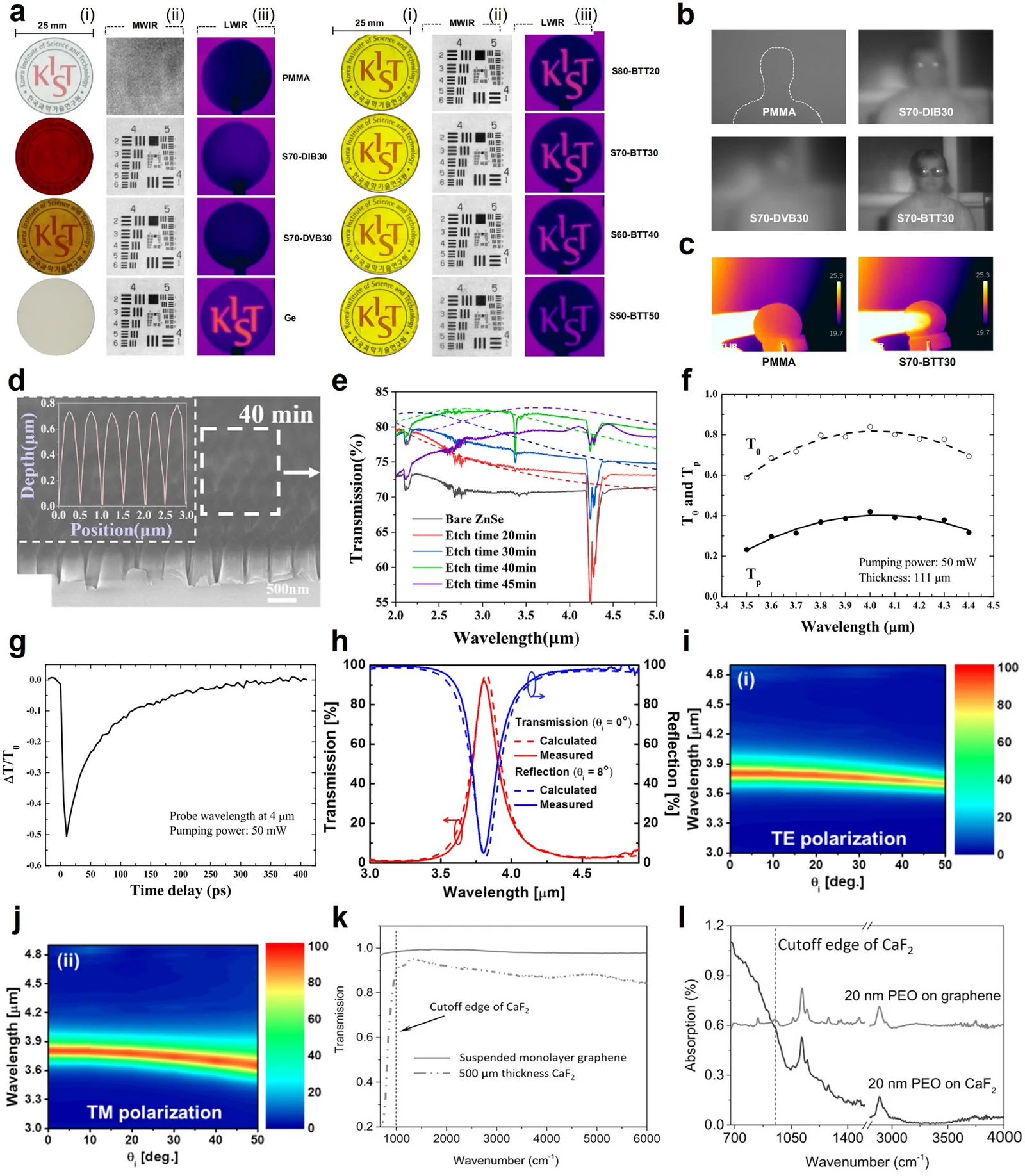
Applications of high *T*_MIR_ materials in imaging and sensing.** a** Comparative analysis of window materials: (i) Photographs of various polymer and Ge windows; (ii) MWIR imaging of USAF 1951 resolution target through corresponding materials; (iii) MIR imaging of patterned PMMA mask demonstrating transparency hierarchy across material series (PMMA, S70-DIB30, S70-DVB30, Ge, S80-BTT20, S70-BTT30, S60-BTT40, and S50-BTT50, top to bottom). **b** NIR images of a female subject captured through 1 mm-thick polymer windows: PMMA, S70-DIB30, S70-DVB30, and S70-BTT30. **c** MIR images of a human finger acquired through 1 mm-thick PMMA and S70-BTT30 windows [139]. Copyright 2023, Springer Nature. **d** SEM images of cross-sectional views of MENS etching for 40 min. **e** FTIR transmittance spectra measurement of the fabricated MENS with different etching times [118]. Copyright 2023, Elsevier. **f** Spectral dependence of the transmittance of the 111 μm-thick sample at a pump power of 50 mW. **g** Fractional transmittance change at 4.0 μm and the 50 mW pump power as a function of the delay time [99]. Copyright 2016, Springer Nature. **h** Calculated and measured spectra of transmission (*θi* = 0°) and reflection (*θi* = 8°) of the filter for the unpolarized incidence. Measured transmission spectra in terms of the angle of incidence (*θi*), **i** for TE polarizations and **j** for TM polarizations [232]. Copyright 2017, The Optical Society. **k** Comparison of the transmission FTIR spectra of a suspended monolayer of graphene and a 500-μm-thick CaF_2_ substrate. The black dotted vertical line indicates the CaF_2_ cutoff edge. **l** IR absorption spectra of 20 nm PEO coated onto a four-layer graphene substrate or a 500 μm CaF_2_ substrate [95]. Copyright 2017, John Wiley and Sons

Lee et al. introduced sulfur-rich poly(S-r-BTT) copolymers as multifunctional optical platforms through innovative inverse vulcanization synthesis employing elemental sulfur and a symmetric thiol cross-linker (BTT) [139]. These copolymers demonstrated high MIR transparency (> 60% transmittance at 1 mm thickness), rendering them highly suitable as monolithic imaging substrates for advanced thermal systems. In MWIR imaging trials using USAF 1951 targets, the S80-BTT20 copolymer demonstrated outstanding resolving capability compared to both conventional PMMA and early-generation sulfur copolymers like S70-DIB30, which were hindered by resolution loss due to scattering effects (Fig. 15a). Beyond MIR applications, the copolymer’s broad-spectrum functionality was exemplified through simultaneous NIR (850 nm) facial imaging (Fig. 15b) and MIR thermographic finger visualization (Fig. 15c) through S70-BTT30 windows. This dual-band capability stems from optimized sulfur content (> 50 wt%), which concurrently achieves an ultra-high refractive index (n = 1.94–2.00 across 637–1549 nm) for enhanced light collection and thermal stability (T_g_ = 100.14 °C for S50-BTT50) for high-temperature operation. Furthermore, the material system integrates spectral filtering, environmental protection, and light guidance into a single layer, eliminating the need for traditional optical stacks.

Huang et al. developed a ZnSe MIR transparent substrate through nanostructure engineering for advanced optical platforms [118]. The parabolic moth-eye nanostructures (period = 500 nm, height = 740 nm) shown in Fig. 15d were fabricated via polystyrene nanosphere lithography and reactive ion etching, creating a monolithic antireflective interface that enhanced ZnSe’s native infrared transparency. As quantified in Fig. 15e, this architecture achieved 81.76% average transmittance across 2–5 μm compared to 71.28% for flat ZnSe. Additionally, the nanostructure geometry enabled environmental protection through Cassie–Baxter state hydrophobicity (114° contact angle vs. 71° for bare ZnSe), effectively repelling moisture condensation that typically degrades infrared sensor accuracy in humid environments. These material-level enhancements in photon collection efficiency and surface robustness improved sensor reliability under operational stresses.

The unique dual functionality of porous silicon (pSi) as both a passive spectral filter and an active modulator in the MWIR regime highlights its potential for advanced infrared imaging and sensing applications. Park et al. demonstrated that freestanding pSi membranes exhibit a natural transparency window with 84% transmittance at 4 μm, making them intrinsically suited as bandpass filters for MWIR radiation. Surface-bound molecular vibrations (Si–OH, Si–H) create flanking absorption bands, facilitating out-of-band rejection [99]. Beyond its passive properties, pSi also functions as an active modulation platform. Under 800 nm femtosecond pumping (50 mW, 4.8 mJ cm^−2^), pSi achieves 50% transmittance modulation (defined as T_o_-T_p_, where T_p_ is the excited states) across the entire MWIR window (Fig. 15f), with wavelength-independent attenuation attributed to free carrier absorption. As shown in Fig. 15g, time-resolved measurements revealed subpicosecond excitation rise (instrument-limited) and 66 ps recovery (1/e decay), corresponding to a 15 GHz modulation bandwidth. The material’s static transparency ensures baseline transmission for thermal imaging, while dynamic modulation enables time-gated detection or spectral filtering. This dual passive/active operation paradigm distinguishes pSi from conventional bulk materials, which are often constrained by static opacity or fixed bandgaps, and positions it as a cornerstone for adaptive optics in environmental LiDAR (light detection and ranging), multispectral surveillance, and dynamic scene projection.

Im and Lee designed a high-performance MIR filter through precision stacking of Ge and silicon monoxide (SiO) layers in a cascaded etalon configuration [125]. This dielectric architecture achieved 92% peak transmission at 3.8 μm wavelength with suppressed sideband interference across the 3–5 μm spectral range, enabling precise molecular fingerprinting critical for infrared chemical sensing (Fig. 15h). The Ge/SiO multilayer system demonstrated exceptional angular independence, maintaining spectral shifts below 0.1 μm even at 50° incidence angles (Fig. 15i, j). Hu et al. developed suspended graphene as an ultra transparent MIR interface [95]. This atomically thin platform exhibited > 97.5% broadband transmittance across a 2.5—25 μm range, effectively eliminating substrate absorption artifacts inherent in traditional CaF_2_ windows (Fig. 15k). When analyzing a 20 nm polyethylene oxide (PEO) film, graphene’s minimal spectral distortion revealed six previously undetectable vibrational modes compared to CaF_2_ measurements, enabling high-fidelity detection of weak molecular signals and showcasing remarkable sensitivity for nanoscale analyte characterization (Fig. 15l).

#### MIR Laser Systems

MIR transparent materials have emerged as indispensable components in laser systems by unifying broadband transparency, enhanced nonlinearity, and high laser durability [233, 234]. These materials facilitate the efficient transmission of pump wavelengths (*e.g.*, 1.06–2 μm) without parasitic absorption while simultaneously enabling coherent MIR output generation through frequency conversion [21]. In doing so, they function as multifunctional interfaces that bridge conventional laser technologies with emerging MIR applications. Through strategic structural engineering, such as non-centrosymmetric frameworks, tailored bandgaps, and covalent anionic networks, the longstanding incompatibility between high nonlinear coefficients and broadband MIR transparency can be effectively overcome [235]. This dual functionality, as both transmissive substrates and nonlinear-active media, underpins transformative advancements in environmental LiDAR, surgical lasers, and defense technologies, positioning MIR transparent materials as vital enablers of high-power, wavelength-agile photonic systems [236].

**Fig. 16:**
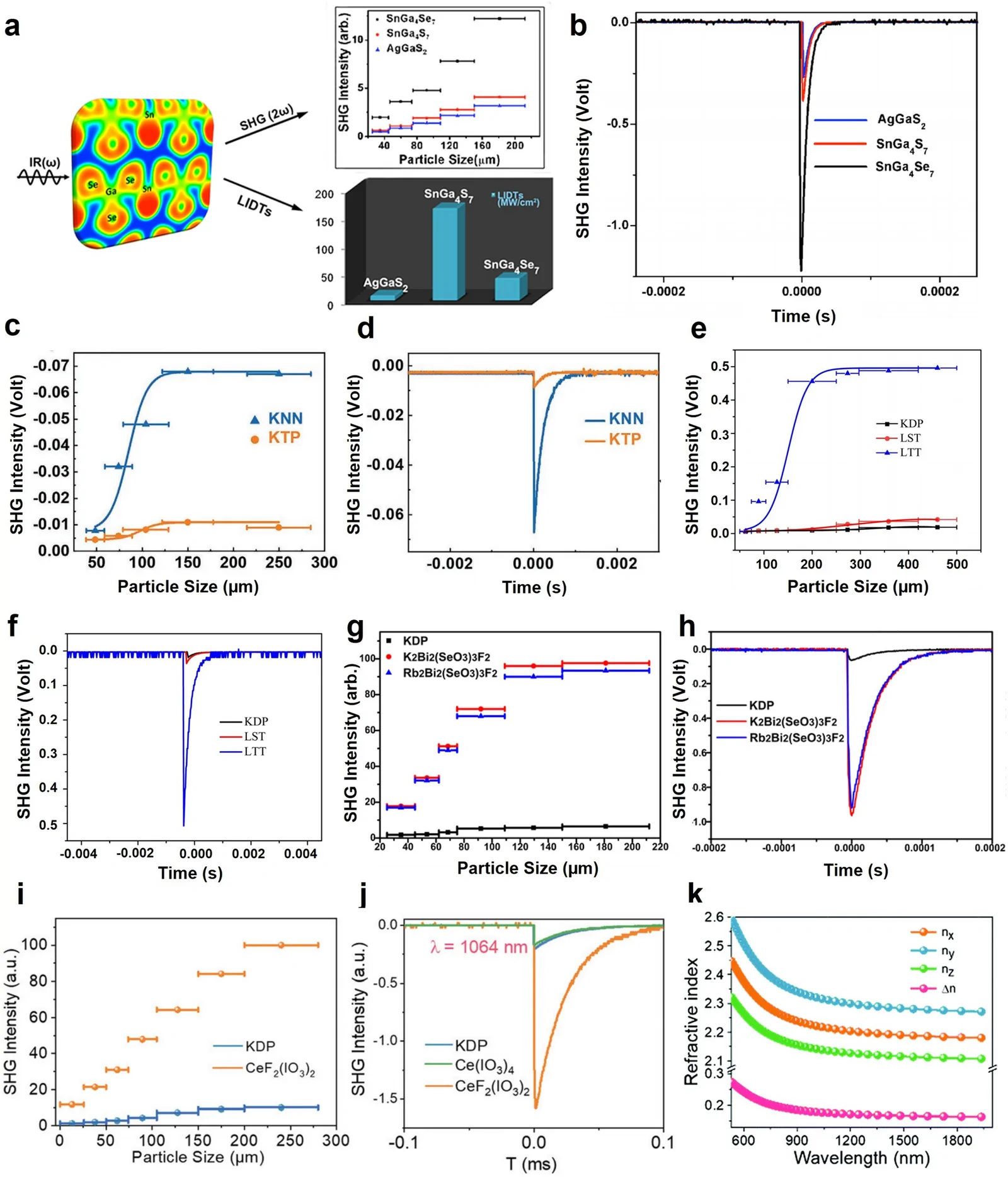
Applications of high *T*_MIR_ materials in the laser system. **a** Phase-matching curves, i.e., particle size vs SHG response for SnGa_4_S_7_ and SnGa_4_Se_7_ with AgGaS_2_ as a reference and LIDTs. **b** Oscilloscope traces of SHG signals for SnGa_4_S_7_ and SnGa_4_Se_7_ with AgGaS_2_ as a reference at a particle size of 150–210 μm [115]. Copyright 2014, American Chemical Society. **c** Phase-matching curves for KNN and KTP. **d** Oscilloscope traces of the SHG signals for KNN and KTP in the same particle size of 250–270 μm [120]. Copyright 2022, John Wiley and Sons. **e** Phase-matching curve for Li_2_MTeO_6_ (M = Ti, Sn). **f** Oscilloscope traces of the SHG signals for Li_2_MTeO_6_ (M = Ti, Sn) and KDP in the same particle size of 420–500 μm [123]. Copyright 2021, John Wiley and Sons. **g** Measured SHG intensities of A_2_Bi_2_(SeO_3_)_3_F_2_ (A = K, Rb) under 1064 nm laser radiation at room temperature. **h** Oscilloscope traces of the SHG signals of A_2_Bi_2_(SeO_3_)_3_F_2_ (corresponding particle size range of 150–212 μm) [128]. Copyright 2020, American Chemical Society. **i** Phase-matching curves of CeF_2_(IO_3_)_2_ under 1064 nm laser radiation. **j** Oscilloscope traces of the SHG responses of Ce(IO_3_)_4_ and CeF_2_(IO_3_)_2_ (105–150 mm). **k** Calculated wavelength-dependent refractive indices and birefringence of CeF_2_(IO_3_)_2_ [130]. Copyright 2021, Royal Society of Chemistry

Chalcogenides capitalize on their inherent MIR transparency windows (0.40–25 μm for S, 0.49–25 μm for Se) to operate as dual-purpose optical substrates, supporting efficient photon transport and nonlinear interactions across critical atmospheric windows. Luo et al. demonstrated SnGa_4_Q_7_ (Q = S, Se) as high-performance MIR transparent platforms for nonlinear optical frequency conversion [115]. As shown in Fig. 16a, these compounds exhibit type I phase matching with second-harmonic generation (SHG) intensities scaling linearly with particle size (150–210 μm). As MIR transparent phase-matching media, SnGa_4_S_7_ delivers an SHG efficiency approximately 1.3 times that of AgGaS_2_ (d36 = 13.7 pm V^−1^), while SnGa_4_Se_7_ achieves a record 3.8-fold enhancement (Fig. 16b). Their exceptional laser-induced damage thresholds (165.1 MW cm^−2^ for S, 40.0 MW cm^−2^ for Se) are directly attributed to the wide bandgaps (3.10 and 2.55 eV), a critical feature that mitigates MIR absorption-induced performance degradation under high-power operation. First-principles calculations confirm that these MIR transparent matrices facilitate synergistic dipole alignment among constituent units, yielding SHG coefficients (15.62 pm V^−1^ for S, and 26.71 pm V^−1^ for Se) that surpass those of single-phase systems through coordinated electron delocalization across the transparent lattice. This architecture establishes MIR transparent chalcogenides as multifunctional gain media, wherein optical transmission and nonlinear activity mutually reinforce one another, overcoming the conventional trade-off between bandgap and nonlinearity in IR laser design.

While chalcogenides demonstrate exceptional nonlinear performance, oxide-based systems offer complementary advantages in environmental stability. Yin et al. reported lead-free ferroelectric (K, Na)NbO_3_ (KNN) single crystals with a non-centrosymmetric *Amm*2 crystalline structure [120], enabling type I phase matching under 1064 nm excitation. The SHG efficiency scales linearly with particle sizes up to > 150 μm, and the intrinsic MIR transparency spanning 0.37–8 μm facilitates low-loss optical transmission across the critical 3–5 μm atmospheric window (Fig. 16c). KNN thereby operates as a dual-function nonlinear optical (NLO) medium, serving as both a broadband transmitter and frequency converter. Notably, comparative particle size analysis (250–270 μm) reveals that KNN’s SHG efficiency surpasses potassium titanyl phosphate (KTP) by a factor of 7.6 while maintaining exceptional MIR transparency (Fig. 16d). Furthermore, its laser damage threshold of 98.9 MW cm^−2^ positions KNN as a robust gain medium for high-power applications, offering greater optical resilience and environmental stability than traditional chalcogenides such as AgGaS_2_ and ZnGeP_2_. This performance profile stems from the synergistic combination of polar-aligned NbO_6_ octahedra and alkali cation ordering, which enhances nonlinear polarization without introducing heavy metal absorption losses.

Building on oxide frameworks, tellurate-based systems introduce structural versatility that accommodates both frequency conversion and thermal management. For example, Du et al. developed non-centrosymmetric Li_2_MTeO_6_ (M = Ti, Sn) crystals with inherent MIR transparency for laser applications [123]. Both variants (LTT and LST) crystallize in polar *Pnn*2 symmetry, achieving type I phase matching at 1064 nm with broadband transparency spanning 0.38–6.86 μm, which fully covers the 3–5 μm atmospheric window. Remarkably, LTT demonstrates a record SHG efficiency reaching 26 times that of KH_2_PO_4_ (KDP) at particle sizes of 420–500 μm, outperforming LST at 2.5 times KDP and established oxides such as LiNbO_3_ (Fig. 16e, f). This performance divergence originates from LTT’s compressed Ti–O bonding geometry (1.931–2.029 Å), which induces enhanced local polarization within the TiO_6_/TeO_6_ octahedral network compared to LST’s Sn-containing configuration. By achieving phase-matchable operation at Nd:YAG wavelengths with damage thresholds exceeding 550 MW cm^−2^ (LTT) and 672 MW cm^−2^ (LST), these dual-purpose crystals resolve the traditional efficiency–power trade-off inherent to chalcogenide systems, offering direct compatibility with existing solid-state laser architectures.

Parallel developments in fluorinated architectures reveal new pathways for balancing nonlinear efficiency with optical durability. Shi et al. designed a high-performance nonlinear optical platform for MIR laser engineering based on alkali metal bismuth selenite fluorides A_2_Bi_2_(SeO_3_)_3_F_2_ (A = K, Rb) [128]. Both compounds display type I phase-matching behavior under 1064 nm excitation, attaining SHG efficiencies of 15.0 times that of KDP for the K analog and 14.4 times that of KDP for the Rb analog, representing the highest values reported among phase-matchable metal selenites (Fig. 16g, h). Their comprehensive transparency window (0.33–6.70 μm) fully encompasses the crucial 3–5 μm atmospheric transmission band, enabling dual-wavelength operation across visible to infrared spectral regions. The potassium variant exhibits superior SHG intensity (0.40 of AgGaS_2_ at 2.05 μm) and exceptional laser-induced damage thresholds (81.3 times that of AgGaS_2_), a synergistic effect arising from its wide experimental bandgap (~ 3.7 eV) and strategically aligned dipole moments within the one-dimensional [Bi_2_O_9_F_2_] chains. Density functional theory calculations reveal that the non-centrosymmetric configuration of [BiOₓFᵧ] and SeO_3_ units amplifies macroscopic polarization, with hybridized Bi(6*p*)/O(2*p*)/F(2*p*)/Se(4*p*) orbitals predominantly dictating the nonlinear optical response. The thermal stability exceeding 400 ℃ and scalable crystal growth processes further substantiate their practical applicability for frequency conversion in high-power MIR laser systems, particularly in atmospheric sensing and molecular spectroscopy implementations. While the rubidium analog shows moderately lower SHG efficiency (0.36 relative to AgGaS_2_), its maintained damage resistance (48.8 relative to AgGaS_2_) highlights the compositional flexibility of this system for tailoring material properties to specific laser requirements.

The incorporation of heavy metal cations with optimized anion coordination further expands the design landscape of MIR transparent materials. Wu et al. developed fluorinated cerium iodates as advanced platforms for MIR nonlinear optical applications, where CeF_2_(IO_3_)_2_ serves as an efficient frequency conversion medium [130]. CeF_2_(IO_3_)_2_ exhibits dual functionality within its characterized MIR transparency window (0.43–6.46 μm), serving simultaneously as a phase-matching medium and nonlinear-active component for optical parametric oscillators. Structural fluorination induces asymmetric [CeO_6_F_3_] polyhedra, enhancing local dipole alignment while preserving MIR transparency across the critical 3–5 μm atmospheric window. Benefiting from an MIR transparent lattice with strengthened nonlinear susceptibility, CeF_2_(IO_3_)_2_ delivers an SHG efficiency roughly eightfold that of KDP, supporting high-efficiency frequency conversion with negligible parasitic absorption (Fig. 16i, j). A widened bandgap (2.90 eV) ensures intrinsic laser damage resistance while maintaining MIR transmission fidelity. Polarized light microscopy reveals 0.212 birefringence, confirming phase-matching capability across the operational MIR spectrum (Fig. 16k). Coupled with thermal stability up to 430 °C, this material demonstrates remarkable resilience under high-power thermal loads.

**Fig. 17:**
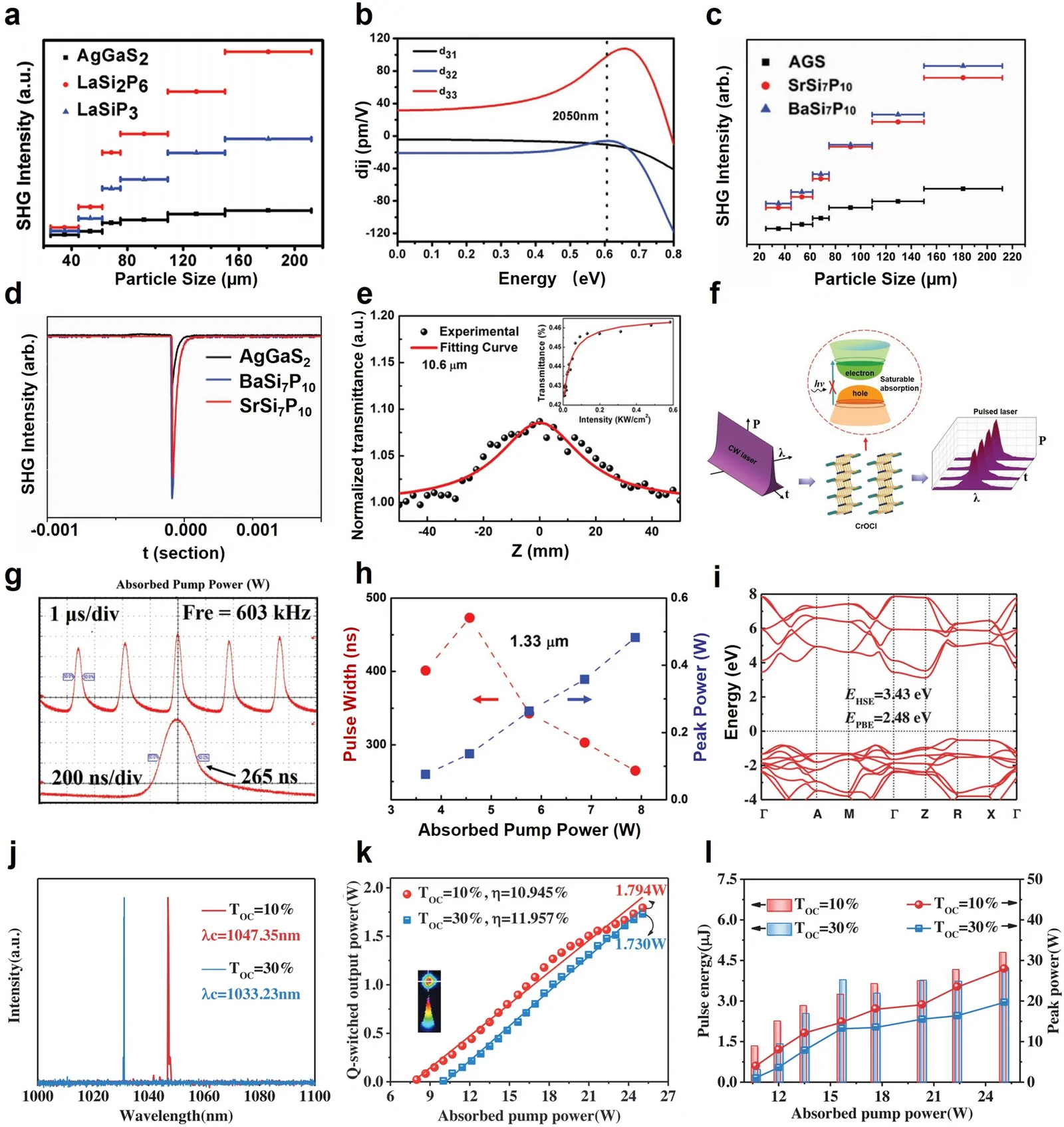
Applications of high *T*_MIR_ materials in the laser system. **a** Powder SHG measurements of LaSiP_3_ and LaSi_2_P_6_ at 2050 nm. **b** Calculated SHG coefficients of LaSi_2_P_6_ [132]. Copyright 2021, John Wiley and Sons. **c** Measured SHG intensities of BaSi_7_P_10_, SrSi_7_P_10_, and AgGaS_2_ under a 2.05 μm laser radiation at room temperature. **d** Measured SHG signals of BaSi_7_P_10_, SrSi_7_P_10_, and AgGaS_2_ with a 150–210 μm particle size [237]. Copyright 2022, John Wiley and Sons. **e** OA Z-scan data and theoretical fitting of the CrOCl crystal at 10.6 μm. The insets show the variations in the normalized transmittance with the incident intensity. **f** Schematic diagram of Q-switched laser generation. **g** Corresponding pulse trains and single-pulse profiles at 1.33 μm. **h** Pulse width and single-pulse peak power at 1.33 μm [133]. Copyright 2020, John Wiley and Sons. **i** Band gap structure of BiOCl calculated by GGA-PBE and HSE06. **j** Output spectrum with different output couplers of Q-switched Yb:LuAG SCF laser using BiOCl. **k** Average output power versus the absorbed pump power, typical 2D beam profiles (inset). **l** Pulse energies and peak powers versus absorbed pump power of the Q-switched Yb:LuAG SCF laser using BiOCl [134]. Copyright 2022, John Wiley and Sons

Beyond conventional cation frameworks, rare earth pnictides introduce three-dimensional covalent networks for enhanced nonlinear responses. Sun et al. reported LaSiP_3_ and LaSi_2_P_6_ compounds with a three-dimensional phosphorus network as mid-to-far-infrared frequency conversion media [132]. These materials display phase-matching behavior under 2050 nm excitation, with SHG intensities reaching saturation at particle sizes of 150–200 μm (Fig. 17a). The three-dimensional phosphorus network in LaSi_2_P_6_ achieves a record nonlinear coefficient (d_33_ = 98.5 pm V^−1^), surpassing conventional materials like ZnGeP_2_ while maintaining comparable bulk dipole moments to its layered counterpart LaSiP_3_ **(**Fig. 17b). This enhanced performance stems from optimized π-electron delocalization within the covalent framework, which amplifies nonlinear responses without compromising structural integrity. Both materials uniquely combine broad infrared transparency (spanning critical atmospheric windows) with high laser damage thresholds (~ 3 relative to AgGaS_2_), thereby overcoming the durability limitations of traditional nonlinear optical crystals. The engineered birefringence (Δn = 0.24–0.25 at 2050 nm) intrinsic to these transparent materials ensures phase matchability across operational wavelengths, addressing a key challenge in practical laser systems.

The nonlinear performance of pnictide systems can also be enhanced by tuning the structural dimensionality. Zhao et al. utilized T2-type pnictides BaSi_7_P_10_ and SrSi_7_P_10_ MIR transparent crystals (transparency window: 3–25 μm) and achieved high phase-matched SHG performance under 2.05 μm excitation, with saturation intensities at 150–212 μm particle sizes delivering approximately 3.2-fold and 3.0-fold AgGaS_2_ efficiency, respectively (Fig. 17c, d) [237]. Their functionality as frequency-doubling media stems from the dual-T_2_ supertetrahedral architecture, where aligned [SiP₄] tetrahedra within [Si_7_P_16_] chains create polarization-enhanced channels through cooperative dipole alignment. The intrinsic MIR transparency enables photon transport across critical atmospheric windows while the exceptional laser damage thresholds (6.69–7.05 relative to AgGaS_2_) establish these materials as durable optical workhorses for high-power laser cavities. First-principles calculations reveal that the MIR transparent framework facilitates optimized charge transfer via two-coordinate phosphorus atoms, producing sufficient birefringence (0.077–0.112 @2050 nm) for phase matching without compromising optical transmission. This synergy positions T_2_-type pnictides as multifunctional MIR components, simultaneously serving as nonlinear-active media, broadband transmissive elements, and thermally robust optical interfaces for frequency conversion across the 3–25 μm spectral regime.

Shifting focus from laser frequency conversion, MIR transparent materials enable advanced photonic functionalities through nonlinear absorption engineering, such as broadband saturable absorbers. Wang et al. devised a broadband saturable absorber platform based on the MIR transparent CrOCl crystal, which features a transmission window of 0.8–18 μm [133]. Leveraging its layered architecture, CrOCl exhibits intensity-dependent transparency across atmospheric windows critical for MIR photonics. Z-scan measurements at 10.6 μm revealed a distinct saturable absorption response, with a nonlinear absorption coefficient of − 5.37 cm W^−1^, demonstrating the material’s functionality in the MIR regime (Fig. 17e). This MIR transparency enables passive Q-switching operations spanning wavelengths from 1.06 to 1.87 μm (Fig. 17f), achieving the shortest pulse widths of 265 ns, corresponding to peak powers of 0.48 W at 1.33 μm (Fig. 17g, h). The 10.6 μm absorption modulation highlights CrOCl’s unique capability to bridge the "MIR gap" between conventional laser wavelengths and molecular fingerprint regions, addressing the limitation of existing oxide crystals like BBO and KTP (< 4 μm). Furthermore, the CrOCl crystal’s van der Waals layered structure enhances photon–matter interactions across multiple IR windows while maintaining spectral fidelity. By combining broadband modulation capabilities (1.06–10.6 μm as per Z-scan characterization) with exceptional laser damage thresholds (> 463 MW cm^−2^ at 1.06 μm), CrOCl exemplifies MIR-optimized materials capable of wavelength-flexible modulation for laser systems requiring both broadband response (spectroscopy) and thermal stability (high-power processing).

Layered MIR transparent saturable absorbers also hold promise for wavelength-agile laser modulation platforms. Ma et al. reported a MIR transparent saturable absorber based on BiOCl crystals, optimized for watt-level passively Q-switched Yb: LuAG single-crystal fiber lasers [134]. As shown in Fig. 17i, BiOCl enables Q-switching through intracavity loss modulation, leveraging its broadband transparency (0.4–11 μm) and nonlinear saturable absorption characteristics. With an indirect bandgap of 3.3 eV, BiOCl exhibits intensity-dependent nonlinear responses critical for passive Q-switching, achieving modulation depths of 34% and 20.8% at 532 and 1030 nm, respectively. As shown in Fig. 17j, BiOCl induces a wavelength blue shift from 1049.22 to 1047.35 nm during Q-switching operations, attributed to ground-state depletion effects. The system achieves a maximum average power of 1.794 W at an absorbed pump power of 25.074 W, with a slope efficiency of 10.945%, demonstrating stable operation under high-intensity pumping (Fig. 17k). Pulse compression to durations of 171.2 ns and peak power amplification to 28.019 W further underscore BiOCl’s capacity for high-energy pulse generation, exemplifying its utility in high-intensity photonic applications (Fig. 17l).

## Conclusion

This review presents a comprehensive analysis of high *T*_MIR_ materials from a materials science and optical physics perspective, systematically establishing the structure–property–function relationships that govern MIR transparency across diverse material classes. The discussion begins with a rigorous examination of the fundamental physical principles that determine *T*_MIR_, including energy balance, complex refractive index behavior, the Beer–Lambert law, Fresnel reflection, and various attenuation mechanisms such as intrinsic absorption, scattering, and microstructural heterogeneity. These mechanistic insights lay the foundation for understanding how optical losses arise and how they can be strategically mitigated through material and interface engineering. By categorizing intrinsic high *T*_MIR_ materials such as oxides, Group VIA compounds, Group IVA elements, fluorochemicals, phosphides, oxyhalides, Prussian blue analogs, and polymers, this review highlights how the interplay of electronic band structure, phonon dynamics, and crystallographic symmetry governs spectral absorption and photon propagation within the 2.5–20 μm range. Parallel to these intrinsic systems, microstructural design strategies including subwavelength structuring, hierarchical porosity, refractive index grading, and hybrid interfaces have proven effective in further enhancing *T*_MIR_ by minimizing reflection and scattering losses. These approaches not only elevate the performance of already transparent materials but also enable transparency in systems previously limited by their intrinsic absorption. High *T*_MIR_ materials have shown wide-ranging functional significance in thermal management, including radiative cooling, heating, and dynamic infrared modulation, as well as in photonic applications such as MIR imaging, sensing, and laser systems. Collectively, these findings underscore that optimal MIR transparency arises not only from favorable chemical composition but also from precise, multiscale structural control informed by foundational optical principles.

Looking forward, the advancement of high *T*_MIR_ materials will rely on addressing several critical challenges and harnessing emerging opportunities in design, fabrication, and integration. On the fundamental level, future research should focus on deepening the understanding of phonon–photon interactions, lattice anharmonicity, and the role of electronic defects in MIR attenuation, aided by advanced spectroscopy and multiscale modeling. At the forefront of materials discovery, design strategies that integrate compositional complexity with structural symmetry breaking, including mechanisms like lone pair distortion, polarizable frameworks, and metastable phase stabilization, are poised to drive the development of next-generation broadband MIR transparent systems with multifunctional capabilities. In application-driven contexts, the development of hybrid platforms that synergize high *T*_MIR_ with nonlinear optical activity, luminescence tunability, or environmental adaptability will be essential for enabling intelligent photonic devices, energy-efficient thermal control, and robust sensing technologies. The integration of these materials into complex system architectures such as multilayer metastructures, flexible textiles, or adaptive filters will further require scalable processing methods, along with high thermal–mechanical stability and strong environmental resilience. Manufacturability remains constrained by stringent optical quality requirements, including thickness uniformity and suppression of pores, inclusions, and interface defects, which can increase processing complexity and reduce yield. Cost and environmental considerations, including precursor availability, processing energy intensity, and long-term stability, should be assessed alongside optical metrics. For integration with CMOS and flexible electronics, compatibility with thermal budgets, contamination control, patterning, adhesion, and mechanical reliability must be addressed. Finally, computational and data-driven discovery that couples first principles prediction with machine learning-based screening and inverse design can accelerate the identification of MIR materials that satisfy these practical constraints. Moreover, data-driven materials design, enabled by high-throughput screening and machine learning, holds promise for accelerating the identification of MIR materials with tailored optical signatures. In sum, high *T*_MIR_ materials represent a foundational enabler for the next generation of infrared technologies, and continued interdisciplinary exploration will be pivotal in transforming their scientific potential into transformative real-world applications.
